# Supplementary material for: XMAP: Cross-population fine-mapping by leveraging genetic diversity and accounting for confounding bias
Source: Nat Commun. 2023 Oct 28;14:6870. doi: 10.1038/s41467-023-42614-7 (PMC10613261; doi:10.1038/s41467-023-42614-7)
Supplement: Supplementary file 1 — Supplementary Information [file 41467_2023_42614_MOESM1_ESM.pdf]

# Supplementary Information of "XMAP: Cross-population fine-mapping by leveraging genetic diversity and accounting for confounding bias"

## Contents


**1 Supplementary Tables** **2**

**2 Supplementary Figures** **4**

**3 Supplementary Note** **46**

3.1 Simulations without polygenic effects and confounding bias . . . . . . . . . . . . 46

3.2 Simulations with misspecified genetic effects . . . . . . . . . . . . . . . . . . . . . 46

3.3 Compared methods . . . . . . . . . . . . . . . . . . . . . . . . . . . . . . . . . . 47

3.4 Derivation of the covariance of $\boldsymbol{\epsilon}_1$ and $\boldsymbol{\epsilon}_2$ . . . . . . . . . . . . . . . . . . . . . 47

3.5 The XMAP model accounting for sample structure . . . . . . . . . . . . . . . . . 50

3.6 Derivation of the variational EM algorithm of XMAP . . . . . . . . . . . . . . . 51

3.7 Adjustment to $z$-scores . . . . . . . . . . . . . . . . . . . . . . . . . . . . . . . . 55

# 1 Supplementary Tables

| | Cohort | Intercept | Intercept s.e. | per-SNP h2 | per-SNP h2 s.e. |
|---|---|---|---|---|---|
| **LDL-AFR** | GLGC | 1.066208 | 1.84E-02 | 3.88E-08 | 1.25E-08 |
| **LDL-EUR** | GLGC | 1.101591 | 1.47E-02 | 2.23E-08 | 5.97E-09 |
| **LDL-EUR** | UKBB | 1.094689 | 1.39E-02 | 1.93E-08 | 4.51E-09 |
| **LDL-EAS** | GLGC | 1.036577 | 7.17E-03 | 2.37E-08 | 4.50E-09 |
| **height-EUR** | UKBB | 1.658928 | 4.16E-02 | 7.60E-08 | 4.34E-09 |
| **height-EUR** | Sibship | 1.116069 | 1.17E-02 | 5.41E-08 | 3.36E-09 |
| **height-EAS** | Chinese | 1.069005 | 8.81E-03 | 8.45E-08 | 6.33E-09 |
| **height-EAS** | BBJ | 1.394916 | 2.41E-02 | 7.81E-08 | 5.31E-09 |
| **Lym-EUR** | UKBB | 1.236483 | 1.66E-02 | 3.00E-08 | 2.37E-09 |
| **Lym-EAS** | BBJ | 1.055371 | 8.18E-03 | 1.47E-08 | 1.96E-09 |
| **WBC-EUR** | UKBB | 1.250661 | 1.79E-02 | 2.85E-08 | 2.07E-09 |
| **WBC-EAS** | BBJ | 1.092382 | 8.88E-03 | 1.76E-08 | 1.93E-09 |
| **Neutro-EUR** | UKBB | 1.217628 | 1.55E-02 | 2.49E-08 | 2.33E-09 |
| **Neutro-EAS** | BBJ | 1.049925 | 7.98E-03 | 2.05E-08 | 2.91E-09 |
| **Mono-EUR** | UKBB | 1.240563 | 1.95E-02 | 3.40E-08 | 4.04E-09 |
| **Mono-EAS** | BBJ | 1.075889 | 8.16E-03 | 1.61E-08 | 2.71E-09 |
| **Eosino-EUR** | UKBB | 1.208816 | 1.78E-02 | 2.52E-08 | 2.37E-09 |
| **Eosino-EAS** | BBJ | 1.067848 | 8.74E-03 | 1.52E-08 | 2.77E-09 |
| **Baso-EUR** | UKBB | 1.113947 | 7.09E-03 | 3.21E-09 | 3.30E-10 |
| **Baso-EAS** | BBJ | 1.100899 | 9.22E-03 | 9.61E-09 | 3.77E-09 |
| **Plt-EUR** | UKBB | 1.27035 | 1.87E-02 | 4.62E-08 | 4.17E-09 |
| **Plt-EAS** | BBJ | 1.120054 | 9.69E-03 | 3.02E-08 | 4.30E-09 |
| **RBC-EUR** | UKBB | 1.257341 | 1.84E-02 | 3.53E-08 | 3.64E-09 |
| **RBC-EAS** | BBJ | 1.098981 | 9.41E-03 | 2.24E-08 | 3.19E-09 |
| **MCH-EUR** | UKBB | 1.176256 | 1.62E-02 | 4.62E-08 | 7.09E-09 |
| **MCH-EAS** | BBJ | 1.102186 | 9.43E-03 | 3.26E-08 | 5.78E-09 |
| **MCHC-EUR** | UKBB | 1.064449 | 9.01E-03 | 9.36E-09 | 1.20E-09 |
| **MCHC-EAS** | BBJ | 1.076843 | 8.91E-03 | 9.70E-09 | 2.09E-09 |
| **MCV-EUR** | UKBB | 1.202512 | 1.67E-02 | 4.60E-08 | 6.46E-09 |
| **MCV-EAS** | BBJ | 1.119433 | 1.03E-02 | 3.56E-08 | 5.76E-09 |
| **Hb-EUR** | UKBB | 1.21788 | 1.86E-02 | 3.32E-08 | 2.90E-09 |
| **Hb-EAS** | BBJ | 1.065907 | 8.12E-03 | 1.23E-08 | 1.45E-09 |

**Supplementary Table 1:** Estimates of LDSC intercepts and per-SNP heritabilities.

| | Cohort | n | p | Publication | Source |
|---|---|---|---|---|---|
| **LDL-AFR** | GLGC | 92,934 | 25,476,275 | doi.org/10.1038/s41586-021-04064-3 | http://csg.sph.umich.edu/willer/public/glgc-lipids2021/ |
| **LDL-EUR** | GLGC | 664,450 | 35,328,891 | doi.org/10.1038/s41586-021-04064-3 | http://csg.sph.umich.edu/willer/public/glgc-lipids2021/ |
| **LDL-EUR** | GLGC (non UKB) | 85,785 | 27,323,411 | doi.org/10.1038/s41586-021-04064-3 | http://csg.sph.umich.edu/willer/public/glgc-lipids2021/ |
| **LDL-EUR** | UKBB | 343,621 | 12,515,778 | doi.org/10.1038/s41586-018-0579-z | https://nealelab.github.io/UKBB_ldsc/index.html |
| **LDL-EAS** | GLGC | 71,150 | 11,569,928 | doi.org/10.1038/s41586-021-04064-3 | http://csg.sph.umich.edu/willer/public/glgc-lipids202/.lkjhu1/ |
| **height-EUR** | UKBB | 360,388 | 12,515,778 | doi.org/10.1038/s41586-018-0579-z | https://nealelab.github.io/UKBB_ldsc/index.html |
| **height-EUR** | Sibship | 71,872 | 6,101,836 | doi.org/10.1038/s41588-022-01062-7 | gwas.mrcieu.ac.uk/datasets/ieu-b-4813/ |
| **height-EAS** | Chinese | 32,921 | 3,776,576 | doi.org/10.1016/j.ajhg.2021.03.002 | doi.org/10.1016/j.ajhg.2021.03.002 |
| **height-EAS** | BBJ | 159,095 | 6,310,855 | doi.org/10.1038/s41467-019-12276-5 | http://jenger.riken.jp/en/result |
| **Lym-EUR** | UKBB | 349,856 | 12,515,778 | doi.org/10.1038/s41586-018-0579-z | https://nealelab.github.io/UKBB_ldsc/index.html |
| **Lym-EAS** | BBJ | 62,076 | 5,961,105 | doi.org/10.1038/s41588-018-0047-6 | http://jenger.riken.jp/en/result |
| **WBC-EUR** | UKBB | 350,470 | 12,515,778 | doi.org/10.1038/s41586-018-0579-z | nealelab.github.io/UKBB_ldsc/index.html |
| **WBC-EAS** | BBJ | 107,964 | 5,961,105 | doi.org/10.1038/s41588-018-0047-6 | http://jenger.riken.jp/en/result |
| **Neutro-EUR** | UKBB | 349,856 | 12,515,778 | doi.org/10.1038/s41586-018-0579-z | nealelab.github.io/UKBB_ldsc/index.html |
| **Neutro-EAS** | BBJ | 62,076 | 5,961,105 | doi.org/10.1038/s41588-018-0047-6 | http://jenger.riken.jp/en/result |
| **Mono-EUR** | UKBB | 349,856 | 12,515,778 | doi.org/10.1038/s41586-018-0579-z | nealelab.github.io/UKBB_ldsc/index.html |
| **Mono-EAS** | BBJ | 62,076 | 5,961,105 | doi.org/10.1038/s41588-018-0047-6 | http://jenger.riken.jp/en/result |
| **Eosino-EUR** | UKBB | 349,856 | 12,515,778 | doi.org/10.1038/s41586-018-0579-z | nealelab.github.io/UKBB_ldsc/index.html |
| **Eosino-EAS** | BBJ | 62,076 | 5,961,105 | doi.org/10.1038/s41588-018-0047-6 | http://jenger.riken.jp/en/result |
| **Baso-EUR** | UKBB | 349,856 | 12,515,778 | doi.org/10.1038/s41586-018-0579-z | nealelab.github.io/UKBB_ldsc/index.html |
| **Baso-EAS** | BBJ | 62,076 | 5,961,105 | doi.org/10.1038/s41588-018-0047-6 | http://jenger.riken.jp/en/result |
| **Plt-EUR** | UKBB | 350,474 | 12,515,778 | doi.org/10.1038/s41586-018-0579-z | nealelab.github.io/UKBB_ldsc/index.html |
| **Plt-EAS** | BBJ | 108,208 | 5,961,105 | doi.org/10.1038/s41588-018-0047-6 | http://jenger.riken.jp/en/result |
| **RBC-EUR** | UKBB | 350,475 | 12,515,778 | doi.org/10.1038/s41586-018-0579-z | nealelab.github.io/UKBB_ldsc/index.html |
| **RBC-EAS** | BBJ | 108,794 | 5,961,105 | doi.org/10.1038/s41588-018-0047-6 | http://jenger.riken.jp/en/result |
| **MCH-EUR** | UKBB | 350,472 | 12,515,778 | doi.org/10.1038/s41586-018-0579-z | nealelab.github.io/UKBB_ldsc/index.html |
| **MCH-EAS** | BBJ | 108,054 | 5,961,105 | doi.org/10.1038/s41588-018-0047-6 | http://jenger.riken.jp/en/result |
| **MCHC-EUR** | UKBB | 350,468 | 12,515,778 | doi.org/10.1038/s41586-018-0579-z | nealelab.github.io/UKBB_ldsc/index.html |
| **MCHC-EAS** | BBJ | 108,728 | 5,961,105 | doi.org/10.1038/s41588-018-0047-6 | http://jenger.riken.jp/en/result |
| **MCV-EUR** | UKBB | 350,473 | 12,515,778 | doi.org/10.1038/s41586-018-0579-z | nealelab.github.io/UKBB_ldsc/index.html |
| **MCV-EAS** | BBJ | 108,256 | 5,961,105 | doi.org/10.1038/s41588-018-0047-6 | http://jenger.riken.jp/en/result |
| **Hb-EUR** | UKBB | 344,182 | 12,515,778 | doi.org/10.1038/s41586-018-0579-z | nealelab.github.io/UKBB_ldsc/index.html |
| **Hb-EAS** | BBJ | 108,769 | 5,961,105 | doi.org/10.1038/s41588-018-0047-6 | http://jenger.riken.jp/en/result |

**Supplementary Table 2:** GWAS sources

# 2 Supplementary Figures

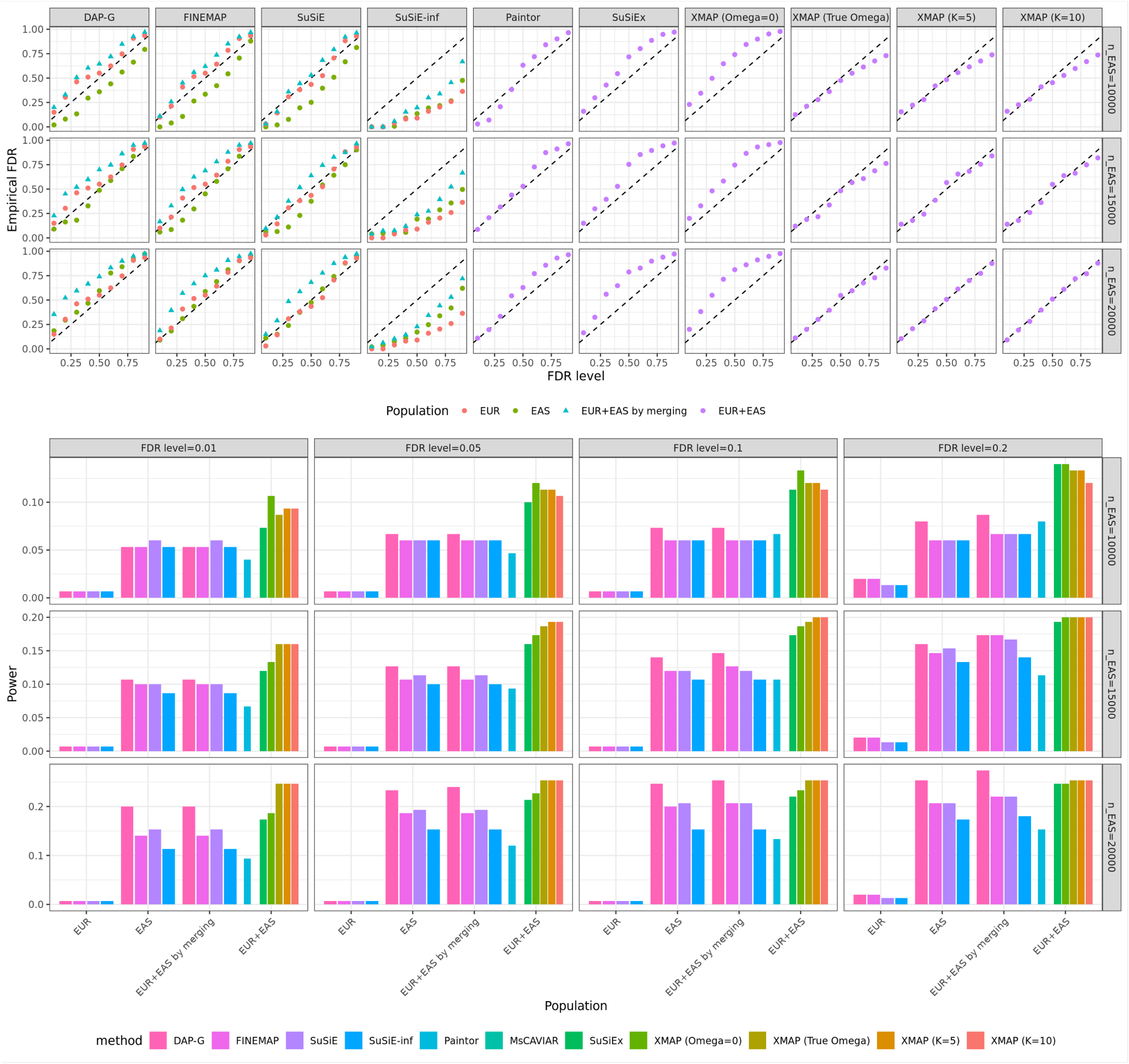


**Supplementary Figure 1:** Comparison of FDR control (top panel) and statistical power (bottom panel) among DAP-G, FINEMAP, SuSiE, SuSiE-inf Paintor, SuSiEx, and XMAP with $K_{true} = 3$, EUR sample size $n_2 = 20,000$, and EAS sample size $n_1 \in \{10,000,\ 15,000,\ 20,000\}$. Because MsCAVIAR was intractable when including more then three causal signals, it was excluded from the comparison in the setting of $K_{true} = 3$. Cyan triangles in the top panel represent the ad-hoc method by merging the PIP obtained with a single-population method across populations. Clearly, the FDR of DAP-G, SuSiEx and XMAP with $\mathbf{\Omega} = \mathbf{0}$ are **not well calibrated** here.

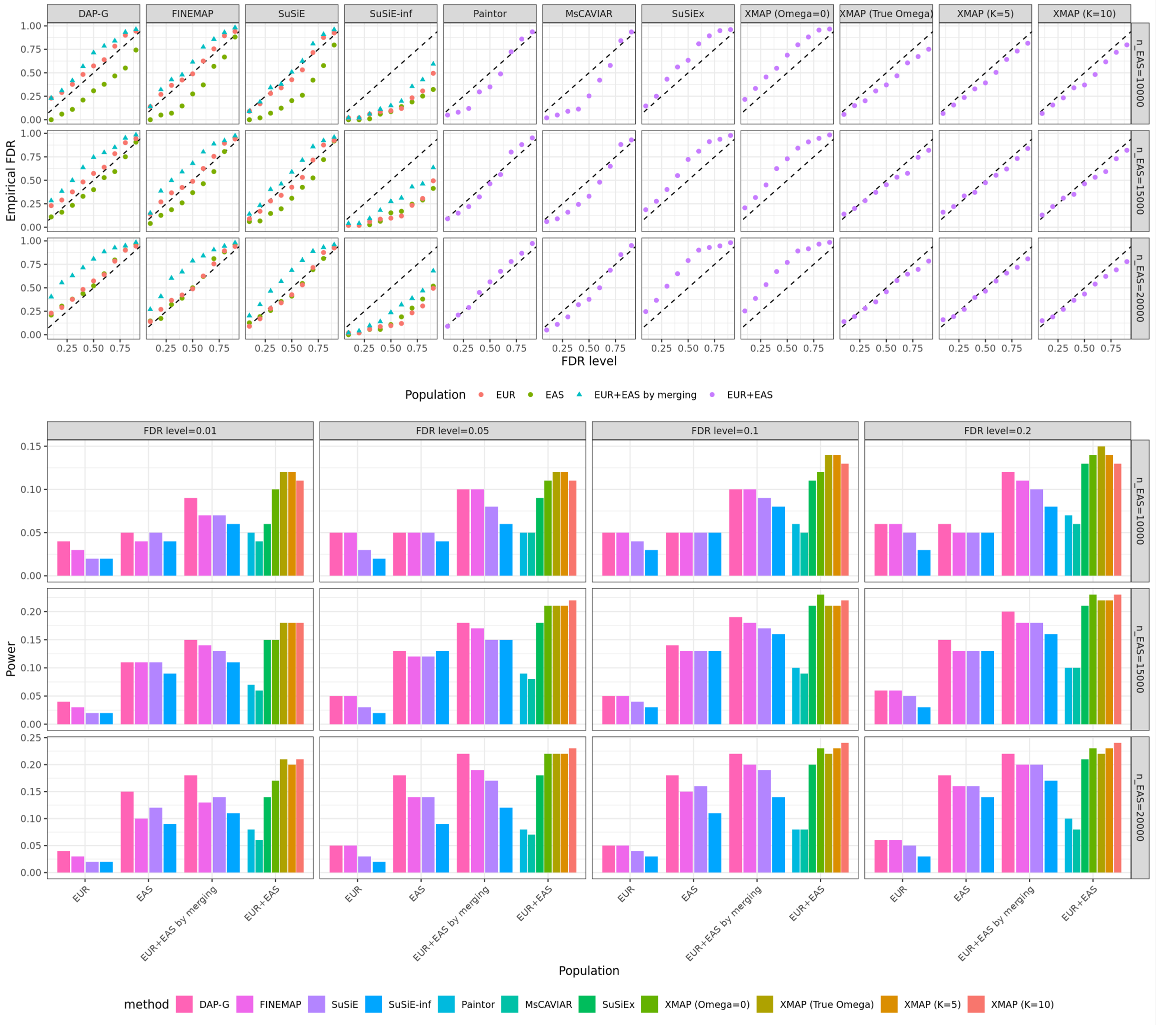


**Supplementary Figure 2:** Comparison of FDR control (top panel) and statistical power (bottom panel) among DAP-G, FINEMAP, SuSiE, SuSiE-inf Paintor, MsCAIVAR, SuSiEx, and XMAP with $K_{true} = 2$, EUR sample size $n_2 = 20,000$, and EAS sample size $n_1 \in \{10,000,\ 15,000,\ 20,000\}$. Cyan triangles in the top panel represent the ad-hoc method by merging the PIP obtained with a single-population method across populations. Clearly, the FDR of DAP-G, SuSiEx and XMAP with $\mathbf{\Omega} = \mathbf{0}$ are **not well calibrated** here.

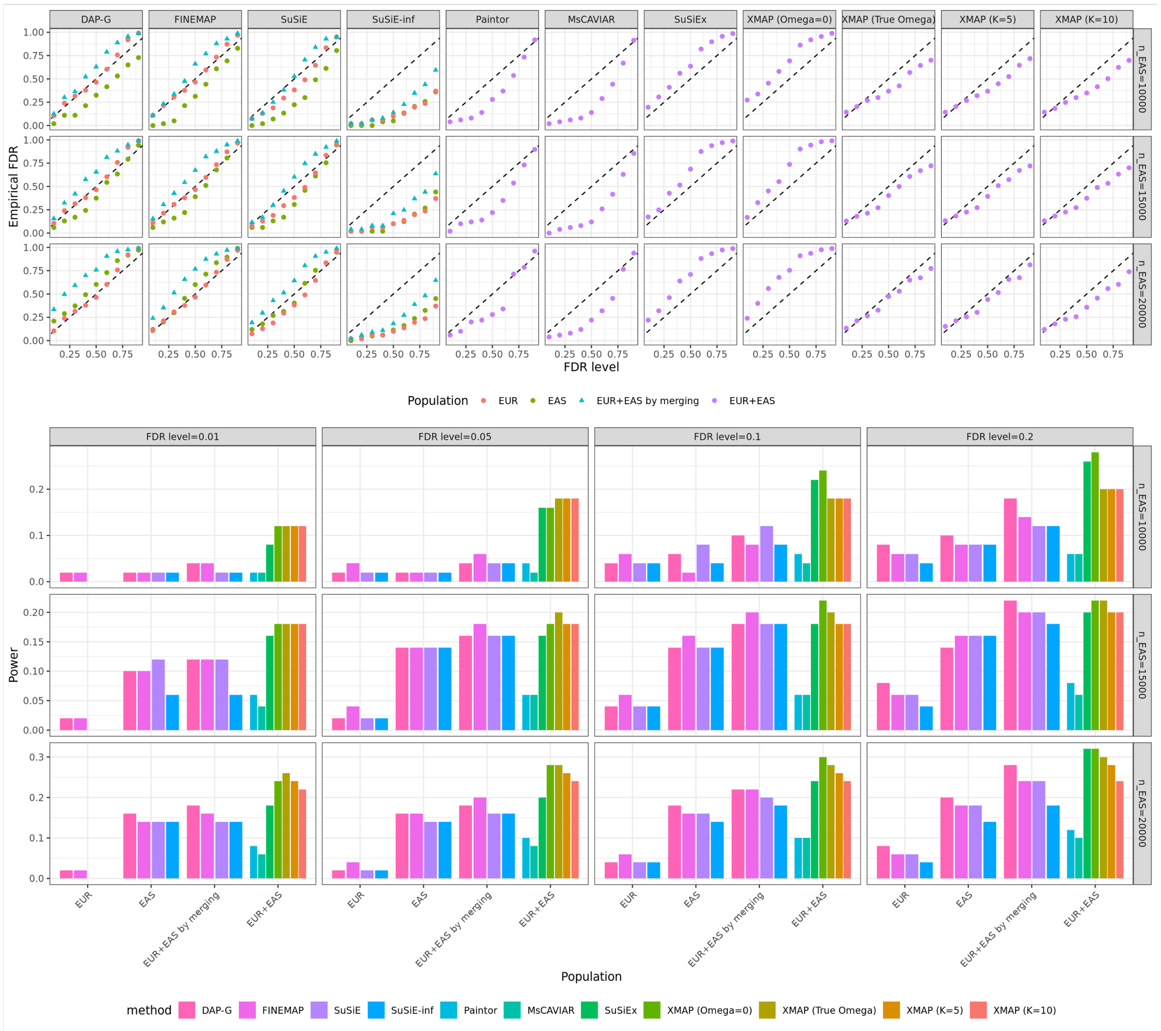


**Supplementary Figure 3:** Comparison of FDR control (top panel) and statistical power (bottom panel) among DAP-G, FINEMAP, SuSiE, SuSiE-inf Paintor, MsCAIVAR, SuSiEx, and XMAP with $K_{true} = 1$, EUR sample size $n_2 = 20,000$, and EAS sample size $n_1 \in \{10,000,\ 15,000,\ 20,000\}$. Cyan triangles in the top panel represent the ad-hoc method by merging the PIP obtained with a single-population method across populations. Clearly, the FDR of DAP-G, SuSiEx and XMAP with $\mathbf{\Omega} = \mathbf{0}$ are **not well calibrated** here.

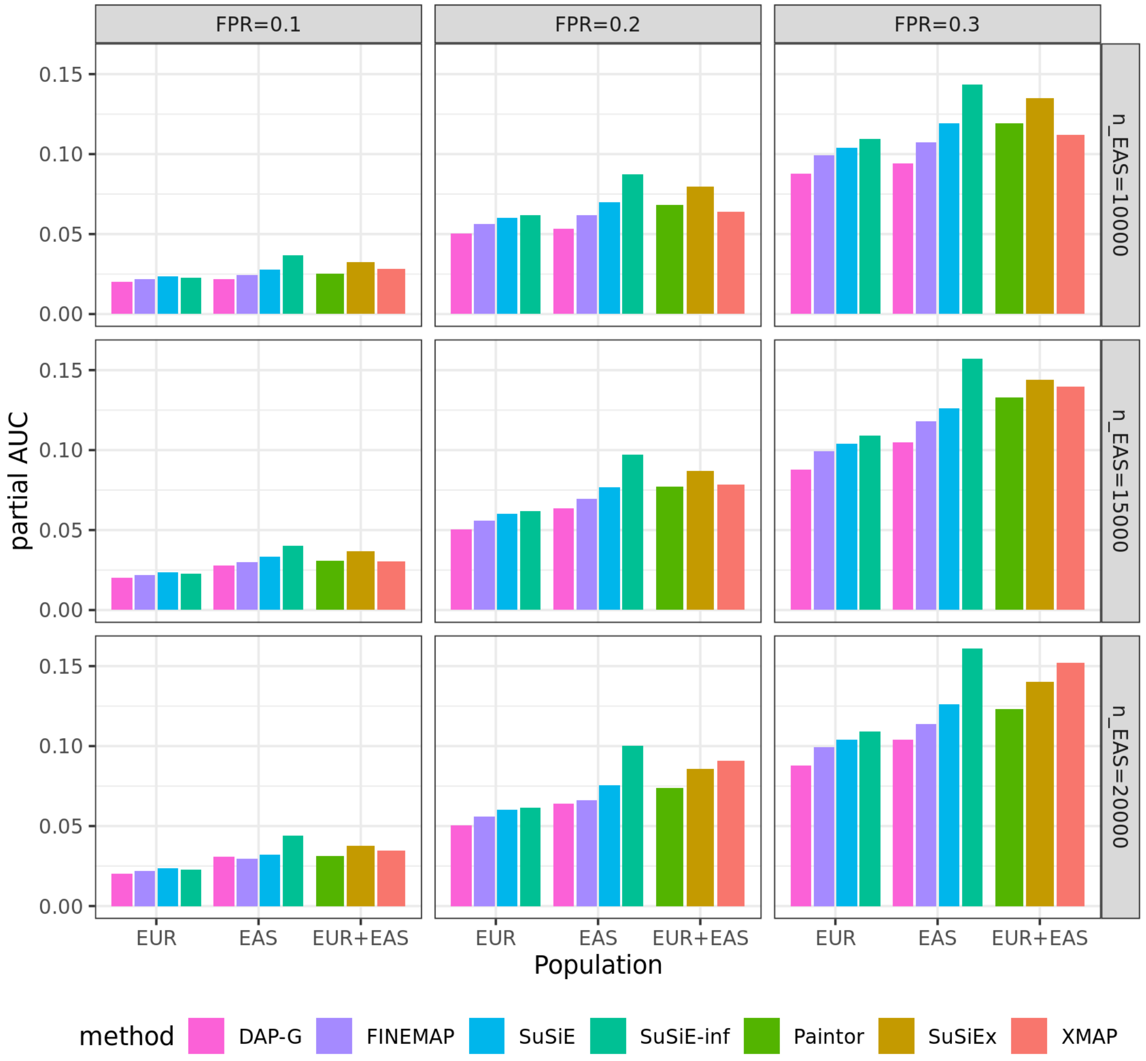


**Supplementary Figure 4:** Comparison of pAUC with different FPR thresholds among DAP-G, FINEMAP, SuSiE, SuSiE-inf, Paintor, SuSiEx, and XMAP across 50 simulations. We varied $K_{true} = 3$ and EAS sample size $n_1 \in \{10,000,\ 15,000,\ 20,000\}$, and set EUR sample size $n_2 = 20,000$. Because MsCAVIAR was intractable when including more then three causal signals, it was excluded from the comparison in the setting of $K_{true} = 3$. It is important to note that pAUC only represents the relative rankings of true causal SNPs under an empirical FPR threshold. The calibration of PIP and statistical power are more important criteria for evaluating the fine-mapping performance.

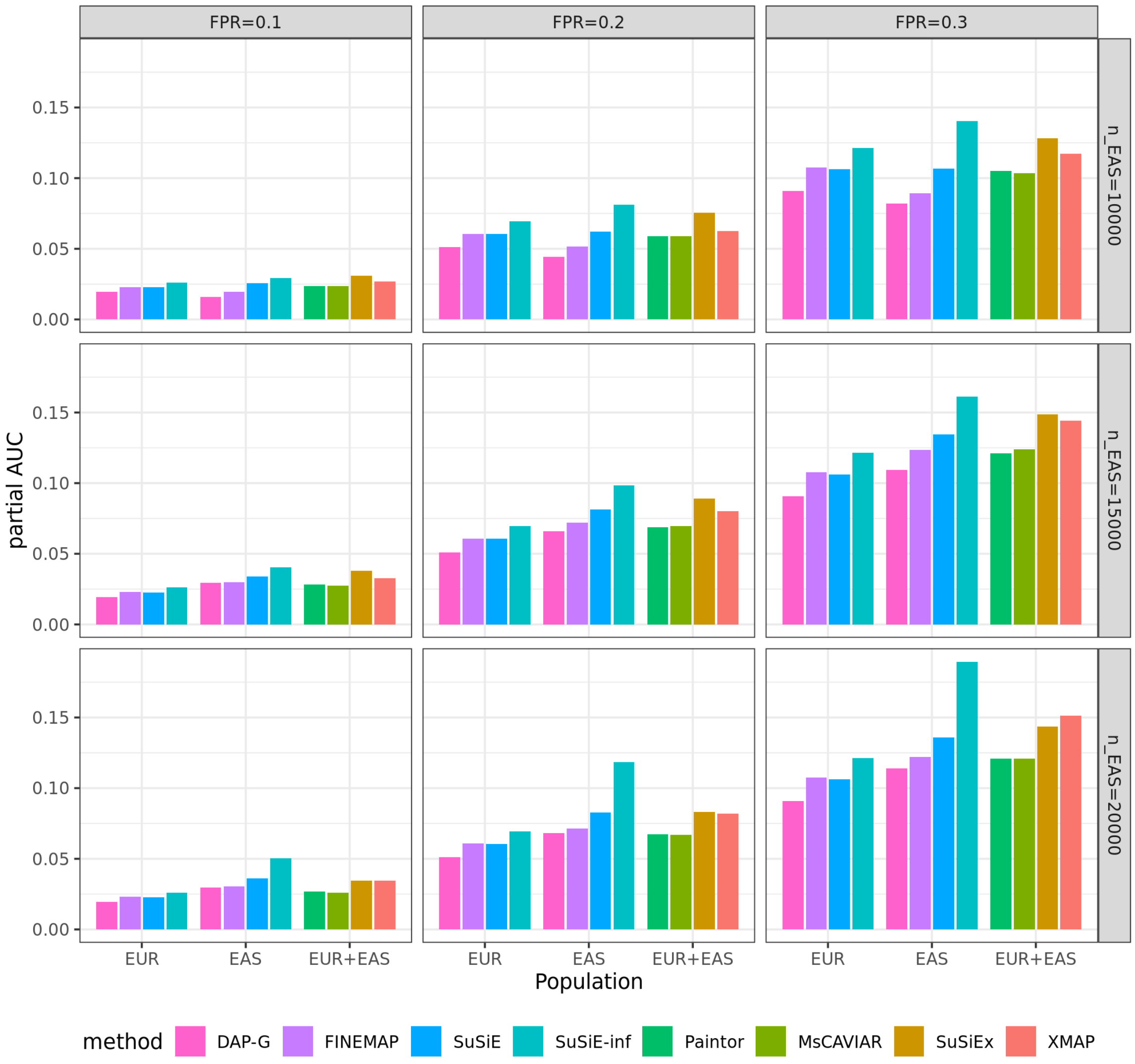


**Supplementary Figure 5:** Comparison of pAUC with different FPR thresholds among DAP-G, FINEMAP, SuSiE, SuSiE-inf, Paintor, MsCAIVAR, SuSiEx, and XMAP across 50 simulations. We varied $K_{true} = 2$ and EAS sample size $n_1 \in \{10,000,\ 15,000,\ 20,000\}$, and set EUR sample size $n_2 = 20,000$.

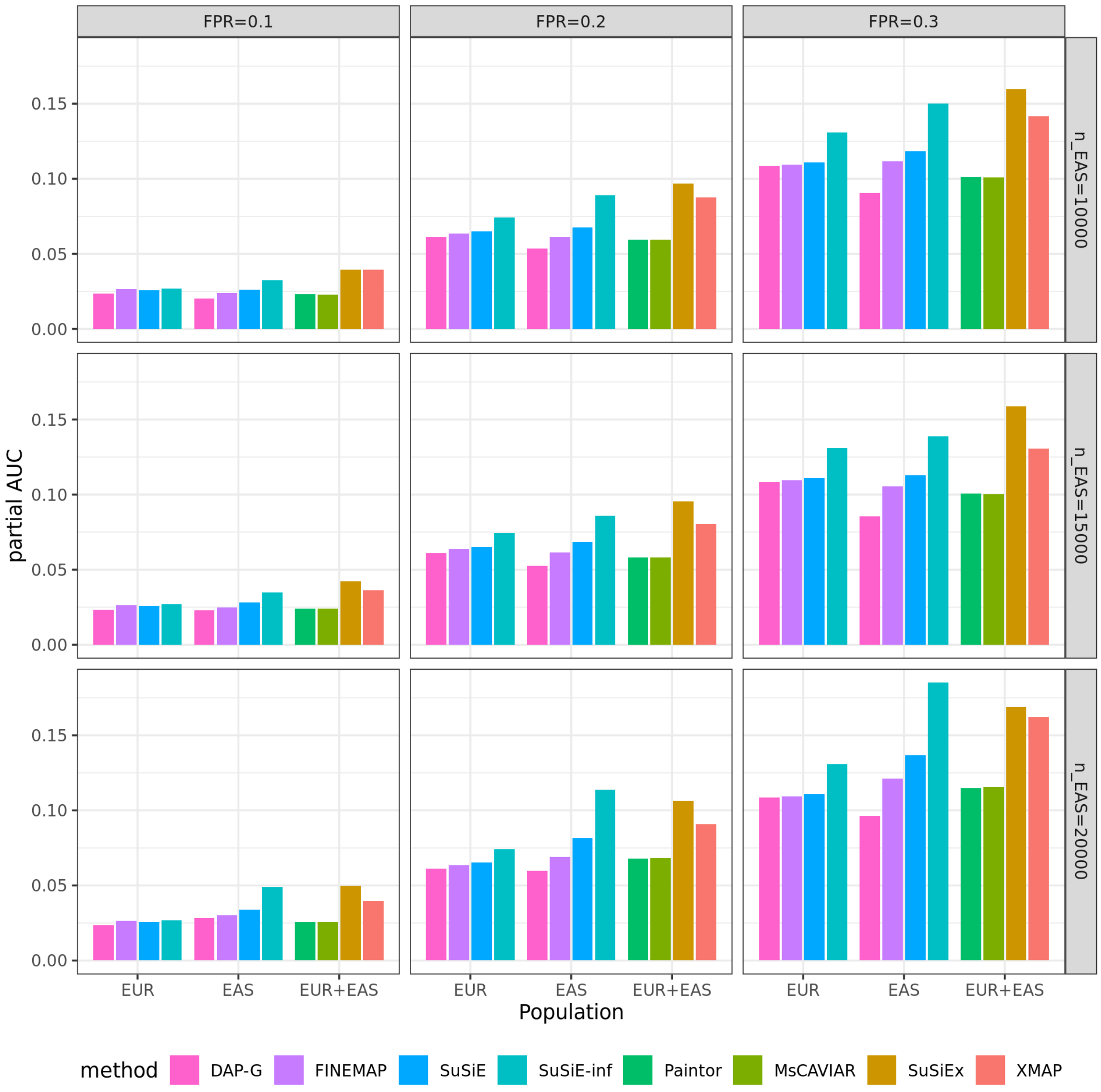


**Supplementary Figure 6:** Comparison of pAUC with different FPR thresholds among DAP-G, FINEMAP, SuSiE, SuSiE-inf, Paintor, MsCAIVAR, SuSiEx, and XMAP across 50 simulations. We varied $K_{true} = 1$ and EAS sample size $n_1 \in \{10,000,\ 15,000,\ 20,000\}$, and set EUR sample size $n_2 = 20,000$.

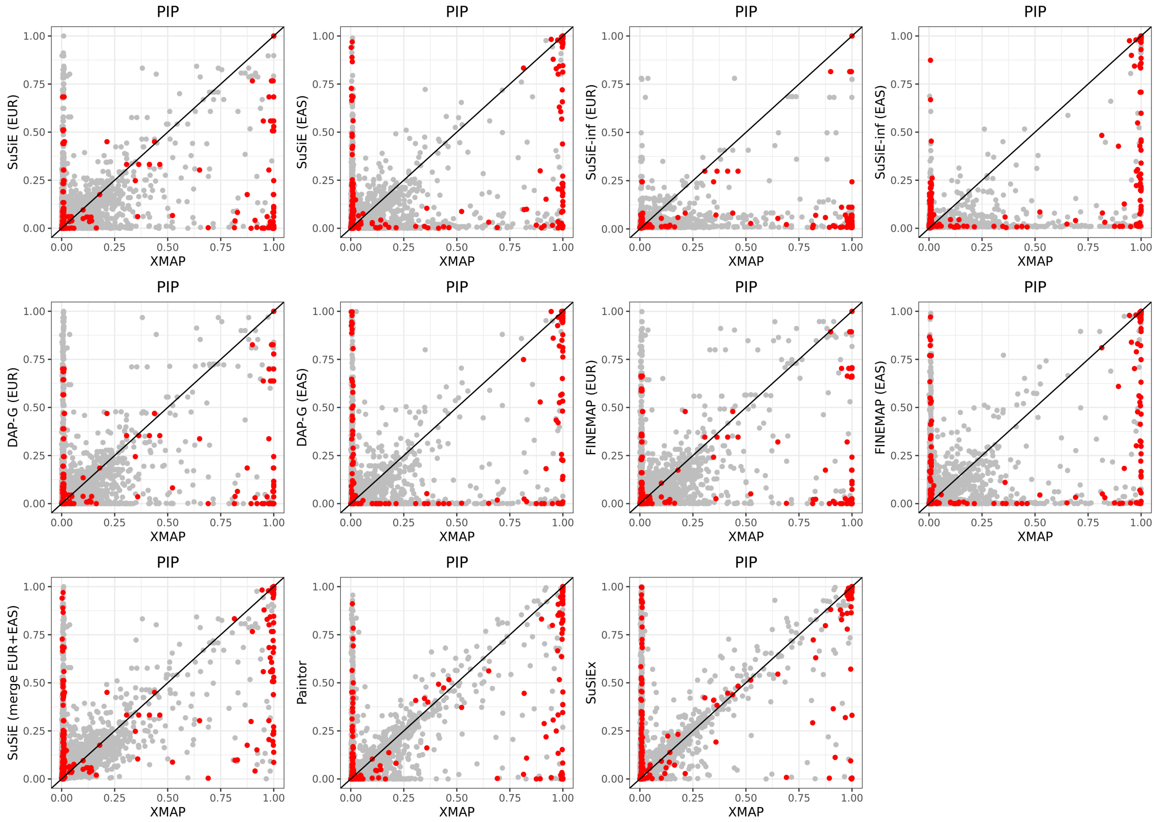


**Supplementary Figure 7:** Pairwise comparisons of PIP obtained by XMAP with those obtained by SuSiE, SuSiE-inf, DAP-G, FINEMAP, PAINTOR, MsCAVIAR, and SuSiEx when $K_{true} = 3$.

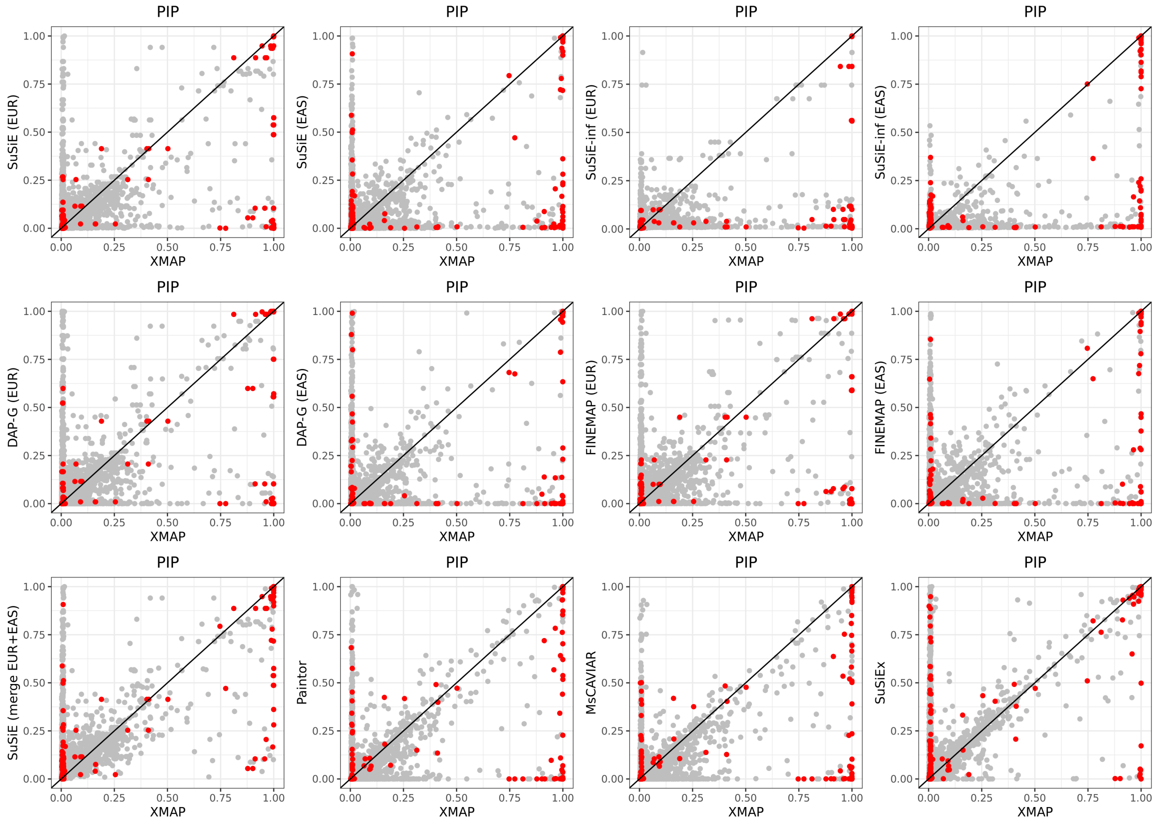


**Supplementary Figure 8:** Pairwise comparisons of PIP obtained by XMAP with those obtained by SuSiE, SuSiE-inf, DAP-G, FINEMAP, PAINTOR, MsCAVIAR, and SuSiEx when $K_{true} = 2$.

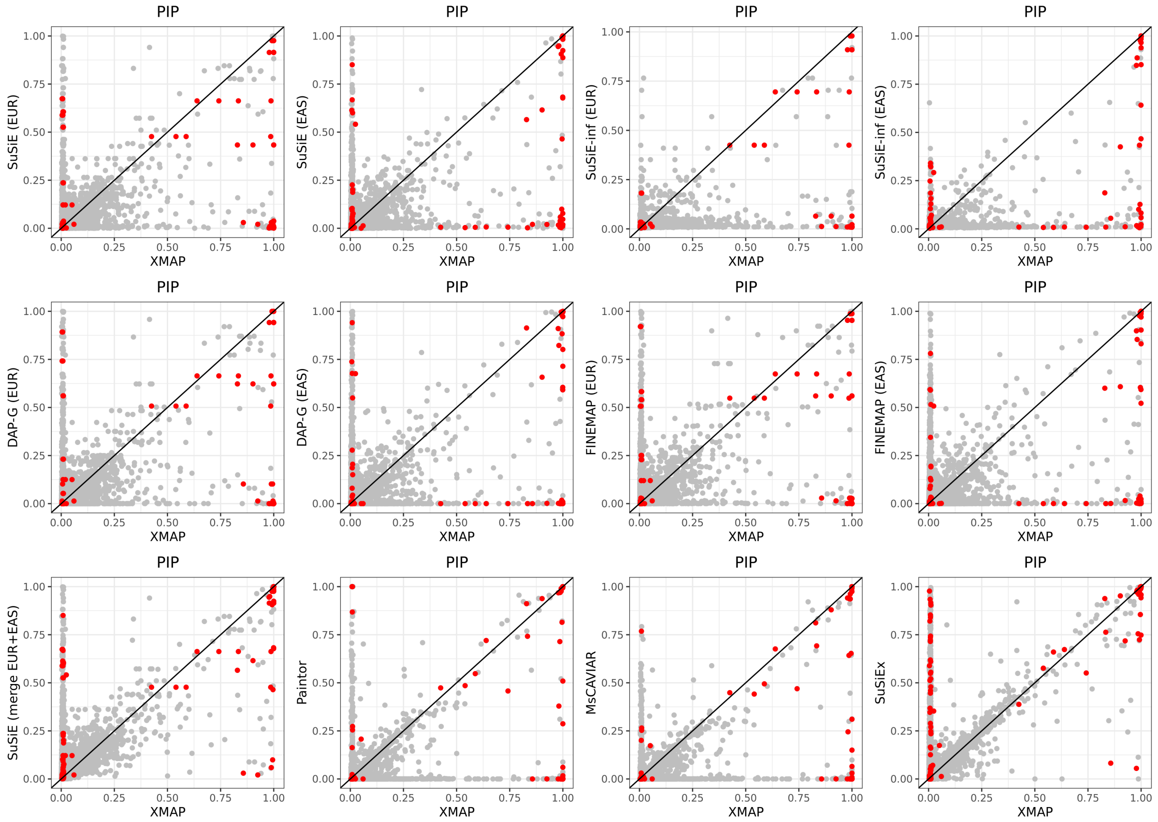


**Supplementary Figure 9:** Pairwise comparisons of PIP obtained by XMAP with those obtained by SuSiE, SuSiE-inf, DAP-G, FINEMAP, PAINTOR, MsCAVIAR, and SuSiEx when $K_{true} = 1$.

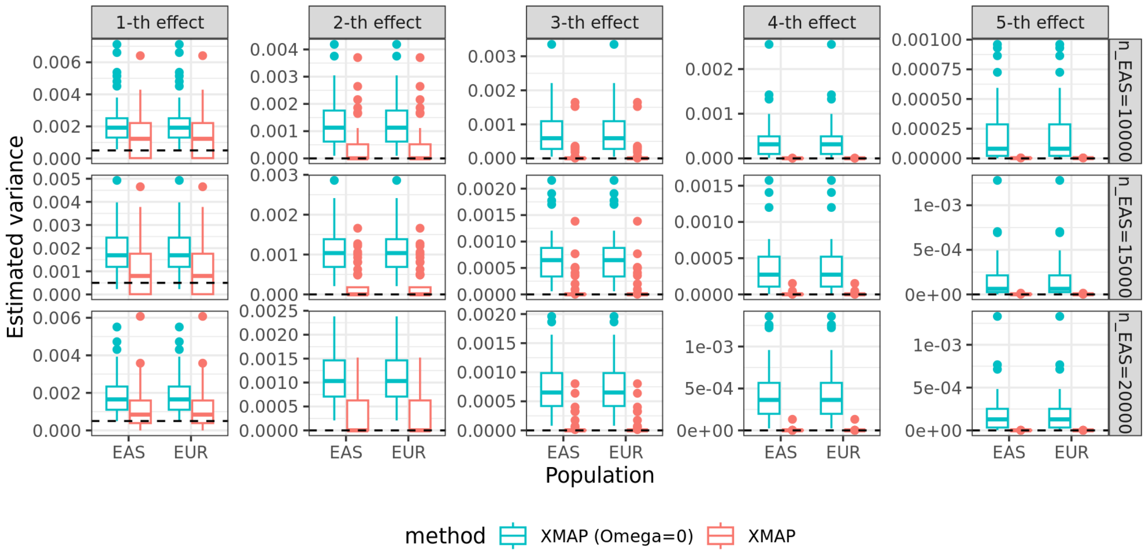


**Supplementary Figure 10:** Comparison of causal effects variance estimated by XMAP and XMAP with $\mathbf{\Omega} = \mathbf{0}$ in the presence of polygenic effects. We consider $K_{true} = 1$ here. We sort $\hat{\sigma}^2_{k1}$ and $\hat{\sigma}^2_{k1}$ for $k = 1, ..., 5$ in decreasing order, respectively. Each column represents a causal effect in the model, with decreasing estimated variance from left to right. Each row represents a setting of EAS sample size. The dashed lines represent the true variance of causal effects, which takes value of 0.25/500 for the first column and 0 for the remaining four columns.

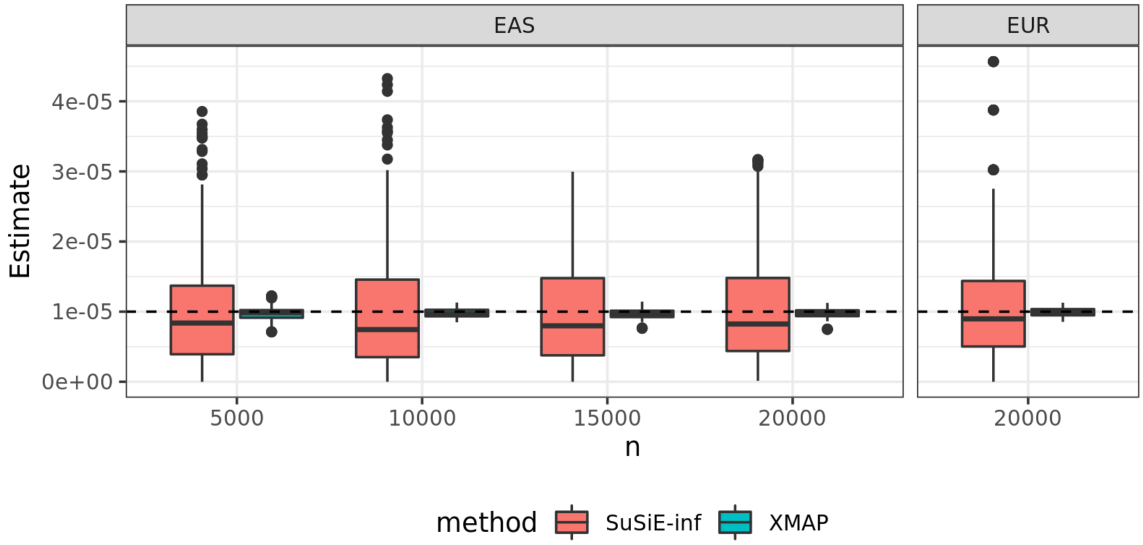


**Supplementary Figure 11:** Boxplots showing the estimated $\omega_1$ (EUR) and $\omega_2$ (EAS) obtained by XMAP and SuSiE-inf with simulation data. We varied the sample size of EAS GWAS in 5,000, 10,000, 15,000, and 20,000 and set the sample size of EUR GWAS as 20,000.

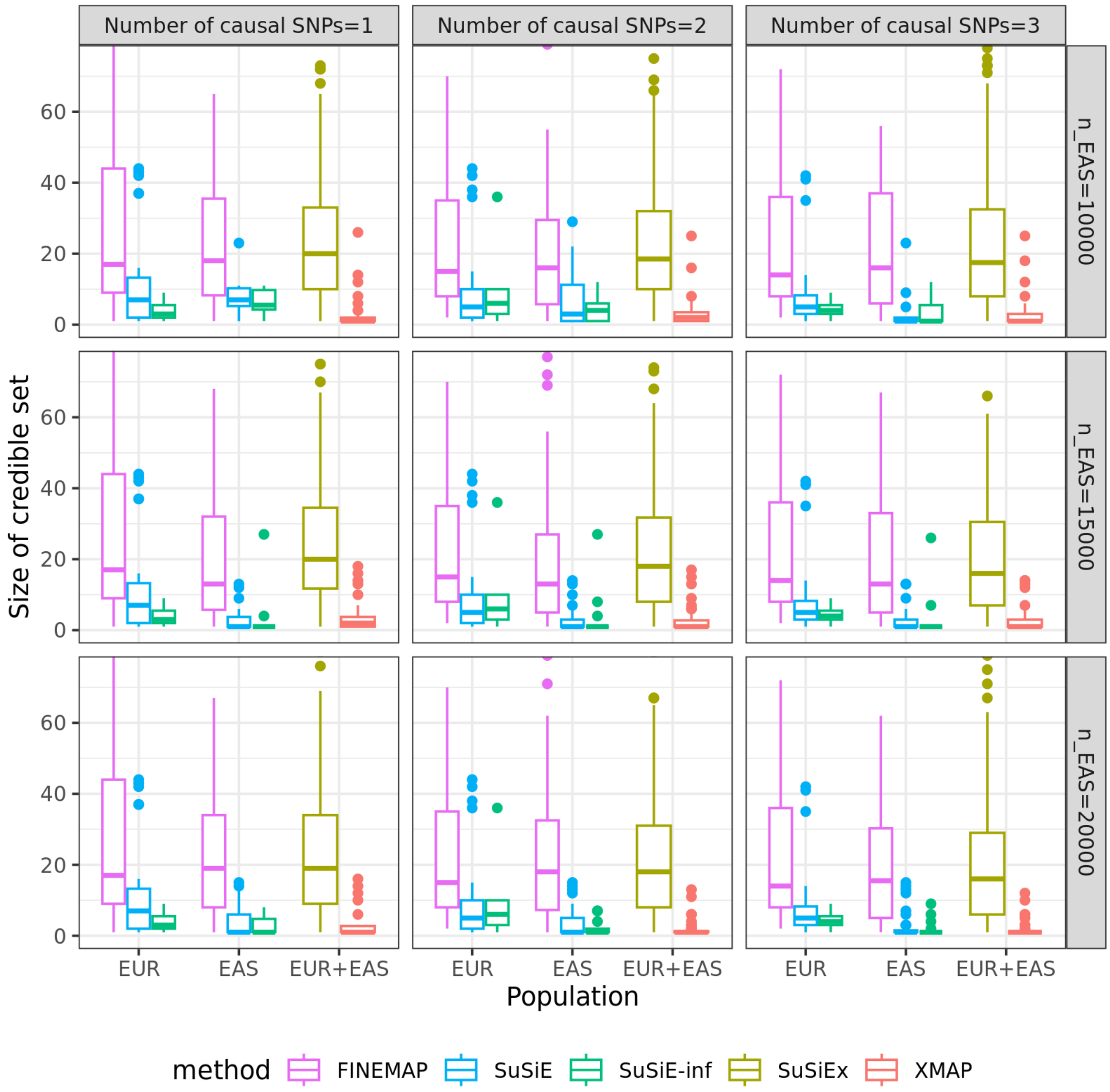


**Supplementary Figure 12:** Boxplots showing the size of level-95% credible sets generated by FINEMAP, SuSiE, SuSiE-inf, SuSiEx, and XMAP.

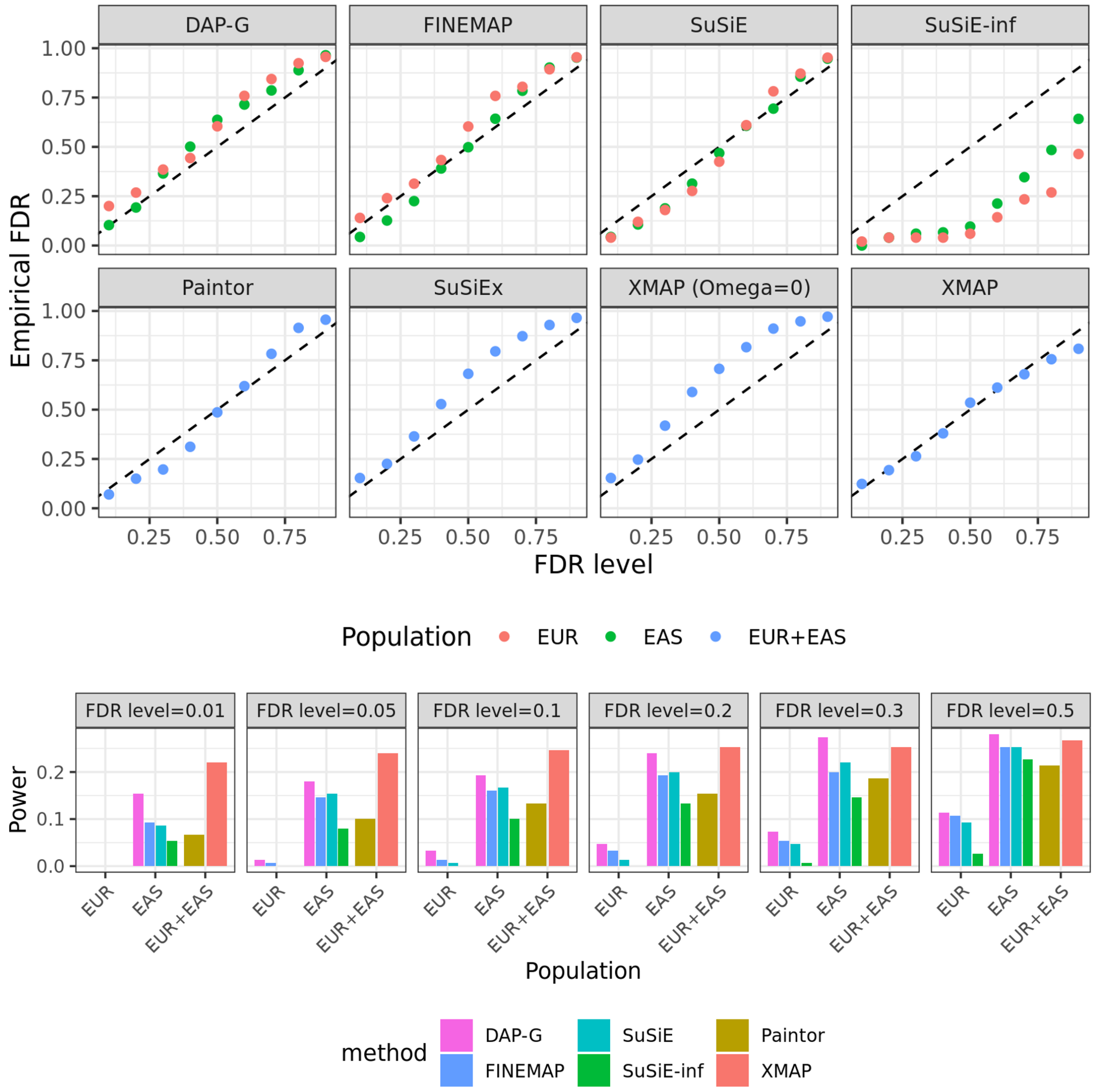


**Supplementary Figure 13:** Comparison of FDR control (top panel) and statistical power (bottom panel) when the causal effects and polygenic effects are generated from a scaled $t$-distribution with degrees of freedom $df = 16$. In the top panel, the x-axis represents the expected FDR level ($\xi$) and the y-axis represents the empirical FDR. SuSiEx is excluded in the comparison of statistical power because its FDR is not well-controlled.

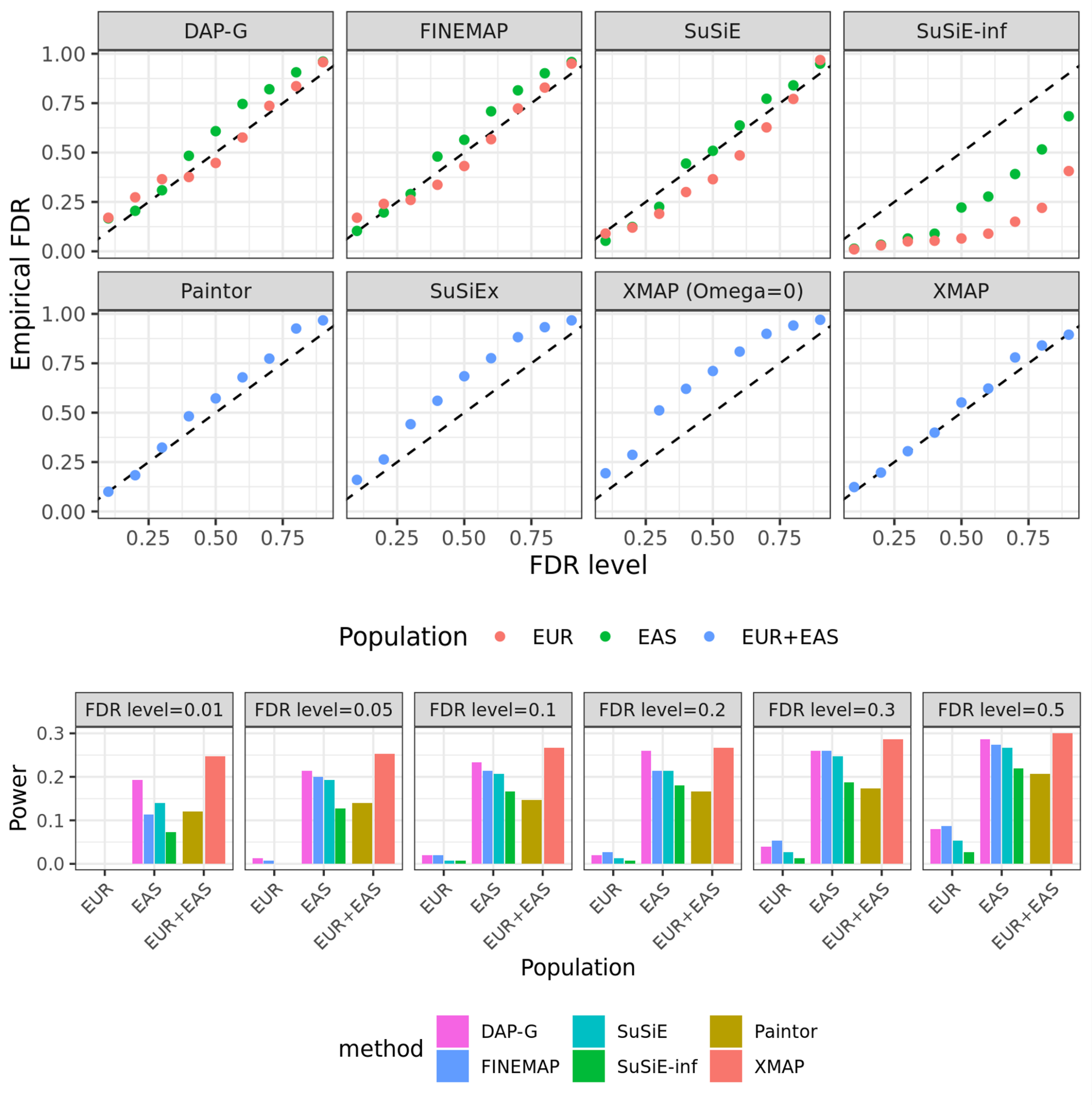


**Supplementary Figure 14:** Comparison of FDR control (top panel) and statistical power (bottom panel) when the causal effects and polygenic effects are generated from a scaled $t$-distribution with degrees of freedom $df = 4$. In the top panel, the x-axis represents the expected FDR level ($\xi$) and the y-axis represents the empirical FDR. SuSiEx is excluded in the comparison of statistical power because its FDR is not well-controlled.

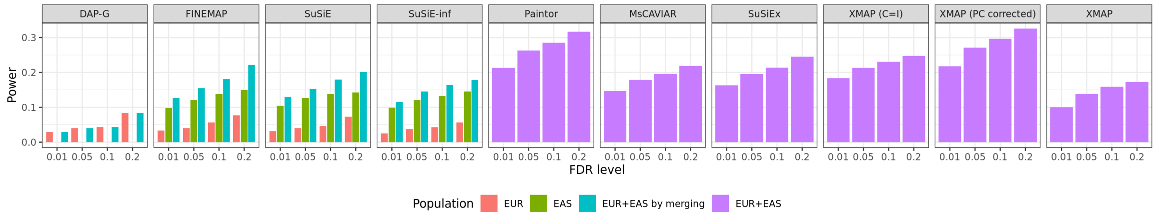


**Supplementary Figure 15:** Comparison of statistical power in the presence of confounding bias across 50 simulations. We compared the ROC among DAP-G, FINEMAP, SuSiE, SuSiE-inf, MsCAVIAR, Paintor, SuSiEx, XAMP with $\mathbf{C} = \mathbf{I}$, and XMAP. We also included the power of XMAP computed with PC-corrected GWAS data, which can be viewed as the best achievable power. It is important to note that the FDR of all methods except XMAP are severely inflated (Figure 3A in the main text).

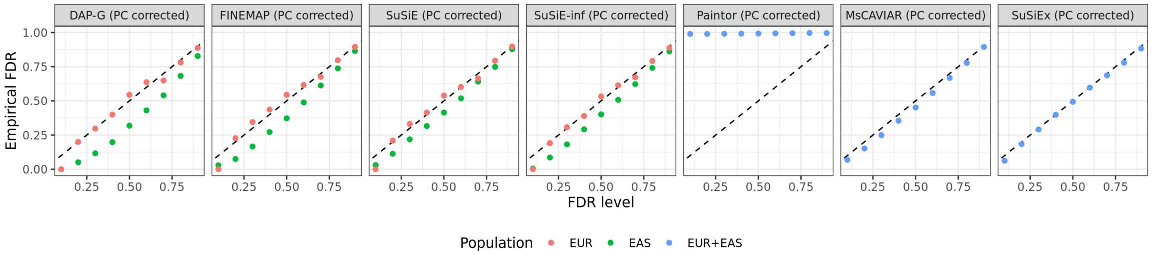


**Supplementary Figure 16:** Comparison of FDR control when the PC-corrected summary statistics was used as input for fine-mapping. We showed the FDR among DAP-G, FINEMAP, SuSiE, SuSiE-inf, MsCAVIAR, Paintor, MsCAVIAR, and SuSiEx.

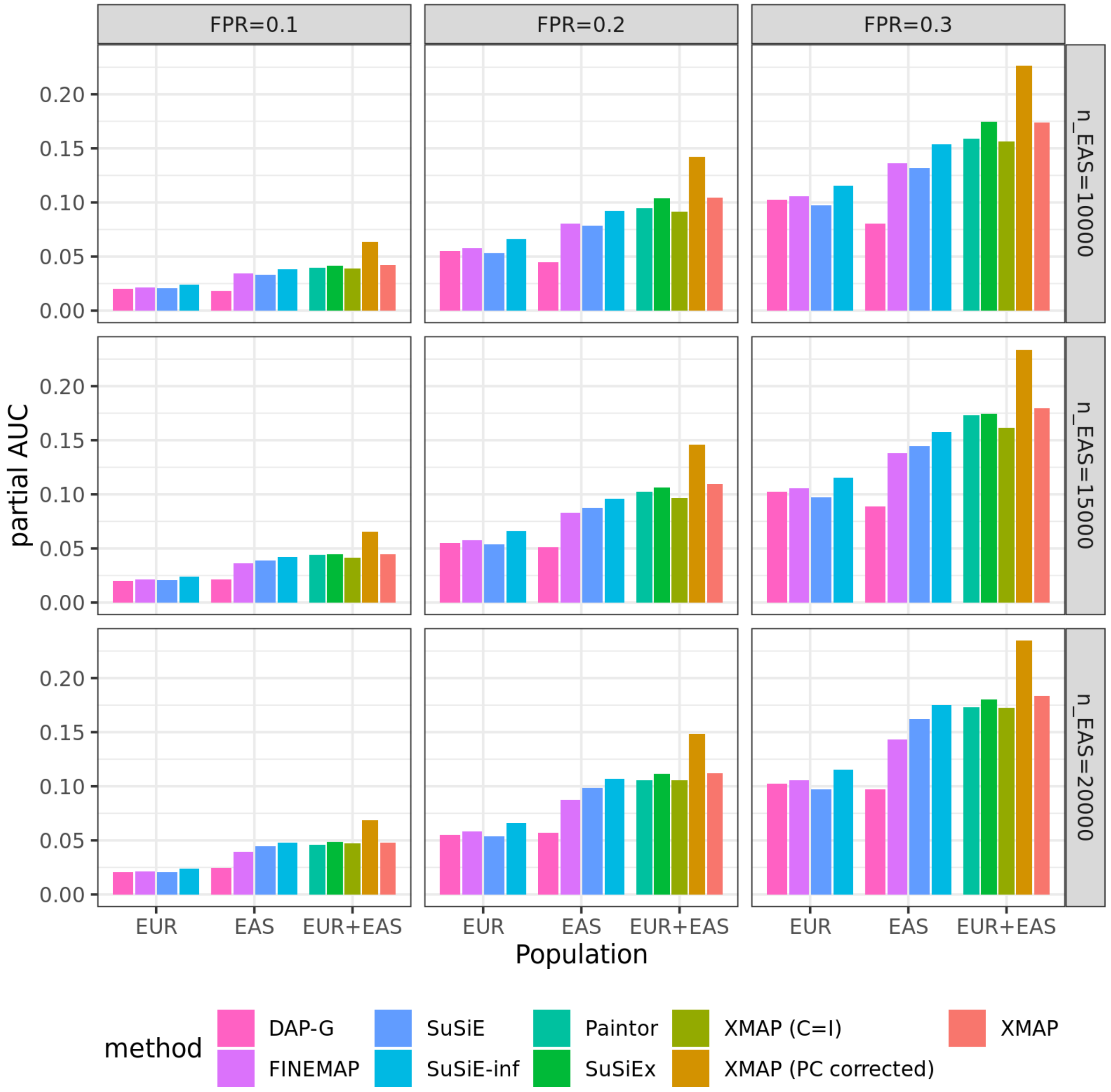


**Supplementary Figure 17:** Comparison of pAUC in the presence of confounding bias across 50 simulations. We compared the ROC among DAP-G, FINEMAP, SuSiE, SuSiE-inf, Paintor, SuSiEx, XAMP with $\mathbf{C} = \mathbf{I}$, and XMAP. We also included the ROC of XMAP computed with PC-corrected GWAS data, which can be viewed as the best achievable pAUC. We set $K_{true} = 3$ and varied EAS sample size $n_1 \in \{10,000,\ 15,000,\ 20,000\}$, and set EUR sample size $n_2 = 20,000$. Because MsCAVIAR was intractable when including more then three causal signals, it was excluded from the comparison in the setting of $K_{true} = 3$. It is important to note that the FDR of all methods except XMAP are severely inflated (Figure 3A in the main text).

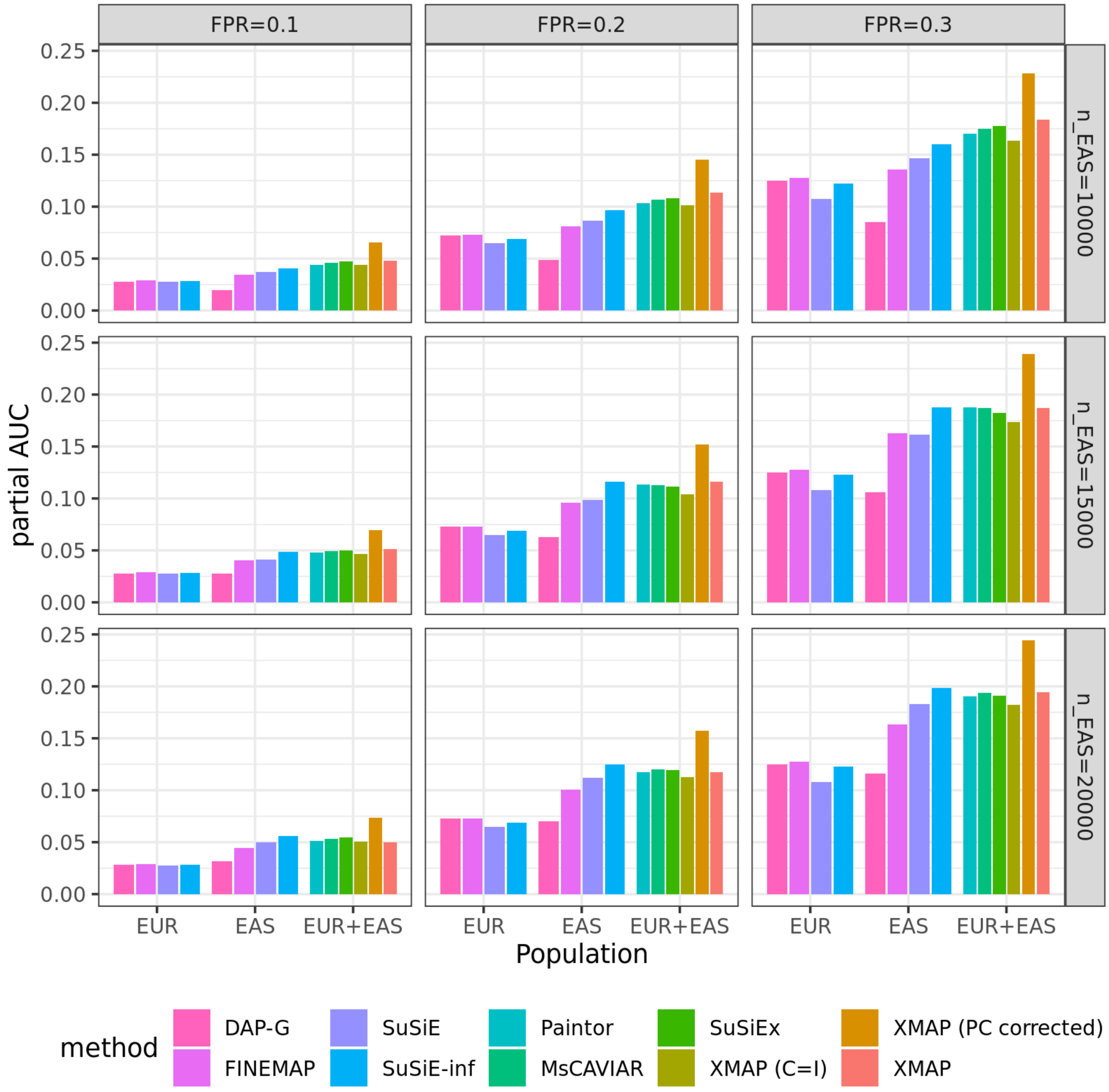


**Supplementary Figure 18:** Comparison of pAUC in the presence of confounding bias across 50 simulations. We compared the ROC among DAP-G, FINEMAP, SuSiE, SuSiE-inf, MsCAVIAR, Paintor, SuSiEx, XAMP with $\mathbf{C} = \mathbf{I}$, and XMAP. We also included the ROC of XMAP computed with PC-corrected GWAS data, which can be viewed as the best achievable pAUC. We set $K_{true} = 2$ and varied EAS sample size $n_1 \in \{10,000,\ 15,000,\ 20,000\}$, and set EUR sample size $n_2 = 20,000$. It is important to note that the FDR of all methods except XMAP are severely inflated (Figure 3A in the main text).

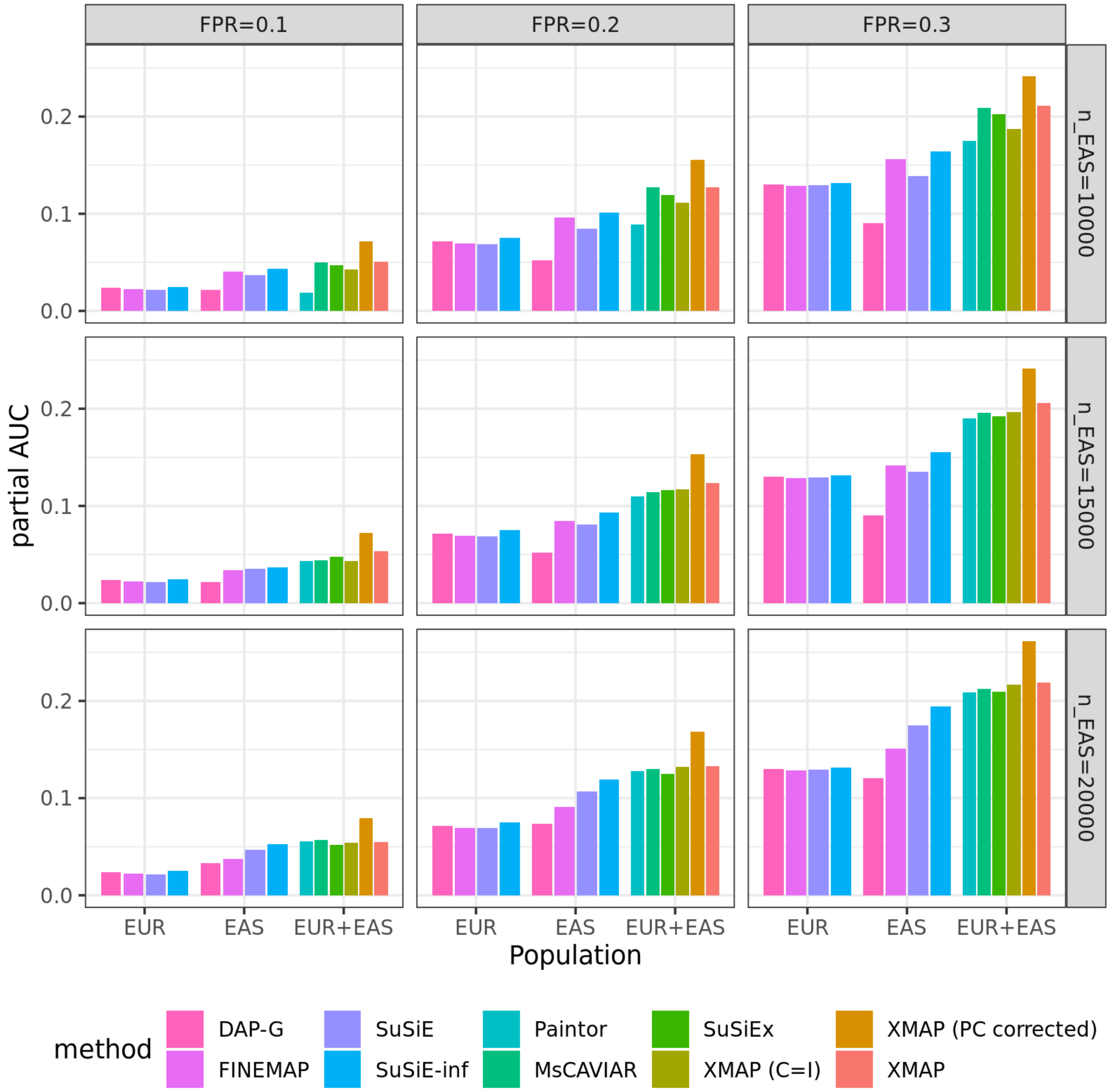


**Supplementary Figure 19:** Comparison of pAUC in the presence of confounding bias across 50 simulations. We compared the ROC among DAP-G, FINEMAP, SuSiE, SuSiE-inf, MsCAVIAR, Paintor, SuSiEx, XAMP with $\mathbf{C} = \mathbf{I}$, and XMAP. We also included the ROC of XMAP computed with PC-corrected GWAS data, which can be viewed as the best achievable pAUC. We set $K_{true} = 1$ and varied EAS sample size $n_1 \in \{10,000,\ 15,000,\ 20,000\}$, and set EUR sample size $n_2 = 20,000$. It is important to note that the FDR of all methods except XMAP are severely inflated (Figure 3A in the main text).

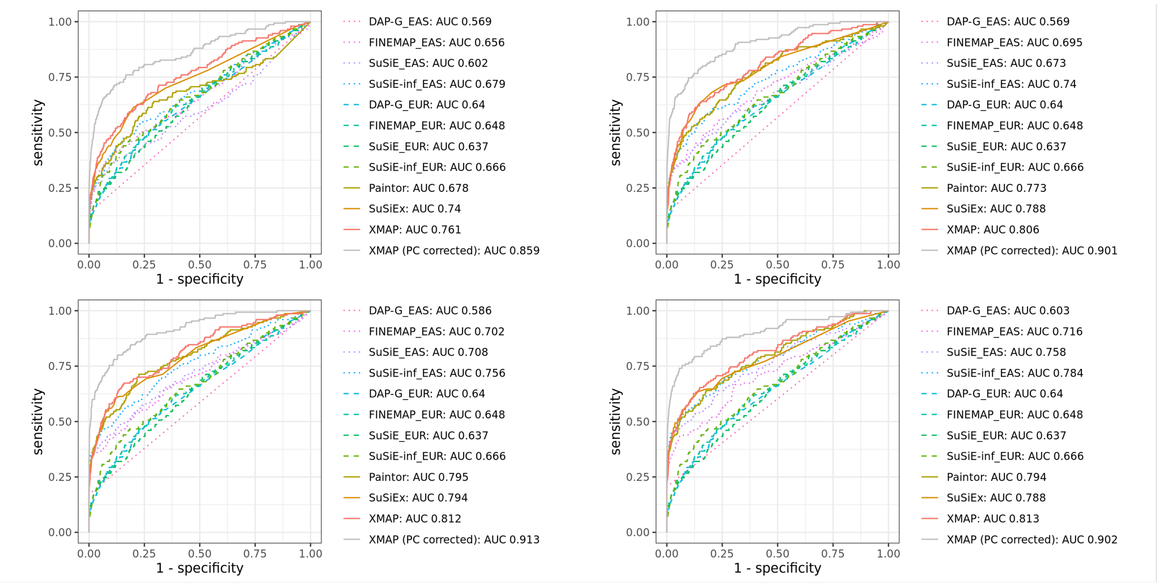


**Supplementary Figure 20:** Comparison of ROC in the presence of confounding bias across 50 simulations. We compared the ROC among DAP-G, FINEMAP, SuSiE, SuSiE-inf, Paintor, SuSiEx, and XMAP. We also included the ROC of XMAP computed with PC-corrected GWAS data, which can be viewed as the best achievable ROC. We set $K_{true} = 3$ and considered different settings of $n_1$: 5,000 (top left), 10,000 (top right), 15,000 (bottom left), and 20,000 (bottom right). Because MsCAVIAR was intractable when including more then three causal signals, it was excluded from the comparison in the setting of $K_{true} = 3$.

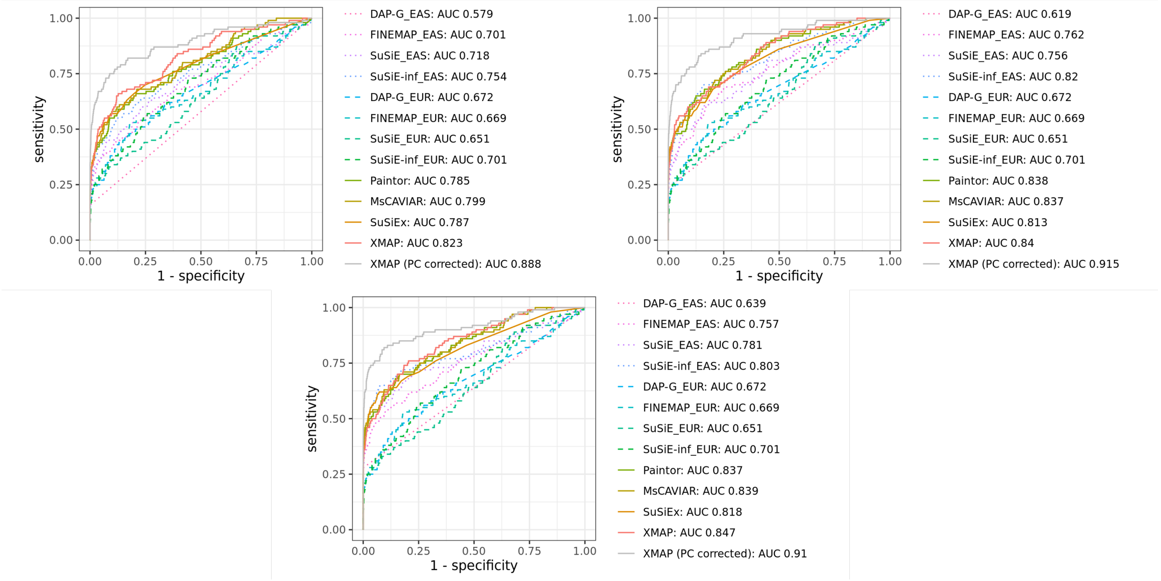


**Supplementary Figure 21:** Comparison of ROC in the presence of confounding bias across 50 simulations. We compared the ROC among DAP-G, FINEMAP, SuSiE, SuSiE-inf, MsCAVIAR, Paintor, SuSiEx, and XMAP. We also included the ROC of XMAP computed with PC-corrected GWAS data, which can be viewed as the best achievable ROC. We set $K_{true} = 2$ and set $K_{true} = 2$. We considered different settings of $n_1$: 5,000 (Figure 3 C in the main text), 10,000 (top left), 15,000 (top right), and 20,000 (bottom).

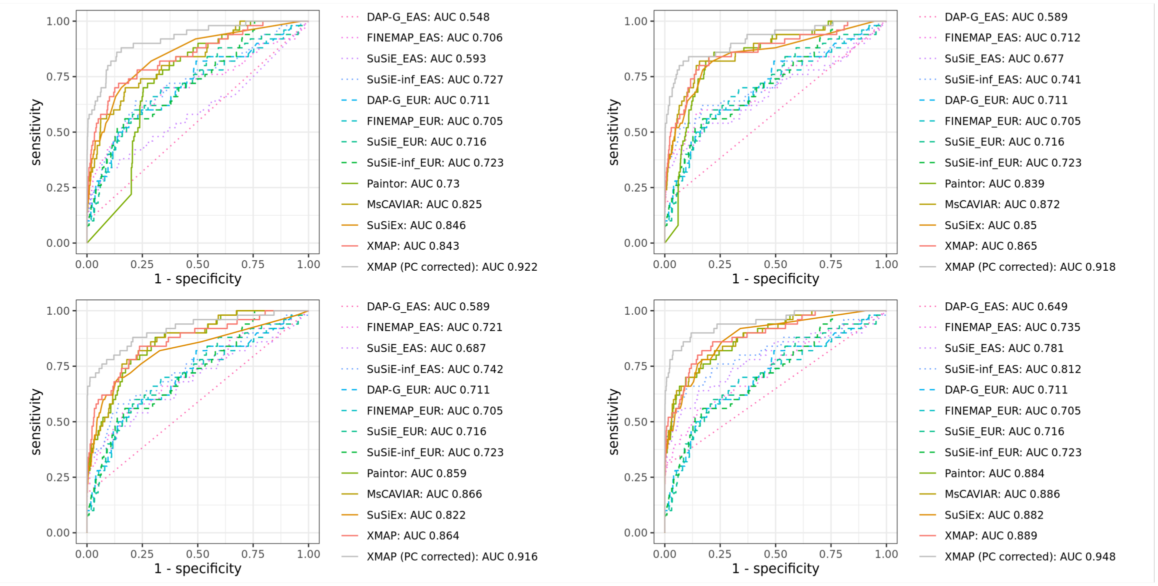


**Supplementary Figure 22:** Comparison of ROC in the presence of confounding bias across 50 simulations. We compared the ROC among DAP-G, FINEMAP, SuSiE, SuSiE-inf, MsCAVIAR, Paintor, SuSiEx, and XMAP. We also included the ROC of XMAP computed with PC-corrected GWAS data, which can be viewed as the best achievable ROC. We set $K_{true} = 1$ and considered different settings of $n_1$: 5,000 (top left), 10,000 (top right), 15,000 (bottom left), and 20,000 (bottom right).

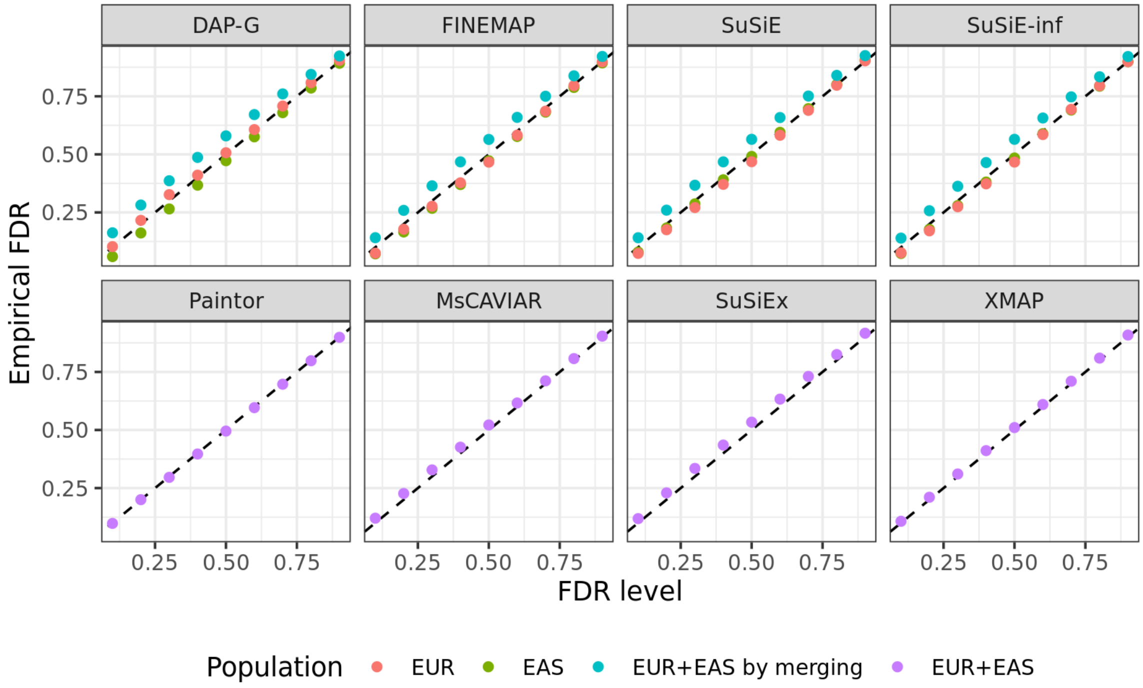


**Supplementary Figure 23:** Comparison of FDR control among DAP-G, FINEMAP, SuSiE, SuSiE-inf, Paintor, MsCAIVAR, SuSiEx, and XMAP in the setting without polygenic effects. Each of the $K_{true}$ causal SNPs explains 1% phenotypic variance.

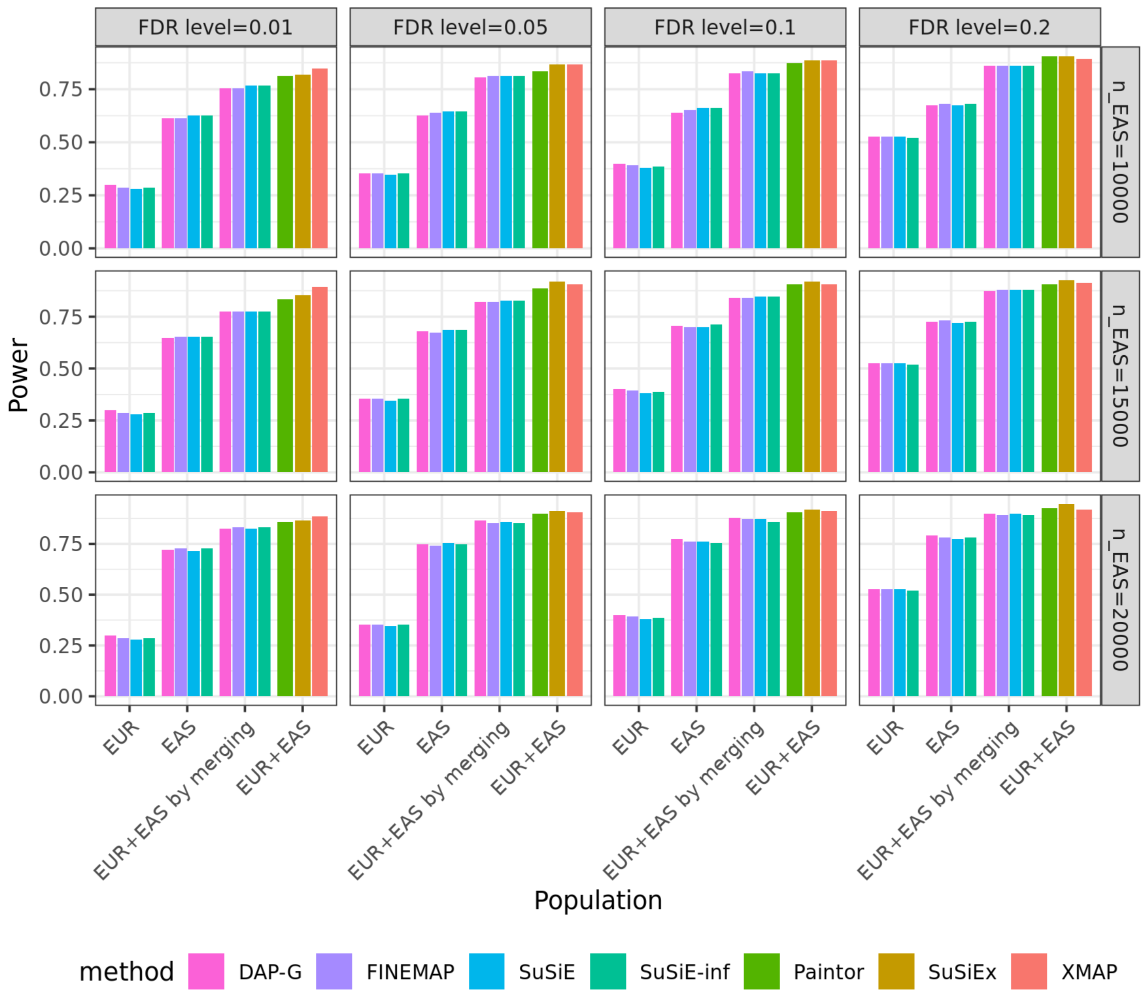


**Supplementary Figure 24:** Comparison of statistical power among DAP-G, FINEMAP, SuSiE, SuSiE-inf, Paintor, MsCAIVAR, SuSiEx, and XMAP in the setting without polygenic effects. We set $K_{true} = 3$, EUR sample size $n_2 = 20,000$, and varied EAS sample size $n_1 \in \{10,000,\ 15,000,\ 20,000\}$. Each of the $K_{true}$ causal SNPs explains 1% phenotypic variance. Because MsCAVIAR was intractable when including more then three causal signals, it was excluded from the comparison in the setting of $K_{true} = 3$.

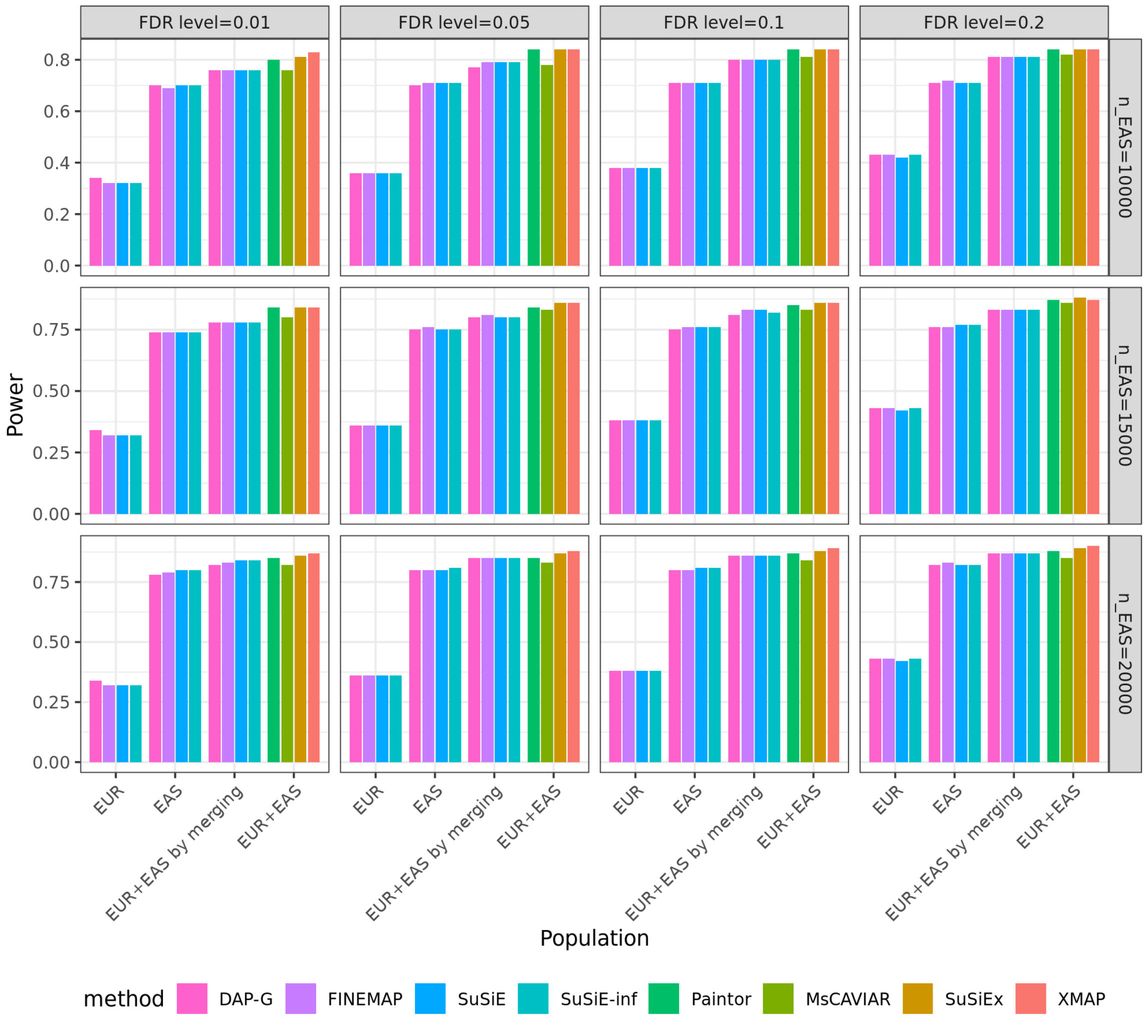


**Supplementary Figure 25:** Comparison of statistical power among DAP-G, FINEMAP, SuSiE, SuSiE-inf, Paintor, MsCAIVAR, SuSiEx, and XMAP in the setting without polygenic effects. We set $K_{true} = 2$, EUR sample size $n_2 = 20,000$, and varied EAS sample size $n_1 \in \{10,000,\ 15,000,\ 20,000\}$. Each of the $K_{true}$ causal SNPs explains 1% phenotypic variance.

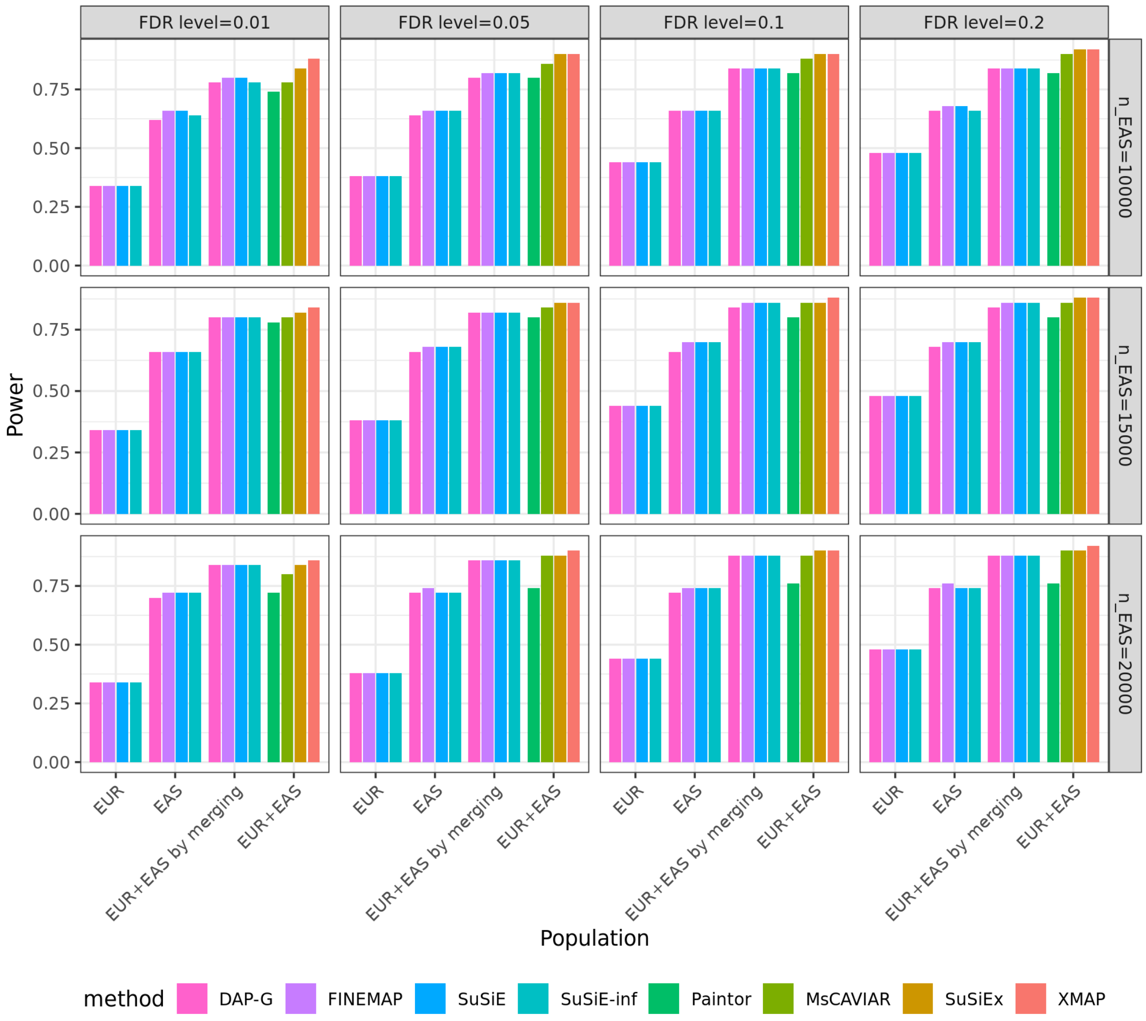


**Supplementary Figure 26:** Comparison of statistical power among DAP-G, FINEMAP, SuSiE, SuSiE-inf, Paintor, MsCAIVAR, SuSiEx, and XMAP in the setting without polygenic effects. We set $K_{true} = 1$, EUR sample size $n_2 = 20,000$, and varied EAS sample size $n_1 \in \{10,000,\ 15,000,\ 20,000\}$. Each of the $K_{true}$ causal SNPs explains 1% phenotypic variance.

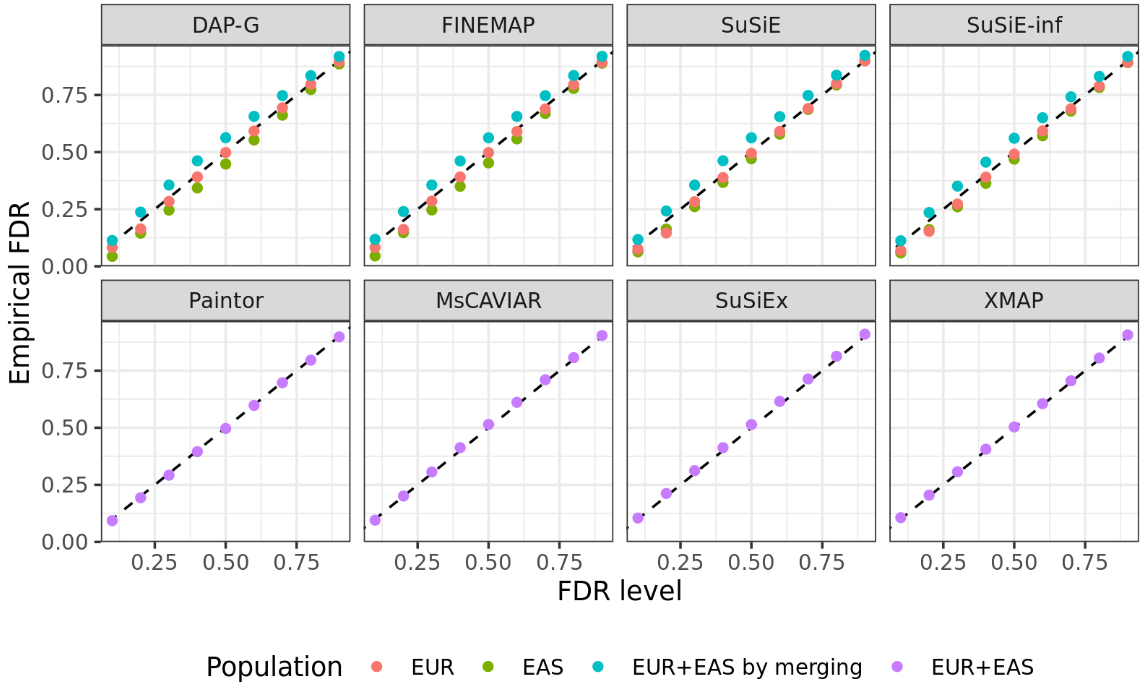


**Supplementary Figure 27:** Comparison of FDR control among DAP-G, FINEMAP, SuSiE, SuSiE-inf, Paintor, MsCAIVAR, SuSiEx, and XMAP in the setting without polygenic effects. Each of the $K_{true}$ causal SNPs explains 1% phenotypic variance.

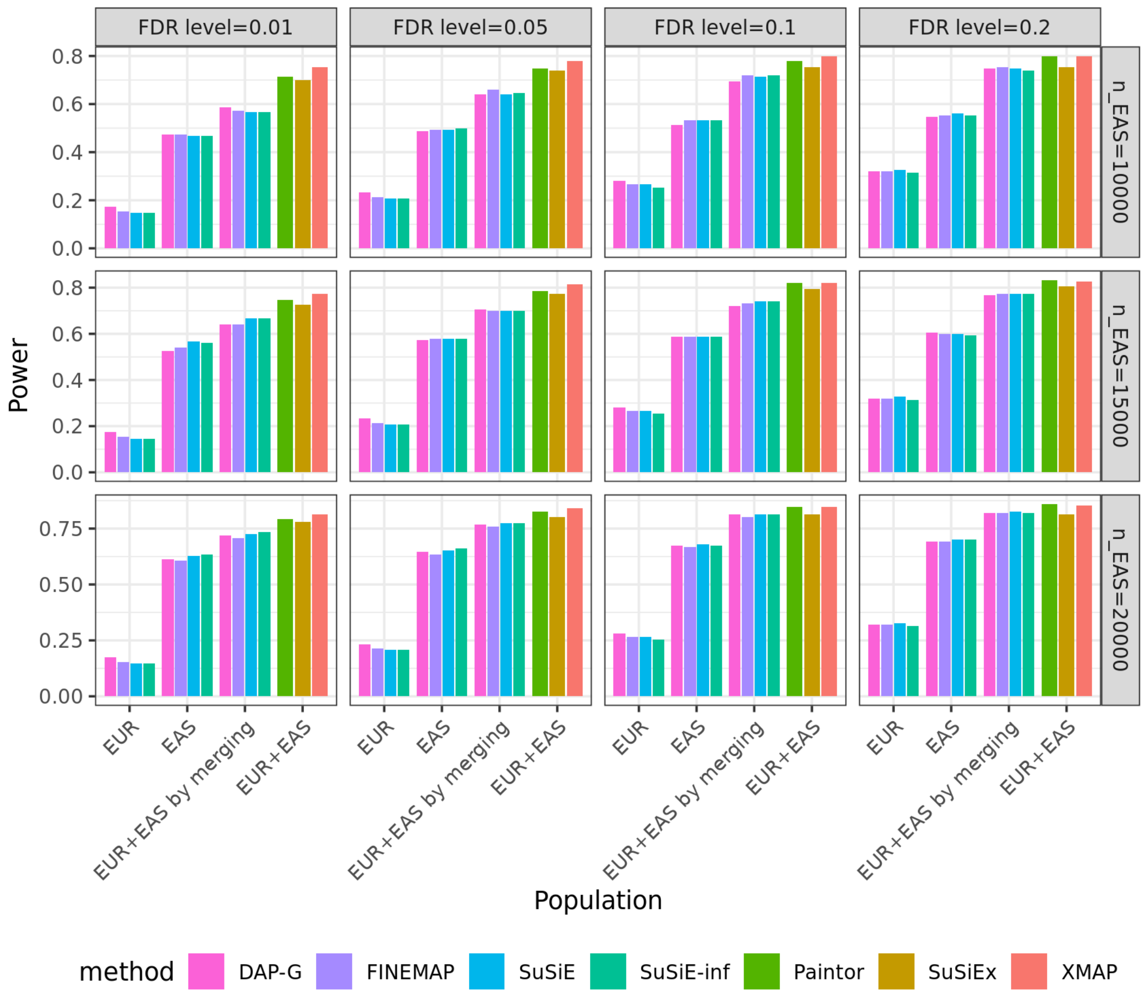


**Supplementary Figure 28:** Comparison of statistical power among DAP-G, FINEMAP, SuSiE, SuSiE-inf, Paintor, MsCAIVAR, SuSiEx, and XMAP in the setting without polygenic effects. We set $K_{true} = 3$, EUR sample size $n_2 = 20,000$, and varied EAS sample size $n_1 \in \{10,000,\ 15,000,\ 20,000\}$. Each of the $K_{true}$ causal SNPs explains 0.5% phenotypic variance. Because MsCAVIAR was intractable when including more then three causal signals, it was excluded from the comparison in the setting of $K_{true} = 3$.

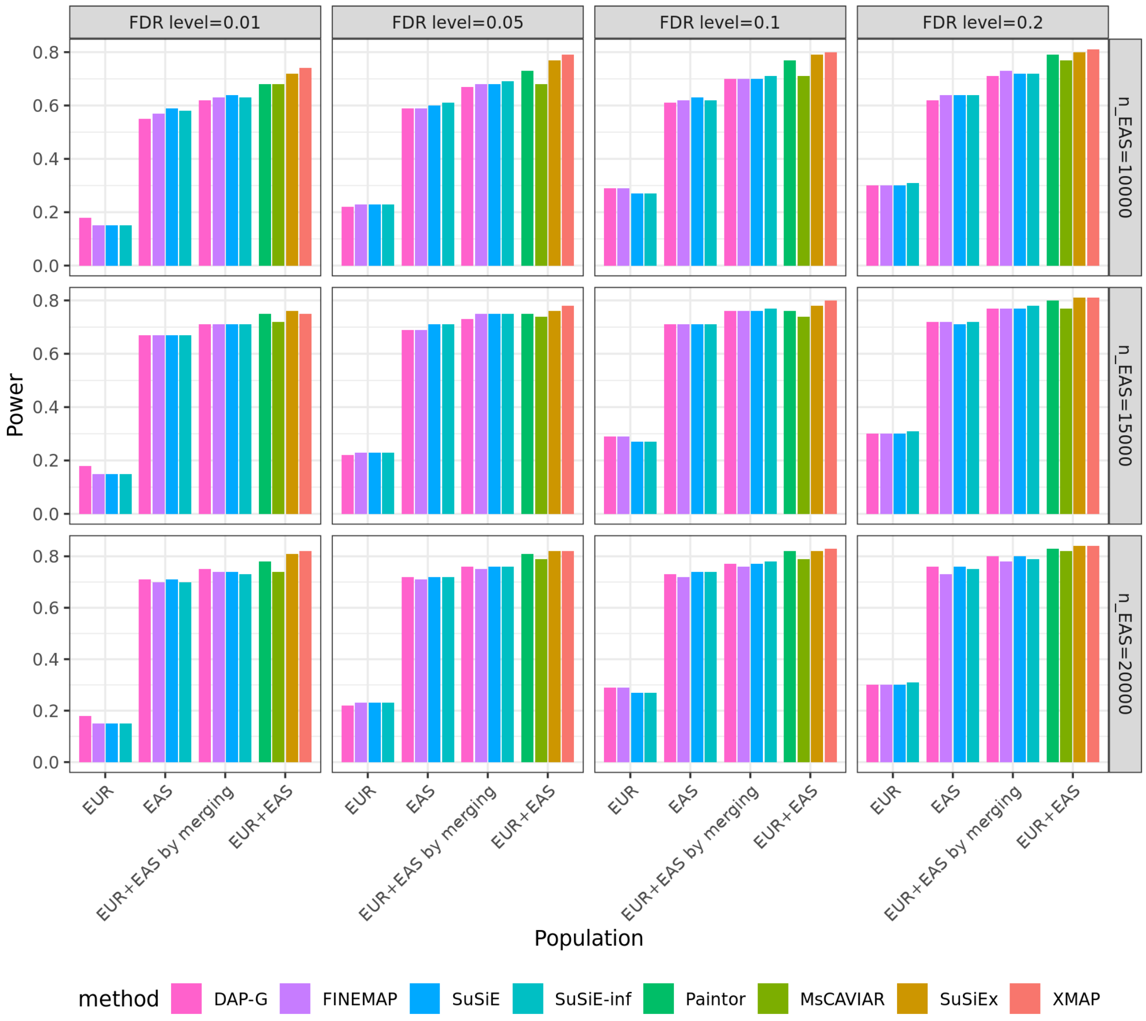


**Supplementary Figure 29:** Comparison of statistical power among DAP-G, FINEMAP, SuSiE, SuSiE-inf, Paintor, MsCAIVAR, SuSiEx, and XMAP in the setting without polygenic effects. We set $K_{true} = 2$, EUR sample size $n_2 = 20,000$, and varied EAS sample size $n_1 \in \{10,000,\ 15,000,\ 20,000\}$. Each of the $K_{true}$ causal SNPs explains 0.5% phenotypic variance.

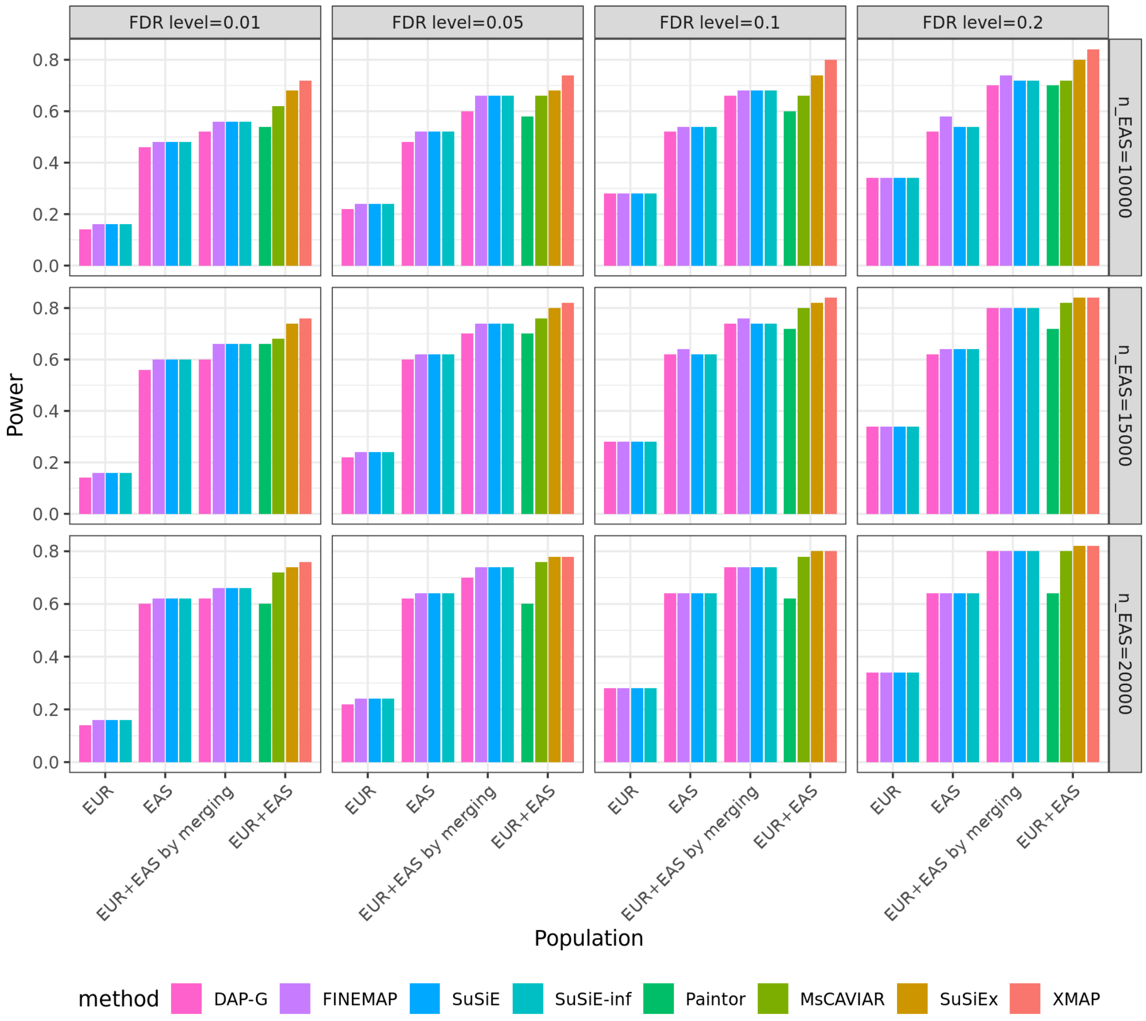


**Supplementary Figure 30:** Comparison of statistical power among DAP-G, FINEMAP, SuSiE, SuSiE-inf, Paintor, MsCAIVAR, SuSiEx, and XMAP in the setting without polygenic effects. We set $K_{true} = 1$, EUR sample size $n_2 = 20,000$, and varied EAS sample size $n_1 \in \{10,000,\ 15,000,\ 20,000\}$. Each of the $K_{true}$ causal SNPs explains 0.5% phenotypic variance.

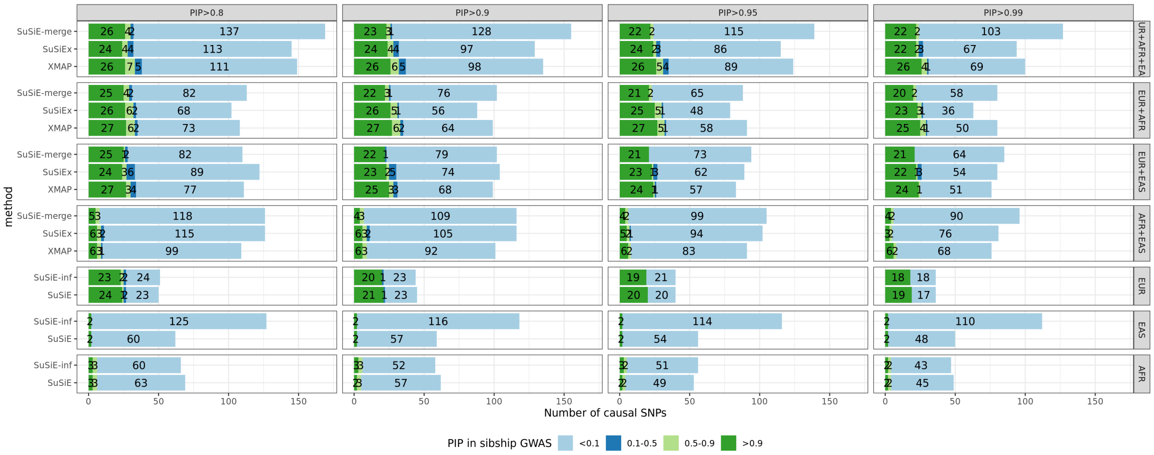


**Supplementary Figure 31:** Replication performance of putative causal SNPs identified by XMAP, SuSiEx, SuSiE-inf, and SuSiE. We included an ad-hoc method by merging the SNPs identified by SuSiE across populations (SuSiE-merge). XMAP is the overall winner with the highest replication rate under various PIP thresholds. A substantial proportion of SNPs identified by SuSiE-merge cannot be replicated because it includes a post-selection step that introduces false positives.

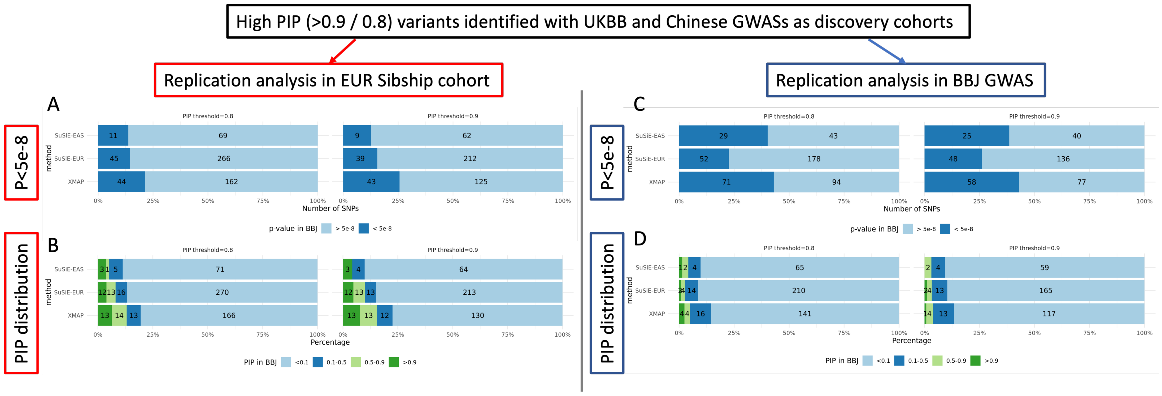


**Supplementary Figure 32:** Replication analysis of XMAP and SuSiE with $K = 15$ on height GWASs. Bar charts are shown for the fraction and number of fine-mapped SNPs with $p$-value$< 5 \times 10^{-8}$ in the replication cohorts of EUR Sibship (A) and BBJ GWAS (C), and the PIP distribution of fine-mapped SNPs in the replication cohorts of EUR Sibship (B) and BBJ GWAS (D).

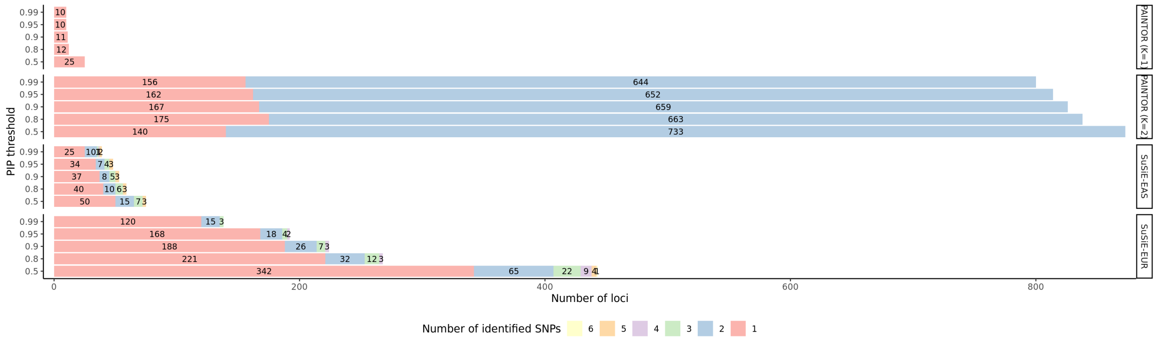


**Supplementary Figure 33:** Performance of PAINTOR (K=1), PAINTOR (K=2), SuSiE applied to EAS GWAS, and SuSiE applied to EUR GWAS in identifying multiple causal variants for height. Bar plots show the distributions of the number of putative causal SNPs under different PIP thresholds. PAINTOR had unstable performance when analyzing loci with thousands of SNPs. When K was set to 1 in PAINTOR (using the falg '-enumerate 1'), many loci had PIP=1 for all SNPs. We removed these loci when summarizing the results. When K was set to 2 in PAINTOR (using the falg '-enumerate 2'), many loci had exactly 2 SNPs with PIP=1, most of which could not be replicated in the Sibship GWAS (see Supplementary Figure34).

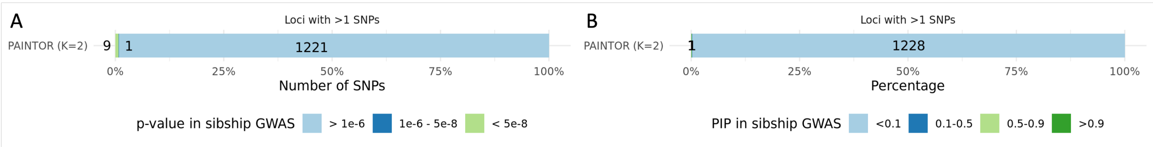


**Supplementary Figure 34:** Replication analysis of PAINTOR with K=2 on height GWASs. Bar charts are shown for the $p$-value (A) and PIP (B) distributions of putative causal SNPs in the replication cohort of EUR Sibship GWAS.

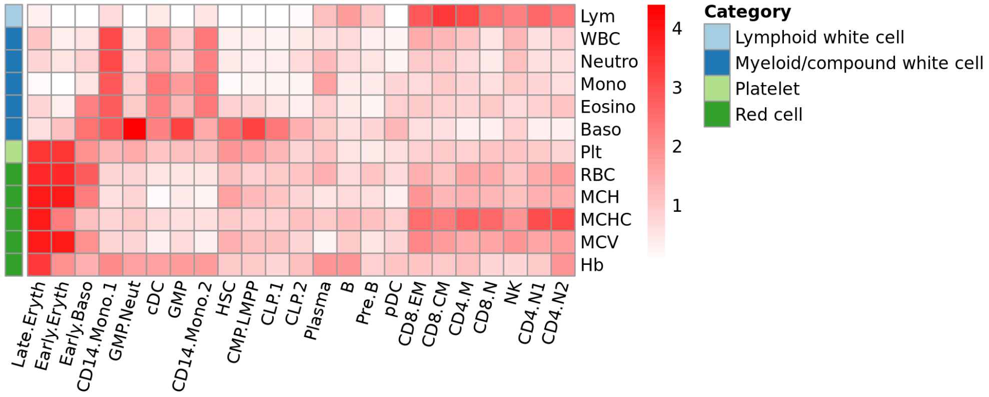


**Supplementary Figure 35:** The heat map showing the median TRS computed by SCAVENGE across 18 cell populations. The 12 blood traits are grouped into four clusters.

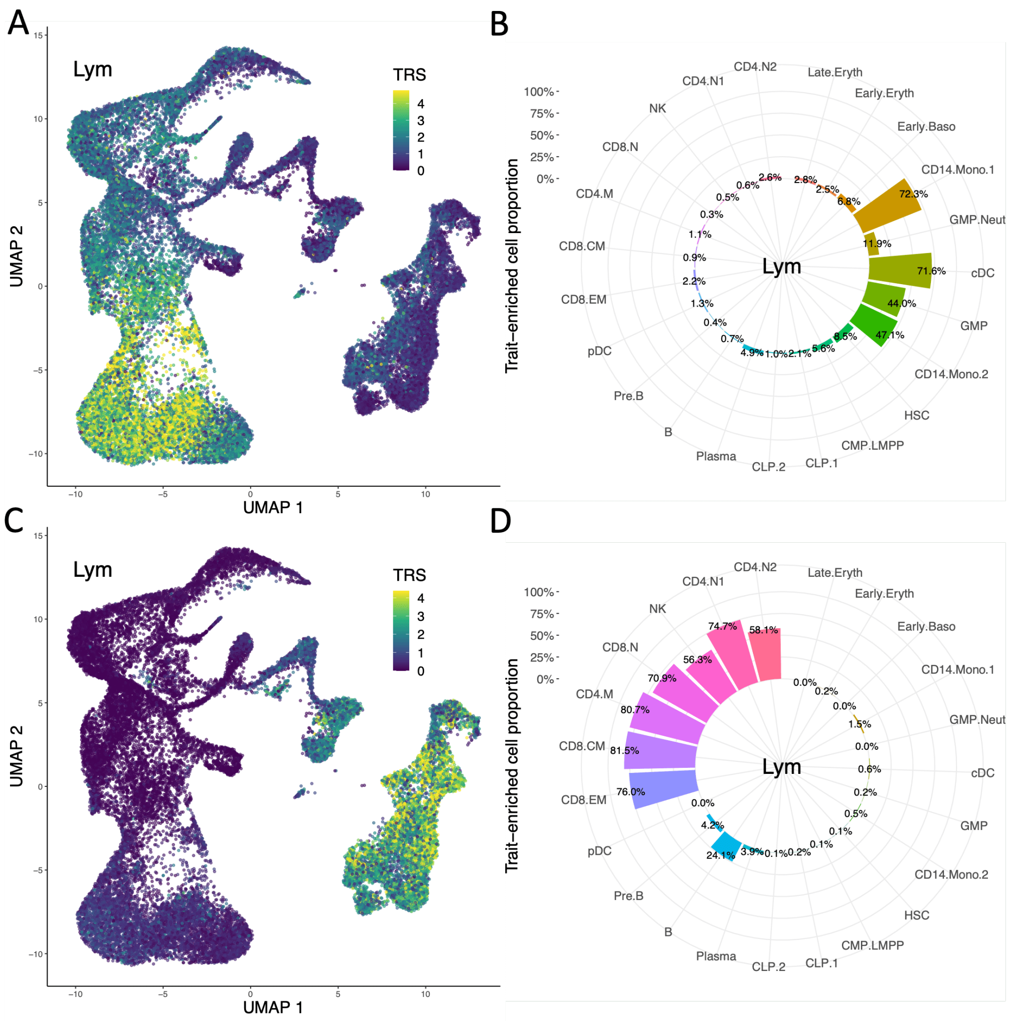


**Supplementary Figure 36:** Enrichment of the lymphocyte count in hematological populations using fine-mapped SNPs as input. The SCAVENGE TRS obtained by using the fine-mapping results of SuSiE in BBJ (A) and UKBB (C) are shown in the UMAP coordinates. The proportions of significantly enriched cells within each population obtained by using the fine-mapping results of SuSiE in BBJ (B) and UKBB (D).

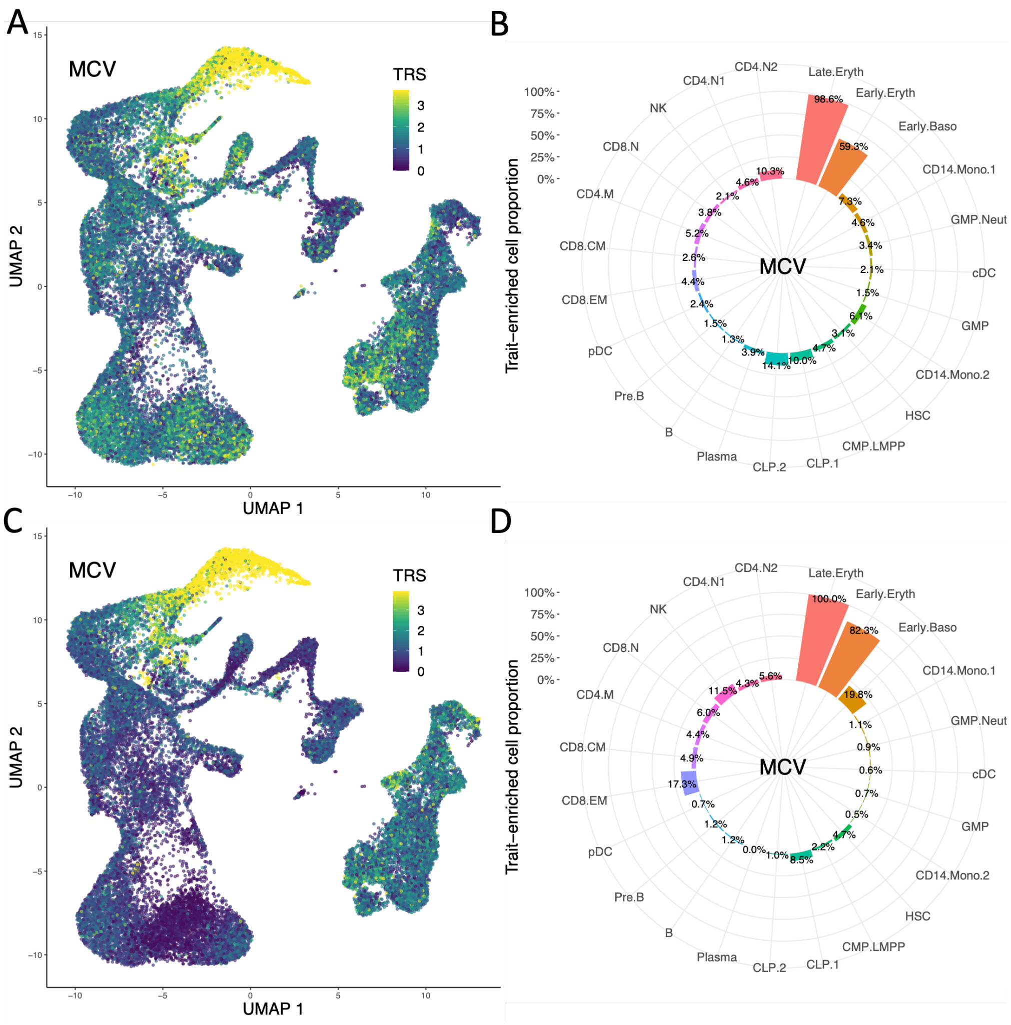


**Supplementary Figure 37:** Enrichment of the mean corpuscular volume in hematological populations using fine-mapped SNPs as input. The SCAVENGE TRS obtained by using the fine-mapping results of SuSiE in BBJ (A) and UKBB (C) are shown in the UMAP coordinates. The proportions of significantly enriched cells within each population obtained by using the fine-mapping results of SuSiE in BBJ (B) and UKBB (D).

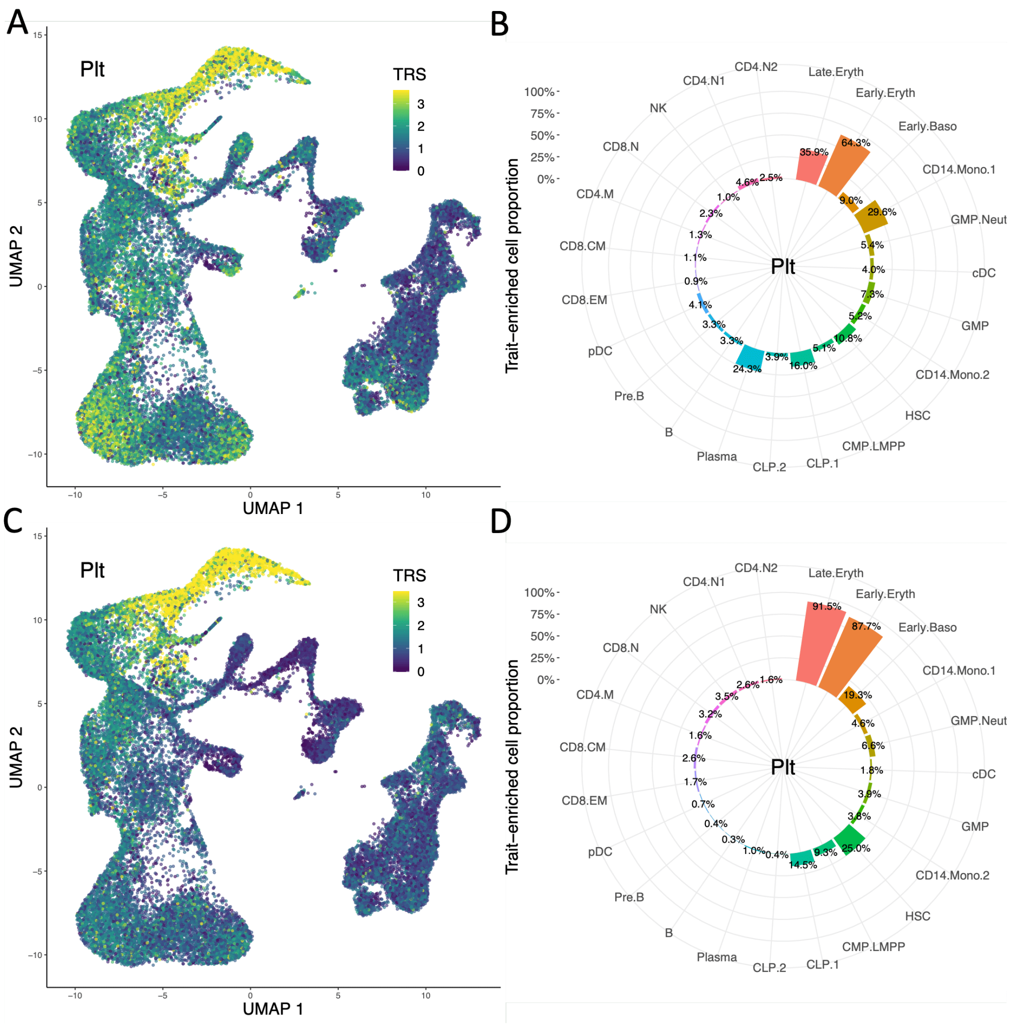


**Supplementary Figure 38:** Enrichment of the platelet count in hematological populations using fine-mapped SNPs as input. The SCAVENGE TRS obtained by using the fine-mapping results of SuSiE in BBJ (A) and UKBB (C) are shown in the UMAP coordinates. The proportions of significantly enriched cells within each population obtained by using the fine-mapping results of SuSiE in BBJ (B) and UKBB (D).

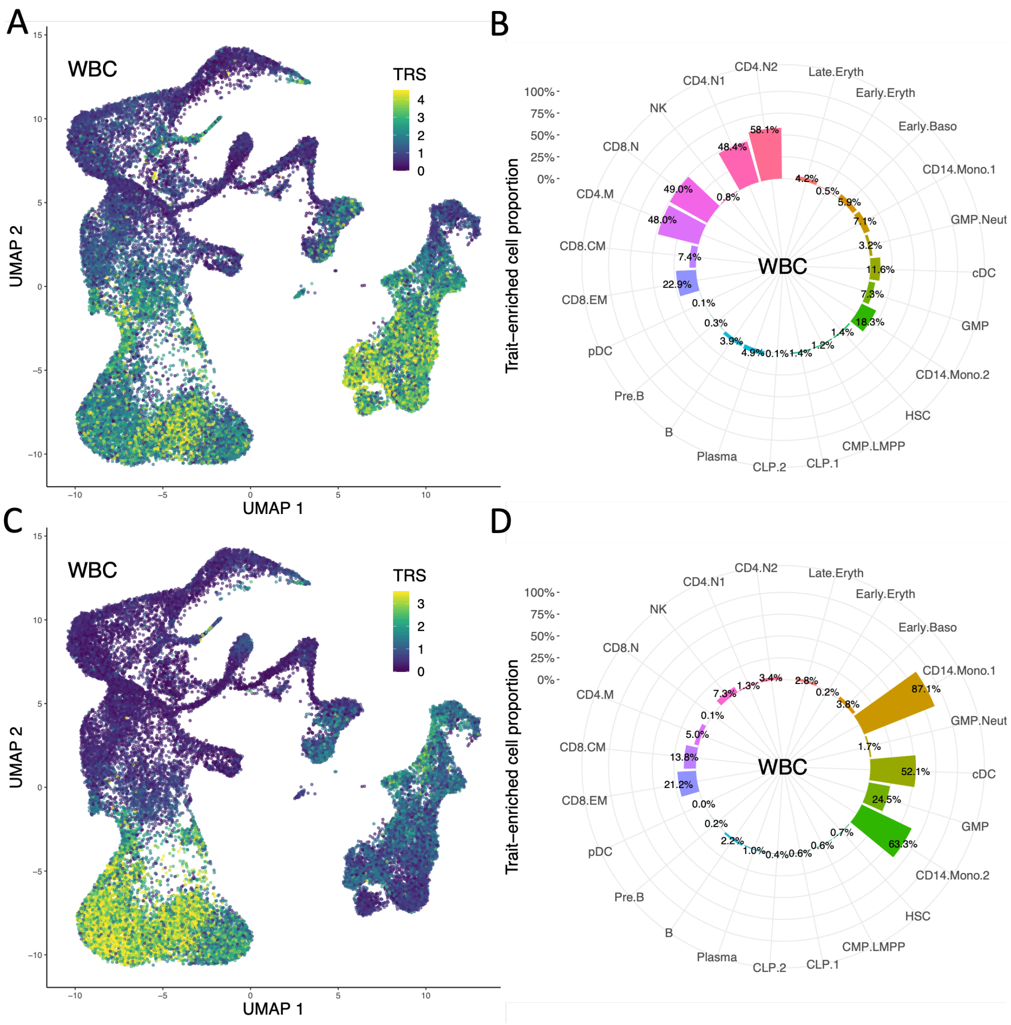

**Supplementary Figure 39:** Enrichment of the white blood cell count in hematological populations using fine-mapped SNPs as input. The SCAVENGE TRS obtained by using the fine-mapping results of SuSiE in BBJ (A) and UKBB (C) are shown in the UMAP coordinates. The proportions of significantly enriched cells within each population obtained by using the fine-mapping results of SuSiE in BBJ (B) and UKBB (D).

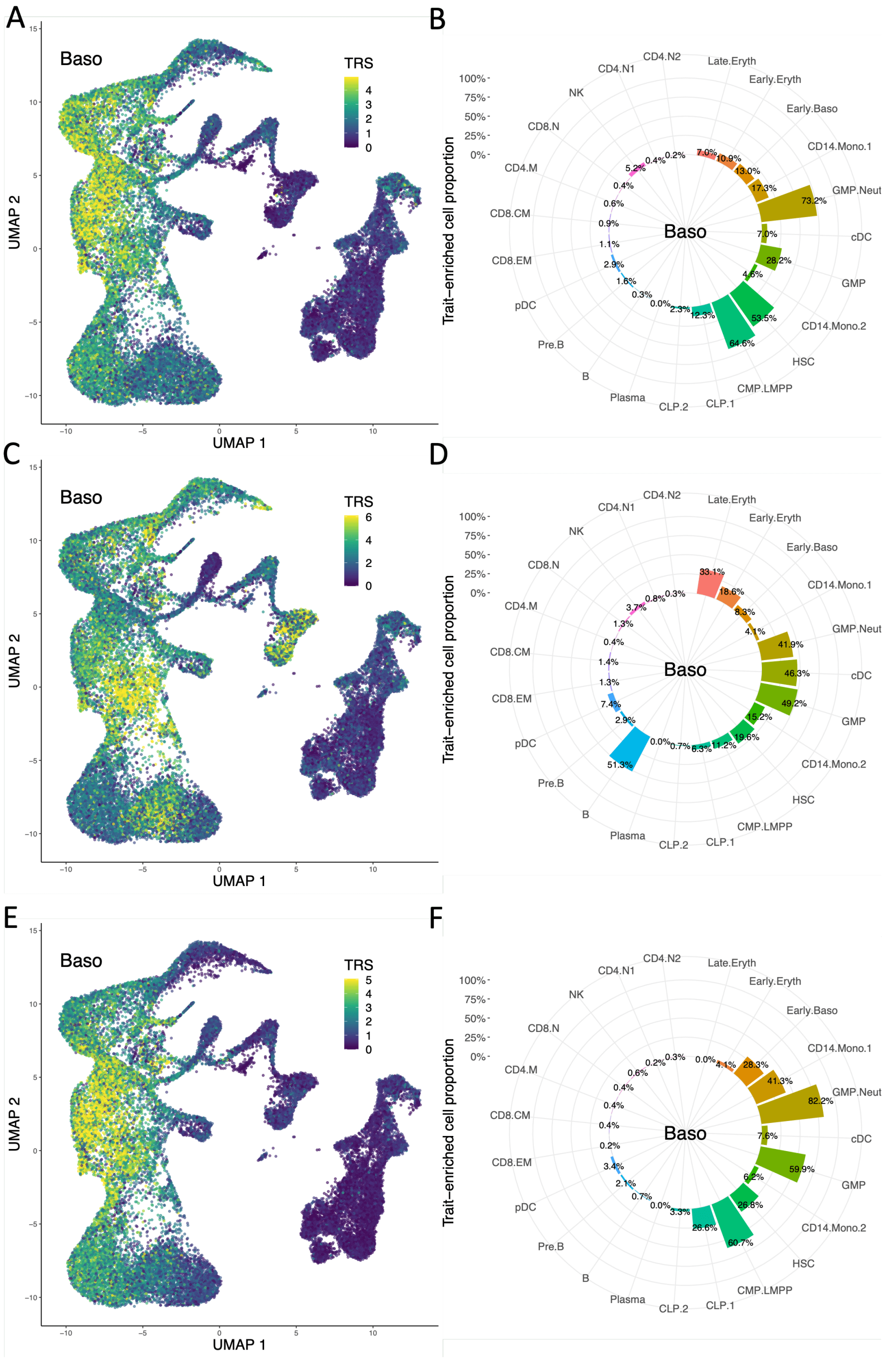


**Supplementary Figure 40:** Enrichment of the basophil count in hematological populations using fine-mapped SNPs as input. The SCAVENGE TRS obtained by using the fine-mapping results of XMAP (A) and SuSiE in BBJ (C) and UKBB (E) are shown in the UMAP coordinates. The proportions of significantly enriched cells within each population obtained by using the fine-mapping results of XMAP (B) and SuSiE in BBJ (D) and UKBB (F).

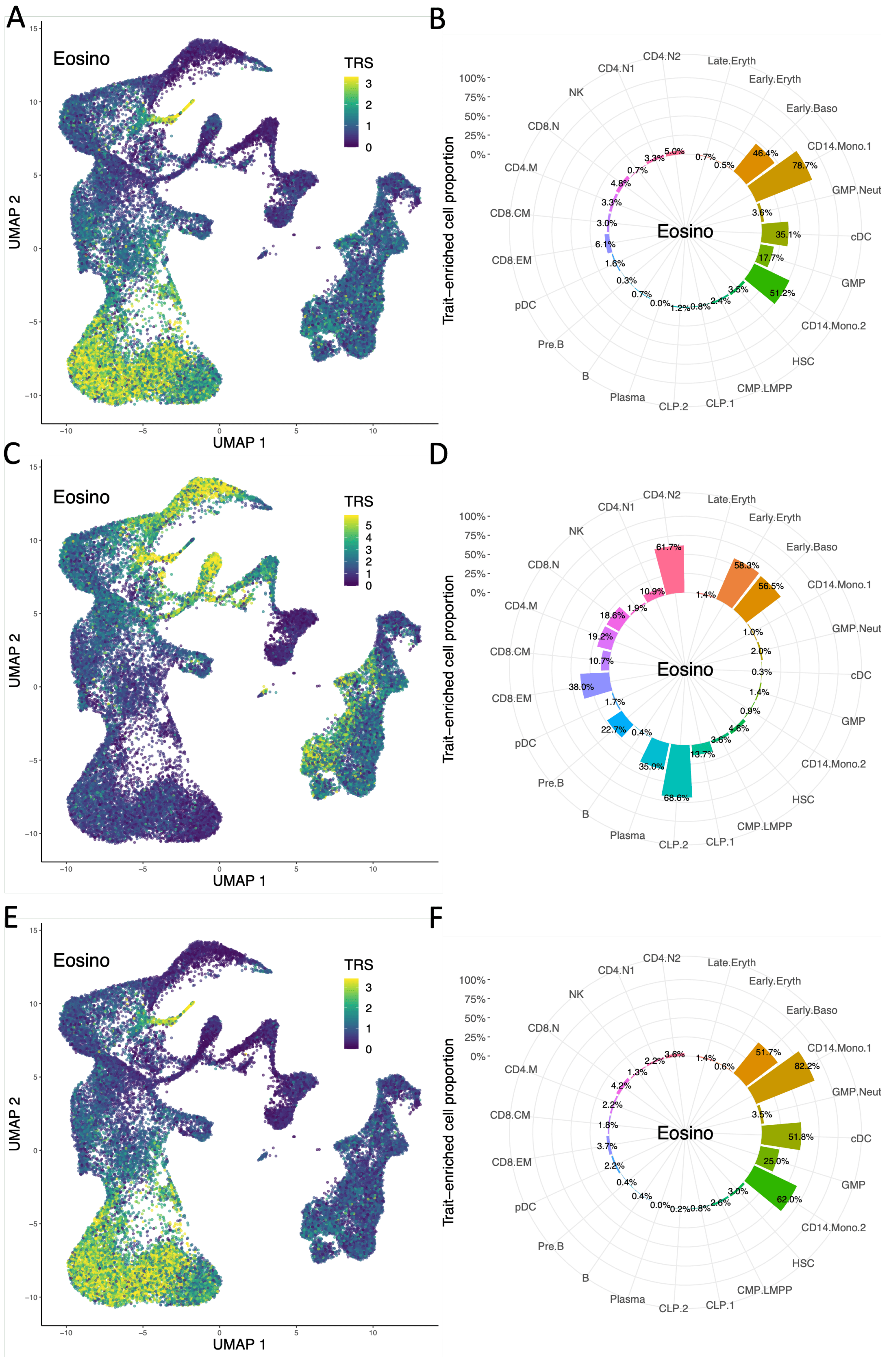


**Supplementary Figure 41:** Enrichment of the eosinophil count in hematological populations using fine-mapped SNPs as input. The SCAVENGE TRS obtained by using the fine-mapping results of XMAP (A) and SuSiE in BBJ (C) and UKBB (E) are shown in the UMAP coordinates. The proportions of significantly enriched cells within each population obtained by using the fine-mapping results of XMAP (B) and SuSiE in BBJ (D) and UKBB (F).

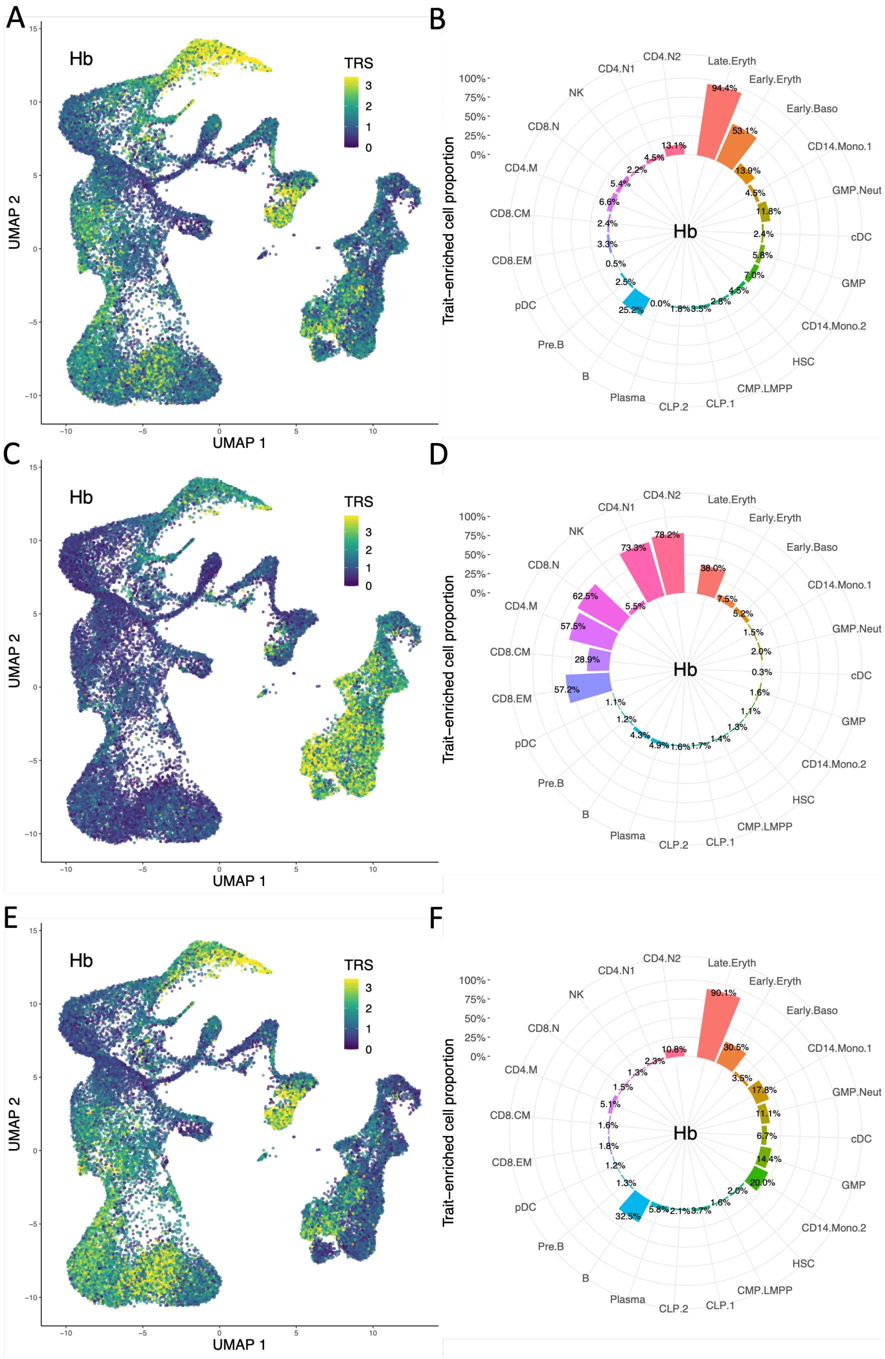

**Supplementary Figure 42:** Enrichment of the hemoglobin in hematological populations using fine-mapped SNPs as input. The SCAVENGE TRS obtained by using the fine-mapping results of XMAP (A) and SuSiE in BBJ (C) and UKBB (E) are shown in the UMAP coordinates. The proportions of significantly enriched cells within each population obtained by using the fine-mapping results of XMAP (B) and SuSiE in BBJ (D) and UKBB (F).

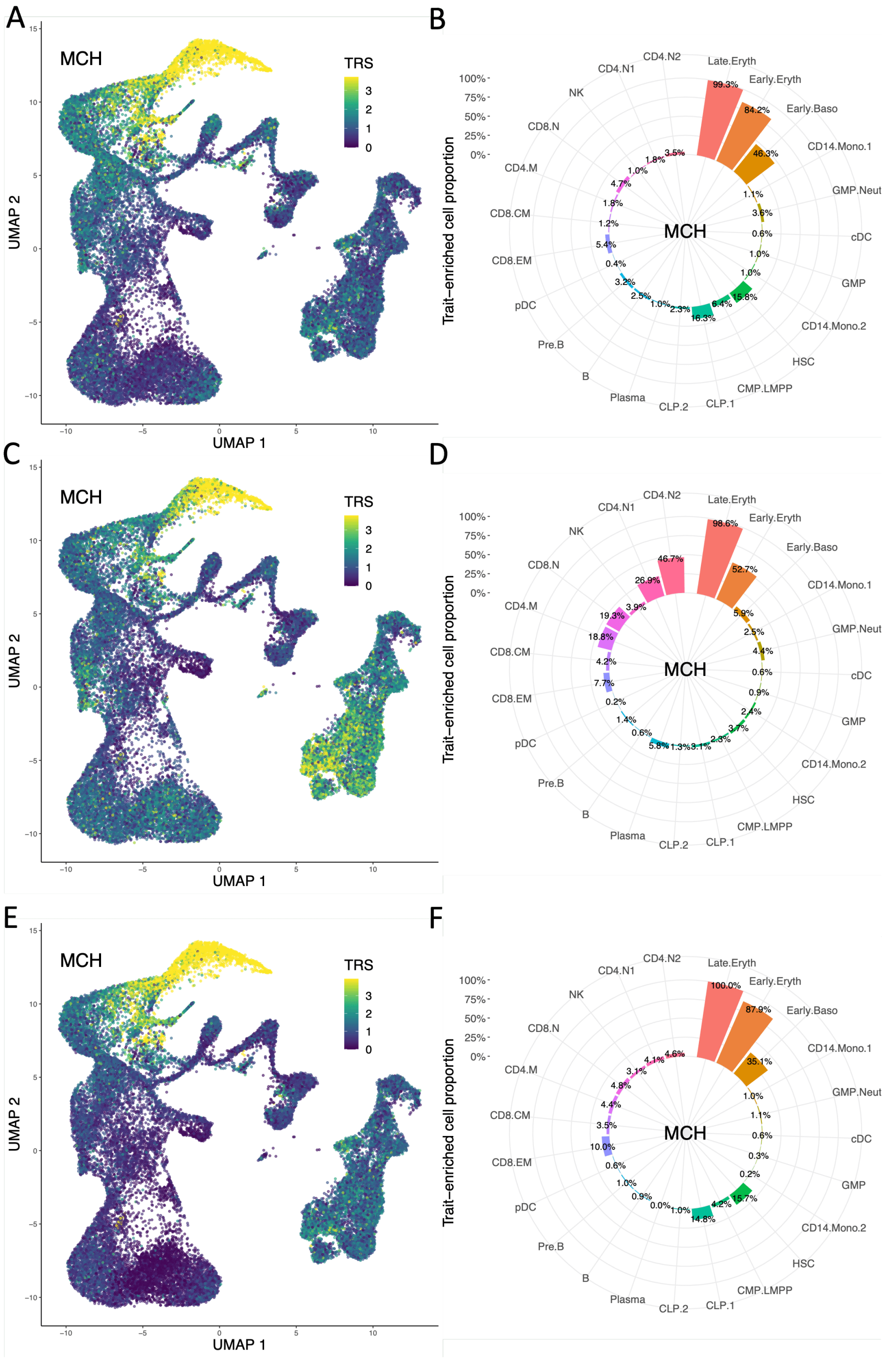


**Supplementary Figure 43:** Enrichment of the mean corpuscular hemoglobin in hematological populations using fine-mapped SNPs as input. The SCAVENGE TRS obtained by using the fine-mapping results of XMAP (A) and SuSiE in BBJ (C) and UKBB (E) are shown in the UMAP coordinates. The proportions of significantly enriched cells within each population obtained by using the fine-mapping results of XMAP (B) and SuSiE in BBJ (D) and UKBB (F).

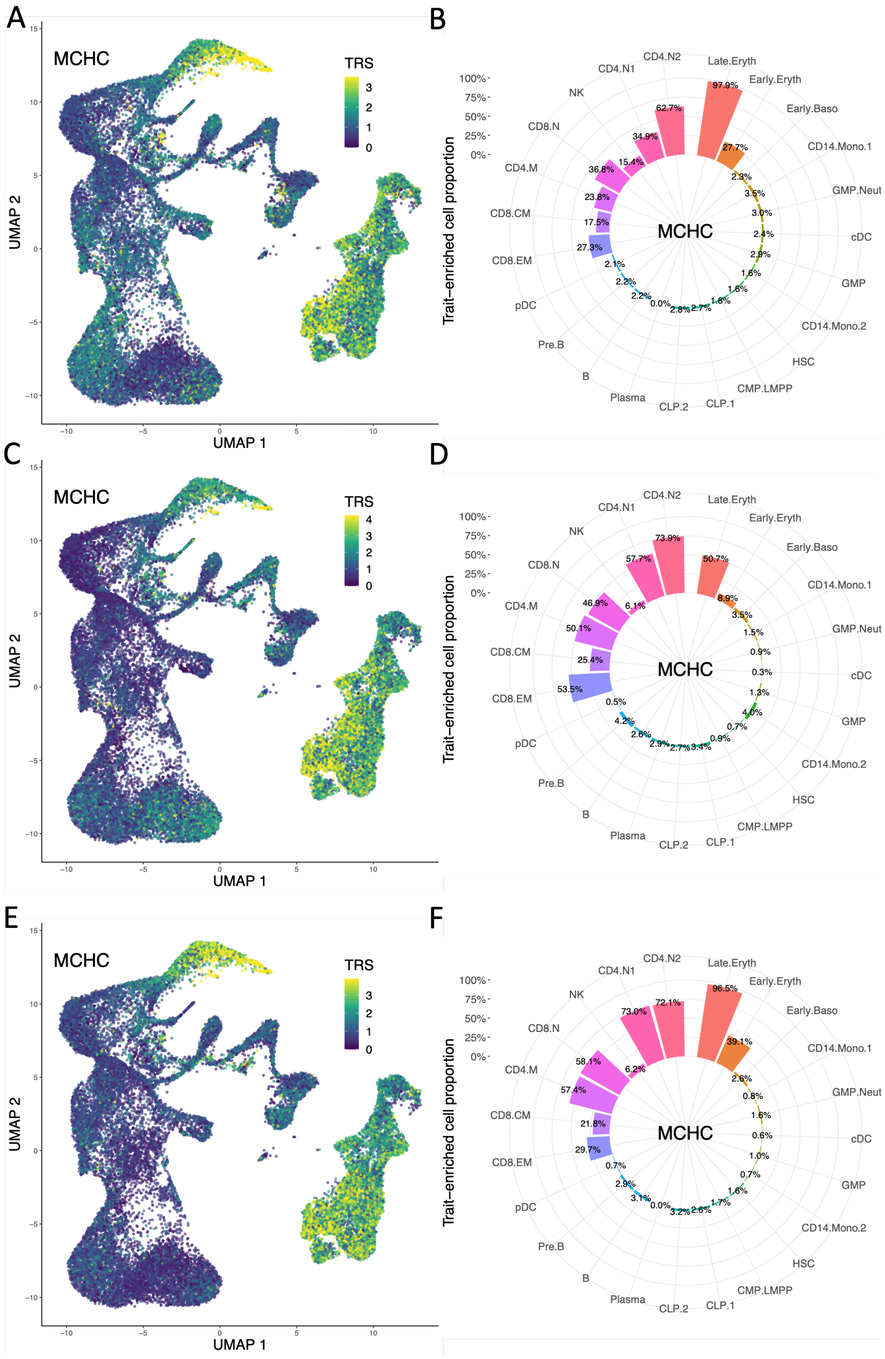


**Supplementary Figure 44:** Enrichment of the mean corpuscular hemoglobin concentration in hematological populations using fine-mapped SNPs as input. The SCAVENGE TRS obtained by using the fine-mapping results of XMAP (A) and SuSiE in BBJ (C) and UKBB (E) are shown in the UMAP coordinates. The proportions of significantly enriched cells within each population obtained by using the fine-mapping results of XMAP (B) and SuSiE in BBJ (D) and UKBB (F).

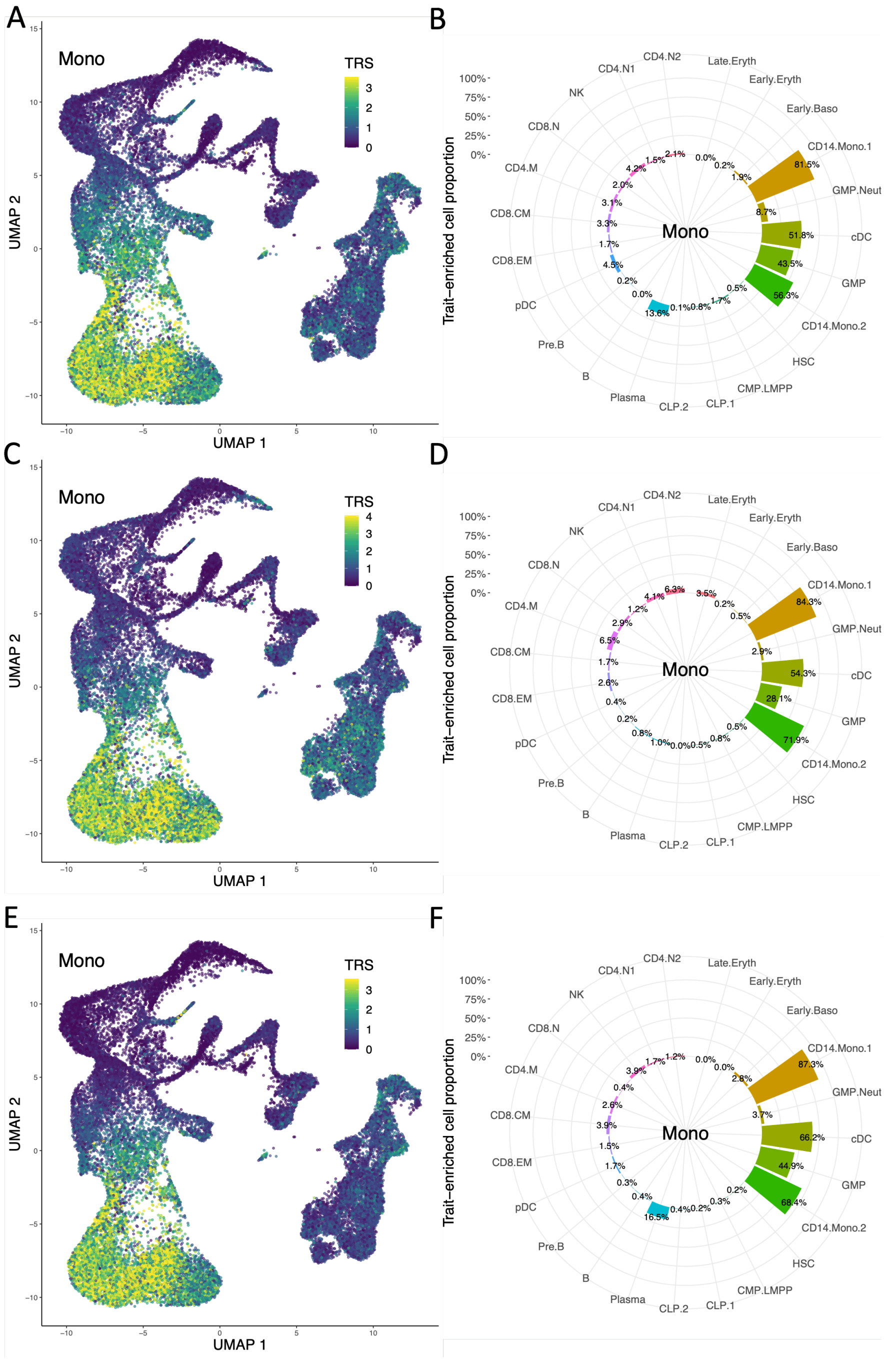


**Supplementary Figure 45:** Enrichment of the monocyte count in hematological populations using fine-mapped SNPs as input. The SCAVENGE TRS obtained by using the fine-mapping results of XMAP (A) and SuSiE in BBJ (C) and UKBB (E) are shown in the UMAP coordinates. The proportions of significantly enriched cells within each population obtained by using the fine-mapping results of XMAP (B) and SuSiE in BBJ (D) and UKBB (F).

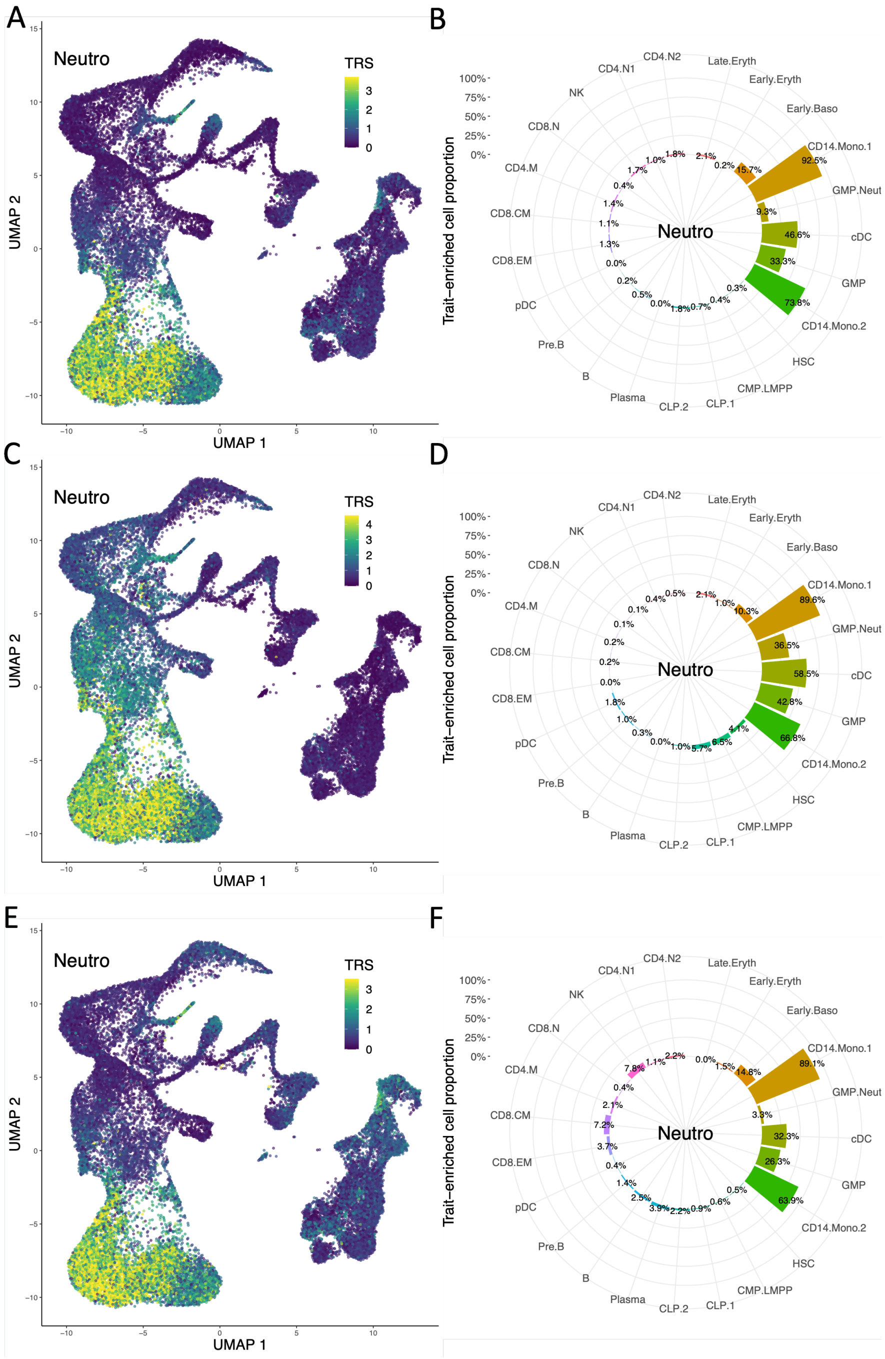


**Supplementary Figure 46:** Enrichment of the neutrophil count in hematological populations using fine-mapped SNPs as input. The SCAVENGE TRS obtained by using the fine-mapping results of XMAP (A) and SuSiE in BBJ (C) and UKBB (E) are shown in the UMAP coordinates. The proportions of significantly enriched cells within each population obtained by using the fine-mapping results of XMAP (B) and SuSiE in BBJ (D) and UKBB (F).

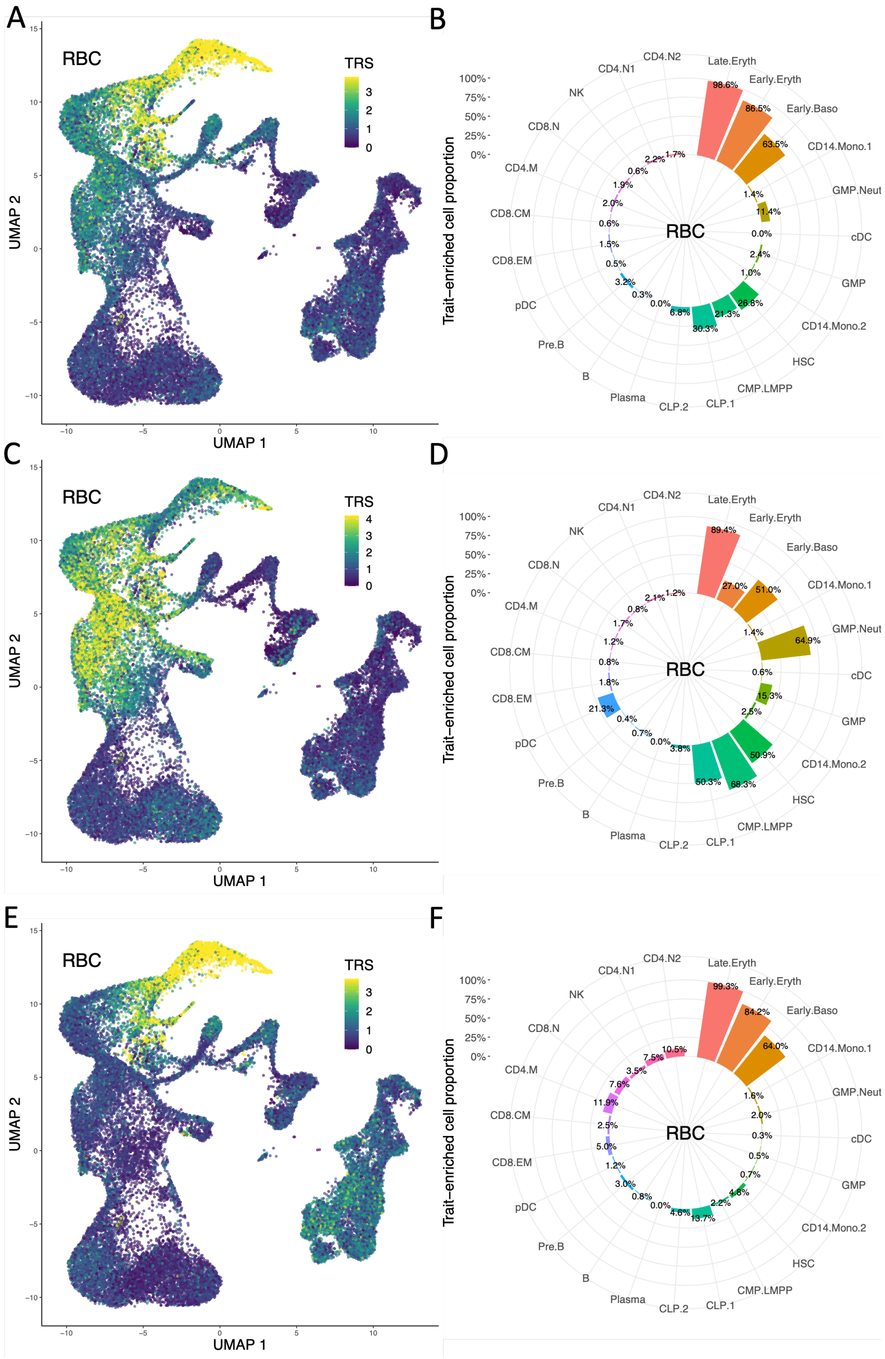


**Supplementary Figure 47:** Enrichment of the red blood cell count in hematological populations using fine-mapped SNPs as input. The SCAVENGE TRS obtained by using the fine-mapping results of XMAP (A) and SuSiE in BBJ (C) and UKBB (E) are shown in the UMAP coordinates. The proportions of significantly enriched cells within each population obtained by using the fine-mapping results of XMAP (B) and SuSiE in BBJ (D) and UKBB (F).

# 3 Supplementary Note

## 3.1 Simulations without polygenic effects and confounding bias

Besides the settings considered in the main text, we conducted additional simulations to investigate the performance of XMAP in the absence of either polygenic effects and confounding bias. In this simulation, we generated the causal effects with $\beta_{1k} \sim \mathcal{N}(0, \omega_1)$ and $\beta_{2k} \sim \mathcal{N}(0, \omega_2)$ for $k = 1, ..., K_{true}$, where $\omega_1$ and $\omega_2$ are the per-SNP heritabilities explained by the causal SNPs in populations 1 and 2, respectively, and we varied $\omega_1 = \omega_2 \in \{0.01, 0.005\}$. We generated quantitative phenotypes in the two populations with $\mathbf{y}_1 = \sum_{k=1}^{K_{true}} \mathbf{x}_{1[k]}\beta_{1k} + \mathbf{e}_2$ and $\mathbf{y}_2 = \sum_{k=1}^{K_{true}} \mathbf{x}_{2[k]}\beta_{2k} + \mathbf{e}_2$, where $\mathbf{x}_{1[k]}$ and $\mathbf{x}_{2[k]}$ are the columns of $\mathbf{X}_1$ and $\mathbf{X}_2$ corresponding to the $k$-th causal SNP, and $\mathbf{e}_1 \sim \mathcal{N}(\mathbf{0}, (1 - \omega_1 \times K_{true})\mathbf{I}_{n_1})$ and $\mathbf{e}_2 \sim \mathcal{N}(\mathbf{0}, (1 - \omega_2 \times K_{true})\mathbf{I}_{n_2})$ are independent noise in the two populations, respectively. The results are summarized in Supplementary Figures 23-30. Under this setting, the performance of XMAP was comparable to with SuSiEx, PAINTOR and MsCAVIAR.

## 3.2 Simulations with misspecified genetic effects

We conducted simulation studies to assess the performance of XMAP when the distributions of effect sizes are misspecified. We used the same real genotypes from the EUR and EAS cohorts and focused on the same region with $p = 500$ SNPs as described in the manuscript. The candidate causal SNPs were selected based on the criterion described in the original simulation. We set $K_{true} = 3$, EAS sample size $n_1 = 20,000$, and EUR sample size as $n_2 = 20,000$. To mimic the violation of XMAP assumptions on effect sizes, we considered scaled t-distribution denoted as $t(df, s)$, where $df$ and $s$ are degrees of freedom and variance of the scaled t-distribution, respectively. The parameter $df$ allows us to specify the discrepancy from the normal distribution and the parameter $s$ controls the strength of genetic effects. For the polygenic effects, we simulated the effect sizes with $\phi_{1j} \sim t(df, 0.005/500)$ and $\phi_{2j} \sim t(df, 0.005/500)$ for $j = 1, ..., 500$, where 0.005 is the total heritability contributed by polygenic effects of the 500 SNPs, with a per-SNP heritability $10^{-5}$. For causal effects, we simulated the effect sizes with $\beta_{1k} \sim t(df, 0.25/500)$ and $\beta_{2k} \sim t(df, 0.25/500)$, which means that each causal SNP has a $0.25/0.005 = 50$ fold per-SNP heritability enrichment compared to non-causal SNPs. We considered two $df$ settings: $df = 4$ and $df = 16$. We compared the performance of fine-mapping across 8 methods, including XMAP, XMAP with $\mathbf{\Omega} = \mathbf{0}$, SuSiEx, DAP-G, FINEMAP, SuSiE, SuSiE-inf, PAINTOR. MsCAVIAR was excluded because it is too time-consuming in the setting of $K_{true} = 3$. For each setting, we generated 50 replicates and identified causal variants by controlling the global FDR. When $df = 16$ (Supplementary Figure 13), XMAP had the best power and calibrated empirical FDR with stringent FDR thresholds (FDR $\leq 0.2$). With a slightly conservative PIP when FDR$\geq 0.3$, it still had the highest statistical power among compared methods. SuSiEx and XMAP with $\mathbf{\Omega} = \mathbf{0}$ were inflated because they do not account for the polygenic effects. SuSiE and SuSiE-inf were deflated because they only used GWAS from a single population. DAP-G, FINEMAP, and PAINTOR had satisfactory performance in FDR control with stringent FDR thresholds but low

statistical power. When $df = 4$, the true distribution of effect sizes is substantially different from the normal distribution (Supplementary Figure 14). In this setting, XMAP could still achieve good calibration of FDR and high statistical power. Although DAP-G had high power with a less stringent FDR threshold ($\geq 0.3$), it had an inflated empirical FDR in these settings. Overall, XMAP performed reasonably well in controlling false positives while achieving high statistical power when the effect size distribution is misspecified. This evidence suggests that XMAP is robust and reliable to misspecified effect size distributions in real GWAS data.

## 3.3 Compared methods

We compared XMAP with existing fine-mapping approaches in the simulation analysis. In the main analysis of XMAP, we estimated the polygenic parameters $\mathbf{\Omega}$ and inflation constants $c_1$ and $c_2$ with bivariate LDSC, and considered two settings of $K$: $K = 5$ and $K = 10$. For single-population approaches, we considered FINEMAP, Dap-G, and SuSiE. We ran FINEMAP with the shot gun stochastic search algorithm by using the flag '–sss' and set '–n-causal-snps' as the default value 5. For SuSiE, SuSiE-inf and SuSiEx, we set the maximum number of causal signals $L = 5$. We also included two cross-population approaches, MsCAVIAR and PAINTOR, in our analysis. We ran MsCAVIAR with the flag '-c $K_{true}$', and ran PAINTOR with the flag '-enumerate $K_{true}$'.

In real data analysis, we used $K = 10$ in XMAP for the main analysis and conducted sensitivity analysis with $K = 15$. For SuSiE, SuSiE-inf and SuSiEx, we set $L = 10$. When applying PAINTOR to screen all loci on the genome, we considered two settings: '-enumerate 1' and '-enumerate 2'. In the example presented in Figure 6, we used the flags '-enumerate 2' in PAINTOR and '-c 2' in MsCAVIAR.

## 3.4 Derivation of the covariance of $\boldsymbol{\epsilon}_1$ and $\boldsymbol{\epsilon}_2$

We first derive the first moment of $\mathbf{b}_1$ and $\mathbf{b}_2$:

$$\begin{aligned}\mathbb{E}\left[\mathbf{b}_1\right] &= \mathbb{E}\left[\sum_{k=1}^{K} \boldsymbol{\gamma}_k \beta_{1k}\right] = \sum_{k=1}^{K} \mathbb{E}\left[\mathbb{E}\left[\boldsymbol{\gamma}_k \beta_{1k} | \boldsymbol{\gamma}_k\right]\right] = \mathbf{0} \\ \mathbb{E}\left[\mathbf{b}_2\right] &= \mathbb{E}\left[\sum_{k=1}^{K} \boldsymbol{\gamma}_k \beta_{2k}\right] = \sum_{k=1}^{K} \mathbb{E}\left[\mathbb{E}\left[\boldsymbol{\gamma}_k \beta_{2k} | \boldsymbol{\gamma}_k\right]\right] = \mathbf{0}.\end{aligned} \tag{1}$$

By applying the law of total variance, the second moment of $\mathbf{b}_1$ can be obtained as:

$$
\begin{aligned}
&\mathbb{E}\left[\mathbf{b}_1\mathbf{b}_1^T\right] = \mathrm{Var}\left[\mathbf{b}_1\right] \\
=&\mathrm{Var}\left[\sum_{k=1}^{K}\mathbb{E}\left[\boldsymbol{\gamma}_k\beta_{1k}|\boldsymbol{\gamma}_k\right]\right] + \mathbb{E}\left[\sum_{k=1}^{K}\mathrm{Var}\left[\boldsymbol{\gamma}_k\beta_{1k}|\boldsymbol{\gamma}_k\right]\right] \\
=&\mathbf{0} + \frac{1}{p}\sum_{k=1}^{K}\sum_{j=1}^{p}\sigma_{1k}^2\mathbf{I}_p \\
=&\sum_{k=1}^{K}\sigma_{1k}^2\mathbf{I}_p.
\end{aligned}
\tag{2}
$$

Similarly, we have $\mathbb{E}\left[\mathbf{b}_2\mathbf{b}_2^T\right] = \mathrm{Var}\left[\mathbf{b}_2\right] = \sum_{k=1}^{K}\sigma_{2k}^2\mathbf{I}_p$. With the above equations, we can obtain the first moments of $\hat{\mathbf{b}}_1$ and $\hat{\mathbf{b}}_2$:

$$
\begin{aligned}
\mathbb{E}\left[\hat{\mathbf{b}}_1\right] &= \mathbb{E}\left[\mathbb{E}\left[\hat{\mathbf{b}}_1|\mathbf{b}_1,\boldsymbol{\phi}_1\right]\right] = \mathbb{E}\left[\mathbb{E}\left[\mathbf{R}_1\mathbf{b}_1 + \mathbf{R}_1\boldsymbol{\phi}_1|\mathbf{b}_1,\boldsymbol{\phi}_1\right]\right] = \mathbf{0} \\
\mathbb{E}\left[\hat{\mathbf{b}}_2\right] &= \mathbb{E}\left[\mathbb{E}\left[\hat{\mathbf{b}}_2|\mathbf{b}_2,\boldsymbol{\phi}_2\right]\right] = \mathbb{E}\left[\mathbb{E}\left[\mathbf{R}_2\mathbf{b}_2 + \mathbf{R}_2\boldsymbol{\phi}_2|\mathbf{b}_2,\boldsymbol{\phi}_2\right]\right] = \mathbf{0}.
\end{aligned}
\tag{3}
$$

Next, we derive the variance-covariance matrix of of $\hat{\mathbf{b}}_1$. The covariance between the $j$-th and the $j'$-th elements of $\hat{\mathbf{b}}_1$ is given as

$$
\begin{aligned}
&\mathrm{Cov}[\hat{\mathbf{b}}_{1j}, \hat{\mathbf{b}}_{1j'}] = \mathbb{E}\left[\hat{\mathbf{b}}_{1j}\hat{\mathbf{b}}_{1j'}\right]\\
=&\mathbb{E}\left[\mathbb{E}\left[\hat{\mathbf{b}}_{1j}\hat{\mathbf{b}}_{1j'}|\mathbf{X}_1\right]\right]\\
=&\mathbb{E}\left[\mathbb{E}\left[\frac{1}{n_1^2}\mathbf{x}_{1j}^T\mathbf{y}_1\mathbf{y}_1^T\mathbf{x}_{1j'}|\mathbf{X}_1\right]\right]\\
=&\mathbb{E}\left[\mathbb{E}\left[\frac{1}{n_1^2}\mathbf{x}_{1j}^T(\mathbf{X}_1\mathbf{b}_1+\mathbf{X}_1\boldsymbol{\phi}_1+\mathbf{e}_1)(\mathbf{X}_1\mathbf{b}_1+\mathbf{X}_1\boldsymbol{\phi}_1+\mathbf{e}_1)^T\mathbf{x}_{1j'}|\mathbf{X}_1\right]\right]\\
=&\mathbb{E}\left[\mathbb{E}\left[\frac{1}{n_1^2}\mathbf{x}_{1j}^T(\mathbf{X}_1\mathbf{b}_1\mathbf{b}_1^T\mathbf{X}_1^T+\mathbf{X}_1\boldsymbol{\phi}_1\boldsymbol{\phi}_1^T\mathbf{X}_1^T+\mathbf{e}_1\mathbf{e}_1^T+2\mathbf{X}_1\mathbf{b}_1\boldsymbol{\phi}_1^T\mathbf{X}_1^T+2\mathbf{X}_1\mathbf{b}_1\mathbf{e}_1^T+2\mathbf{X}_1\boldsymbol{\phi}_1\mathbf{e}_1^T)\mathbf{x}_{1j'}|\mathbf{X}_1\right]\right]\\
=&\mathbb{E}\left[\frac{1}{n_1^2}\mathbf{x}_{1j}^T\mathbf{X}_1\mathbb{E}\left[\mathbf{b}_1\mathbf{b}_1^T\right]\mathbf{X}_1^T\mathbf{x}_{1j'}+\frac{1}{n_1^2}\mathbf{x}_{1j}^T\mathbf{X}_1\mathbb{E}\left[\boldsymbol{\phi}_1\boldsymbol{\phi}_1^T\right]\mathbf{X}_1^T\mathbf{x}_{1j'}+\frac{1}{n_1^2}\mathbf{x}_{1j}^T\mathbb{E}\left[\mathbf{e}_1\mathbf{e}_1^T\right]\mathbf{x}_{1j'}\right]\\
\overset{①}{=}&\frac{1}{n_1^2}(\sum_{k=1}^K\sigma_{k1}^2)\mathbb{E}\left[\mathbf{x}_{1j}^T\mathbf{X}_1\mathbf{X}_1^T\mathbf{x}_{1j'}\right]+\frac{1}{n_1^2}\omega_1\mathbb{E}\left[\mathbf{x}_{1j}^T\mathbf{X}_1\mathbf{X}_1^T\mathbf{x}_{1j'}\right]+\frac{1}{n_1^2}\sigma_{\mathbf{e}_1}^2\mathbb{E}\left[\mathbf{x}_{1j}^T\mathbf{x}_{1j'}\right]\\
=&(\sum_{k=1}^K\sigma_{k1}^2+\omega_1)\mathbb{E}\left[\mathbf{x}_{1j}^T\mathbf{X}_1\mathbf{X}_1^T\mathbf{x}_{1j'}/n_1^2\right]+\frac{1}{n_1}\sigma_{\mathbf{e}_1}^2r_{1jj'}\\
\overset{②}{\approx}&(\sum_{k=1}^K\sigma_{k1}^2+\omega_1)\sum_{l=1}^p r_{1jl}r_{1j'l}+\frac{p}{n_1}(\sum_{k=1}^K\sigma_{k1}^2+\omega_1)r_{1jj'}+\frac{1}{n_1}\sigma_{\mathbf{e}_1}^2r_{1jj'}\\
\overset{③}{=}&(\sum_{k=1}^K\sigma_{k1}^2+\omega_1)\sum_{l=1}^p r_{1jl}r_{1j'l}+\frac{1}{n_1}r_{1jj'}\\
\overset{④}{\approx}&(\sum_{k=1}^K\sigma_{k1}^2+\omega_1)\sum_{l=1}^p r_{1jl}r_{1j'l}+\hat{s}_{1j}\hat{s}_{1j'}r_{1jj'},
\end{aligned}
\tag{4}
$$

where we have used Supplementary Equation (2) for ①, approximated ② with $\mathbb{E}\left[\mathbf{x}_{1j}^T\mathbf{X}_1\mathbf{X}_1^T\mathbf{x}_{1j'}/n_1^2\right] = \mathbb{E}\left[\sum_{l=1}^p\mathbf{x}_{1j}^T\mathbf{x}_{1l}\mathbf{x}_{1l}^T\mathbf{x}_{1j'}/n_1^2\right] \approx \sum_{l=1}^p r_{1jl}r_{1j'l}+r_{1jj'}/n_1$, obtained ③ from $\mathrm{Var}(y_{1i}) = p\sum_{k=1}^K\sigma_{k1}^2 + p\omega_1+\sigma_{\mathbf{e}_1}^2 = 1$, and the approximation ④ is granted by Equations (6). Similarly, we have $\mathrm{Cov}[\hat{\mathbf{b}}_{2j}, \hat{\mathbf{b}}_{2j'}] \approx (\sum_{k=1}^K\sigma_{k2}^2+\omega_2)\sum_{l=1}^p r_{2jl}r_{2j'l}+\hat{s}_{2j}\hat{s}_{2j'}r_{2jj'}$. The covariance between elements

of $\hat{\mathbf{b}}_1$ and $\hat{\mathbf{b}}_2$ can be derived as

$$
\begin{aligned}
&\text{Cov}[\hat{\mathbf{b}}_{1j}, \hat{\mathbf{b}}_{2j'}] = \mathbb{E}\left[\hat{\mathbf{b}}_{1j}\hat{\mathbf{b}}_{2j'}\right] \\
=&\mathbb{E}\left[\mathbb{E}\left[\hat{\mathbf{b}}_{1j}\hat{\mathbf{b}}_{2j'}|\mathbf{X}_1, \mathbf{X}_2\right]\right] \\
=&\mathbb{E}\left[\mathbb{E}\left[\frac{1}{n_1n_2}\mathbf{x}_{1j}^T\mathbf{y}_1\mathbf{y}_2^T\mathbf{x}_{2j'}|\mathbf{X}_1, \mathbf{X}_2\right]\right] \\
=&\mathbb{E}\left[\mathbb{E}\left[\frac{1}{n_1n_2}\mathbf{x}_{1j}^T(\mathbf{X}_1\mathbf{b}_1 + \mathbf{X}_1\boldsymbol{\phi}_1 + \mathbf{e}_1)(\mathbf{X}_2\mathbf{b}_2 + \mathbf{X}_2\boldsymbol{\phi}_2 + \mathbf{e}_2)^T\mathbf{x}_{2j'}|\mathbf{X}_1, \mathbf{X}_2\right]\right] \\
=&\mathbb{E}\left[\mathbb{E}\left[\frac{1}{n_1n_2}\mathbf{x}_{1j}^T(\mathbf{X}_1\mathbf{b}_1\mathbf{b}_2^T\mathbf{X}_2^T + \mathbf{X}_1\boldsymbol{\phi}_1\boldsymbol{\phi}_2^T\mathbf{X}_2^T + \mathbf{e}_1\mathbf{e}_2^T|\mathbf{X}_1, \mathbf{X}_2\right]\right] \\
=&\mathbb{E}\left[\frac{1}{n_1n_2}\mathbf{x}_{1j}^T\mathbf{X}_1\mathbb{E}\left[\mathbf{b}_1\mathbf{b}_2^T\right]\mathbf{X}_2^T\mathbf{x}_{2j'} + \frac{1}{n_1n_2}\mathbf{x}_{1j}^T\mathbf{X}_1\mathbb{E}\left[\boldsymbol{\phi}_1\boldsymbol{\phi}_2^T\right]\mathbf{X}_2^T\mathbf{x}_{2j'} + \frac{1}{n_1n_2}\mathbf{x}_{1j}^T\mathbb{E}\left[\mathbf{e}_1\mathbf{e}_2^T\right]\mathbf{x}_{2j'}\right] \\
\overset{①}{=}&\frac{1}{n_1n_2}(\sum_{k=1}^K\sigma_{k12}^2)\mathbb{E}\left[\mathbf{x}_{1j}^T\mathbf{X}_1\mathbf{X}_2^T\mathbf{x}_{2j'}\right] + \frac{1}{n_1n_2}\omega_{12}\mathbb{E}\left[\mathbf{x}_{1j}^T\mathbf{X}_1\mathbf{X}_2^T\mathbf{x}_{2j'}\right] \\
=&(\sum_{k=1}^K\sigma_{k12}^2 + \omega_{12})\mathbb{E}\left[\mathbf{x}_{1j}^T\mathbf{X}_1\mathbf{X}_2^T\mathbf{x}_{2j'}/(n_1n_2)\right] \\
=&(\sum_{k=1}^K\sigma_{k12}^2 + \omega_{12})\sum_{l=1}^p r_{1jl}r_{2j'l},
\end{aligned}
\tag{5}
$$

where the equation ① is obtained given the fact that $\mathbb{E}\left[\mathbf{e}_1\mathbf{e}_2^T\right] = \mathbf{0}$ because GWAS samples from two populations are independent. With above relationships, the variance-covariance matrices of $\hat{\mathbf{b}}_1$ and $\hat{\mathbf{b}}_2$ can be obtained as

$$
\text{Var}\begin{bmatrix}\hat{\mathbf{b}}_1\\ \hat{\mathbf{b}}_2\end{bmatrix} = \mathcal{N}\left(\mathbf{0}, \begin{bmatrix}(\sum_{k=1}^K\sigma_{k1}^2 + \omega_1)\mathbf{R}_1^2 + \hat{\mathbf{S}}_1\mathbf{R}_1\hat{\mathbf{S}}_1 & (\sum_{k=1}^K\sigma_{k12}^2 + \omega_{12})\mathbf{R}_1\mathbf{R}_2\\ (\sum_{k=1}^K\sigma_{k12}^2 + \omega_{12})\mathbf{R}_1\mathbf{R}_2 & (\sum_{k=1}^K\sigma_{k2}^2 + \omega_2)\mathbf{R}_2^2 + \hat{\mathbf{S}}_2\mathbf{R}_2\hat{\mathbf{S}}_2\end{bmatrix}\right). \tag{6}
$$

Based on model (8), the variance-covariance matrices of $\boldsymbol{\epsilon}_1$ and $\boldsymbol{\epsilon}_2$ can be obtained as:

$$
\begin{aligned}
\text{Var}\begin{bmatrix}\boldsymbol{\epsilon}_1\\ \boldsymbol{\epsilon}_2\end{bmatrix} =&\text{Var}\begin{bmatrix}\hat{\mathbf{b}}_1\\ \hat{\mathbf{b}}_2\end{bmatrix} - \text{Var}\begin{bmatrix}\mathbf{R}_1(\sum_{k=1}^K\boldsymbol{\gamma}_k\beta_{1k} + \boldsymbol{\phi}_1)\\ \mathbf{R}_2(\sum_{k=1}^K\boldsymbol{\gamma}_k\beta_{2k} + \boldsymbol{\phi}_2)\end{bmatrix} \\
=&\text{Var}\begin{bmatrix}\hat{\mathbf{b}}_1\\ \hat{\mathbf{b}}_2\end{bmatrix} - \begin{bmatrix}(\sum_{k=1}^K\sigma_{k1}^2 + \omega_1)\mathbf{R}_1^2 & (\sum_{k=1}^K\sigma_{k12}^2 + \omega_{12})\mathbf{R}_1\mathbf{R}_2\\ (\sum_{k=1}^K\sigma_{k12}^2 + \omega_{12})\mathbf{R}_1\mathbf{R}_2 & (\sum_{k=1}^K\sigma_{k2}^2 + \omega_2)\mathbf{R}_2^2\end{bmatrix} \\
=&\begin{bmatrix}\hat{\mathbf{S}}_1\mathbf{R}_1\hat{\mathbf{S}}_1 & \mathbf{0}\\ \mathbf{0} & \hat{\mathbf{S}}_2\mathbf{R}_2\hat{\mathbf{S}}_2\end{bmatrix}.
\end{aligned}
\tag{7}
$$

Considering the large sample size of GWASs, we can obtain the asymptotic normal distribution in Equation (9).

## 3.5 The XMAP model accounting for sample structure

Here, we derive XMAP under the genetic drift model in Equation (10). To model the population stratification, we assume that the samples from population 1 are constructed by a 50:50 mixture

of sub-population 1$a$ and sub-population 1$b$, and the samples from population 2 are constructed by a 50:50 mixture of sub-population 2$a$ and sub-population 2$b$. We use $F_{ST,1}$ to denote the allele frequency difference between sub-population 1$a$ and sub-population 1$b$ and use $\sigma_{d_1}$ to denote the mean phenotype difference between sub-population 1$a$ and sub-population 1$b$. Similarly, we use $F_{ST,2}$ and $\sigma_{d_2}$ to denote the allele frequency difference and mean phenotype difference between the two sub-populations of population 2, respectively. To account for the sample structures, we consider an extension of model (1) as follows:

$$\begin{aligned}\mathbf{y}_1 &= \mathbf{X}_1\mathbf{b}_1 + \mathbf{X}_1\boldsymbol{\phi}_1 + \mathbf{d}_1 + \mathbf{e}_1,\\ \mathbf{y}_2 &= \mathbf{X}_2\mathbf{b}_2 + \mathbf{X}_2\boldsymbol{\phi}_2 + \mathbf{d}_2 + \mathbf{e}_2,\end{aligned} \tag{8}$$

where $\mathbf{d}_1 \sin \mathbb{R}^{n_1}$ and $\mathbf{d}_2 \sin \mathbb{R}^{n_2}$ are the environmental stratification terms defined as

$$\begin{aligned} d_{1,i} &= \begin{cases} \sigma_{d_1}, & i \in \text{ sub-population } 1a \\ -\sigma_{d_1}, & i \in \text{ sub-population } 1b \end{cases}, \quad i = 1, \cdots n_1 \ , \\ d_{2,i} &= \begin{cases} \sigma_{d_2}, & i \in \text{ sub-population } 2a \\ -\sigma_{d_2}, & i \in \text{ sub-population } 2b \end{cases}, \quad i = 1, \cdots n_2 \ . \end{aligned} \tag{9}$$

We also assume that the $\mathbf{d}_1$ is independent of $\mathbf{e}_1$ and $\mathbf{d}_2$ is independent of $\mathbf{e}_1$, and that $\text{Var}(y_{1i}) = p\sum_{k=1}^{K}\sigma_{k1}^2 + p\omega_1 + \sigma_{d_1}^2 + \sigma_{\mathbf{e}_1}^2 = 1$ and $\text{Var}(y_{2i}) = p\sum_{k=1}^{K}\sigma_{k2}^2 + p\omega_2 + \sigma_{d_2}^2 + \sigma_{\mathbf{e}_2}^2 = 1$. Using the results of bivariate LDSC [1, 2], we have

$$\begin{aligned} \text{Cov}[\hat{\mathbf{b}}_{1j}, \hat{\mathbf{b}}_{1j'}] &= (\sum_{k=1}^{K}\sigma_{k1}^2 + \omega_1)\sum_{l=1}^{p} r_{1jl}r_{1j'l} + \underbrace{(1 + n_1F_{ST,1}(h_1^2F_{ST,1} + \sigma_{d_1}^2))}_{c_1}\hat{s}_{b,1j}\hat{s}_{b,1j'}r_{1jj'} \\ \text{Cov}[\hat{\mathbf{b}}_{2j}, \hat{\mathbf{b}}_{2j'}] &= (\sum_{k=1}^{K}\sigma_{k2}^2 + \omega_2)\sum_{l=1}^{p} r_{2jl}r_{2j'l} + \underbrace{(1 + n_2F_{ST,2}(h_2^2F_{ST,2} + \sigma_{d_2}^2))}_{c_2}\hat{s}_{b,2j}\hat{s}_{b,2j'}r_{2jj'}, \end{aligned} \tag{10}$$

where $h_1^2 = p\sum_{k=1}^{K}\sigma_{k1}^2 + p\omega_1$ and $h_2^2 = p\sum_{k=1}^{K}\sigma_{k2}^2 + p\omega_2$ are heritabilities of $\mathbf{y}_1$ and $\mathbf{y}_2$, respectively. Then, we can update Supplementary Equation (7) as

$$\text{Var}\begin{bmatrix}\boldsymbol{\epsilon}_1\\ \boldsymbol{\epsilon}_2\end{bmatrix} = \begin{bmatrix} c_1\hat{\mathbf{S}}_1\mathbf{R}_1\hat{\mathbf{S}}_1 & \mathbf{0} \\ \mathbf{0} & c_2\hat{\mathbf{S}}_2\mathbf{R}_2\hat{\mathbf{S}}_2\end{bmatrix}. \tag{11}$$

As we can observe, the inflation constants $c_1$ and $c_2$ can be greater than one in the presence of population stratification ($F_{ST,1} \neq 0$ and $F_{ST,2} \neq 0$, respectively).

### 3.6 Derivation of the variational EM algorithm of XMAP

We derive the variational EM algorithm to obtain the estimate of parameters $\boldsymbol{\Sigma}$ and the approximate posterior $q(\boldsymbol{\gamma}, \boldsymbol{\beta}, \boldsymbol{\phi})$. For simplicity of notation, we suppress the pre-estimated parameters $\{\hat{\boldsymbol{\Omega}}, \hat{c}_1, \hat{c}_2\}$ in the following derivation.

The complete-data log-likelihood is given as

$$
\begin{aligned}
&\Pr(\hat{\mathbf{b}}_1, \hat{\mathbf{b}}_2, \boldsymbol{\gamma}, \boldsymbol{\beta}, \boldsymbol{\phi}|\boldsymbol{\Sigma}) \\
=& \log[\Pr(\hat{\mathbf{b}}_1|\boldsymbol{\gamma}, \boldsymbol{\beta}, \boldsymbol{\phi}) \Pr(\hat{\mathbf{b}}_2|\boldsymbol{\gamma}, \boldsymbol{\beta}, \boldsymbol{\phi}) \prod_k^K \Pr(\beta_{1k}, \beta_{2k}) \prod_k^K \Pr(\boldsymbol{\gamma}_k) \Pr(\boldsymbol{\phi}_1, \boldsymbol{\phi}_2)] \\
=& -\frac{1}{2\hat{c}_1}(\hat{\mathbf{b}}_1 - \sum_k^K \mathbf{R}_1\boldsymbol{\gamma}_k\beta_{1k} - \mathbf{R}_1\boldsymbol{\phi}_1)^T(\hat{\mathbf{S}}_1\mathbf{R}_1\hat{\mathbf{S}}_1)^{-1}(\hat{\mathbf{b}}_1 - \sum_k^K \mathbf{R}_1\boldsymbol{\gamma}_k\beta_{1k} - \mathbf{R}_1\boldsymbol{\phi}_1) \\
& -\frac{1}{2\hat{c}_2}(\hat{\mathbf{b}}_2 - \sum_k^K \mathbf{R}_2\boldsymbol{\gamma}_k\beta_{2k} - \mathbf{R}_2\boldsymbol{\phi}_2)^T(\hat{\mathbf{S}}_2\mathbf{R}_2\hat{\mathbf{S}}_2)^{-1}(\hat{\mathbf{b}}_2 - \sum_k^K \mathbf{R}_2\boldsymbol{\gamma}_k\beta_{2k} - \mathbf{R}_2\boldsymbol{\phi}_2) \\
& -\frac{1}{2}\sum_k^K \log(2\pi)^2|\boldsymbol{\Sigma}_k| - \frac{1}{2}\sum_k^K \begin{bmatrix}\beta_{1k} & \beta_{2k}\end{bmatrix} \boldsymbol{\Sigma}_k^{-1} \begin{bmatrix}\beta_{1k} \\ \beta_{2k}\end{bmatrix} + \sum_j^p \sum_k^K \gamma_{kj} \log\frac{1}{p} \\
& -\frac{p}{2}\log|2\pi\hat{\boldsymbol{\Omega}}| - \frac{1}{2}\sum_{j=1}^p \begin{bmatrix}\phi_{1j} & \phi_{2j}\end{bmatrix} \hat{\boldsymbol{\Omega}}^{-1} \begin{bmatrix}\phi_{1j} \\ \phi_{2j}\end{bmatrix} + \text{constant}
\end{aligned}
\tag{12}
$$

where the constant term do not depend on $\mathbf{b}$ and $\boldsymbol{\phi}$. In practice, the LD matrices $\mathbf{R}_1$ and $\mathbf{R}_2$ can be estimated with population-matched reference samples. However, the LD matrices may not be invertible when some SNPs are in perfect LD or the number of individuals in the reference panel is less than $p$. To address this difficulty, we define the XMAP likelihood by discarding the terms that do not depend on $\mathbf{b}$ and $\boldsymbol{\phi}$:

$$
\begin{aligned}
&\mathcal{L}(\boldsymbol{\Sigma}) \\
=& -\frac{1}{2\hat{c}_1}(-2(\sum_k^K \boldsymbol{\gamma}_k\beta_{1k} + \boldsymbol{\phi}_1)^T\hat{\mathbf{S}}_1^{-2}\hat{\mathbf{b}}_1 + (\sum_k^K \boldsymbol{\gamma}_k\beta_{1k} + \boldsymbol{\phi}_1)^T\hat{\mathbf{S}}_1^{-1}\mathbf{R}_1\hat{\mathbf{S}}_1^{-1}(\sum_k^K \boldsymbol{\gamma}_k\beta_{1k} + \boldsymbol{\phi}_1)) \\
& -\frac{1}{2\hat{c}_2}(-2(\sum_k^K \boldsymbol{\gamma}_k\beta_{2k} + \boldsymbol{\phi}_2)^T\hat{\mathbf{S}}_2^{-2}\hat{\mathbf{b}}_2 + (\sum_k^K \boldsymbol{\gamma}_k\beta_{2k} + \boldsymbol{\phi}_2)^T\hat{\mathbf{S}}_2^{-1}\mathbf{R}_2\hat{\mathbf{S}}_2^{-1}(\sum_k^K \boldsymbol{\gamma}_k\beta_{2k} + \boldsymbol{\phi}_2)) \\
& -\frac{1}{2}\sum_k^K \log(2\pi)^2|\boldsymbol{\Sigma}_k| - \frac{1}{2}\sum_k^K \begin{bmatrix}\beta_{1k} & \beta_{2k}\end{bmatrix} \boldsymbol{\Sigma}_k^{-1} \begin{bmatrix}\beta_{1k} \\ \beta_{2k}\end{bmatrix} + \sum_j^p \sum_k^K \boldsymbol{\gamma}_{kj} \log\frac{1}{p} \\
& -\frac{p}{2}\log|2\pi\hat{\boldsymbol{\Omega}}| - \frac{1}{2}\sum_{j=1}^p \begin{bmatrix}\phi_{1j} & \phi_{2j}\end{bmatrix} \hat{\boldsymbol{\Omega}}^{-1} \begin{bmatrix}\phi_{1j} \\ \phi_{2j}\end{bmatrix}.
\end{aligned}
\tag{13}
$$

As we can observe, this definition of likelihood allows our algorithm to handle non-invertible LD matrices because it does not depend on $\mathbf{R}_1^{-1}$ and $\mathbf{R}_2^{-1}$.

**E-step** Based on the mean field assumption for the variational distribution (15), we can derive

the approximated posterior $q(\boldsymbol{\phi})$:

$$\log q\left(\begin{bmatrix}\boldsymbol{\phi}_1\\ \boldsymbol{\phi}_2\end{bmatrix}\right) = \begin{bmatrix}\boldsymbol{\phi}_1 & \boldsymbol{\phi}_2\end{bmatrix} \begin{bmatrix}\frac{1}{\hat{c}_1}\hat{\mathbf{S}}_1^{-2}\hat{\mathbf{b}}_1 - \frac{1}{\hat{c}_1}\hat{\mathbf{S}}_b^{-1}\mathbf{R}_1\hat{\mathbf{S}}_1^{-1}\sum_{k=1}^K \mathbb{E}_{q_k}(\boldsymbol{\gamma}_k\beta_{1k})\\ \frac{1}{\hat{c}_2}\hat{\mathbf{S}}_2^{-2}\hat{\mathbf{b}}_2 - \frac{1}{\hat{c}_2}\hat{\mathbf{S}}_2^{-1}\mathbf{R}_2\hat{\mathbf{S}}_2^{-1}\sum_{k=1}^K \mathbb{E}_{q_k}(\boldsymbol{\gamma}_k\beta_{2k})\end{bmatrix}$$
$$- \frac{1}{2}\begin{bmatrix}\boldsymbol{\phi}_1 & \boldsymbol{\phi}_2\end{bmatrix}\left(\begin{bmatrix}\frac{1}{\hat{c}_1}\hat{\mathbf{S}}_{b,1}^{-1}\mathbf{R}_1\hat{\mathbf{S}}_{b,1}^{-1} & \mathbf{0}\\ \mathbf{0} & \frac{1}{\hat{c}_2}\hat{\mathbf{S}}_2^{-1}\mathbf{R}_2\hat{\mathbf{S}}_2^{-1}\end{bmatrix} + \hat{\boldsymbol{\Omega}}^{-1}\otimes\mathbf{I}_p\right)\begin{bmatrix}\boldsymbol{\phi}_1\\ \boldsymbol{\phi}_2\end{bmatrix} \tag{14}$$
$$+ \text{constant},$$

where $\otimes$ denotes the Kronecker product, and the expectation is taken under the distribution $q(\boldsymbol{\gamma}_k)$ and $q(\beta_{1k}, \beta_{2k}|\boldsymbol{\gamma}_k)$ for $k = 1, .., K$. From the quadratic form of Supplementary Equation (14), we know that $q(\boldsymbol{\phi})$ follows the normal distribution:

$$\begin{bmatrix}\boldsymbol{\phi}_1\\ \boldsymbol{\phi}_2\end{bmatrix} \sim \mathcal{N}(\tilde{\boldsymbol{\nu}}, \tilde{\boldsymbol{\Lambda}}), \tag{15}$$

where

$$\tilde{\boldsymbol{\Lambda}} = \left(\begin{bmatrix}\frac{1}{\hat{c}_1}\hat{\mathbf{S}}_1^{-1}\mathbf{R}_1\hat{\mathbf{S}}_1^{-1} & \mathbf{0}\\ \mathbf{0} & \frac{1}{\hat{c}_2}\hat{\mathbf{S}}_2^{-1}\mathbf{R}_2\hat{\mathbf{S}}_2^{-1}\end{bmatrix} + \hat{\boldsymbol{\Omega}}^{-1}\otimes\mathbf{I}_p\right)^{-1},$$
$$\tilde{\boldsymbol{\nu}} = \tilde{\boldsymbol{\Lambda}}\begin{bmatrix}\frac{1}{\hat{c}_1}\hat{\mathbf{S}}_1^{-2}\hat{\mathbf{b}}_1 - \frac{1}{\hat{c}_1}\hat{\mathbf{S}}_1^{-1}\mathbf{R}_1\hat{\mathbf{S}}_1^{-1}\sum_{k=1}^K \mathbb{E}_{q_k}(\boldsymbol{\gamma}_k\beta_{1k})\\ \frac{1}{\hat{c}_2}\hat{\mathbf{S}}_2^{-2}\hat{\mathbf{b}}_2 - \frac{1}{\hat{c}_2}\hat{\mathbf{S}}_2^{-1}\mathbf{R}_2\hat{\mathbf{S}}_2^{-1}\sum_{k=1}^K \mathbb{E}_{q_k}(\boldsymbol{\gamma}_k\beta_{2k})\end{bmatrix}. \tag{16}$$

Similarly, $\log q(\beta_{1k}, \beta_{2k}|\gamma_{kj} = 1)$ can be obtained as

$$\log q\left(\begin{bmatrix}\beta_{1k}\\ \beta_{2k}\end{bmatrix}|\gamma_{kj} = 1\right) = \begin{bmatrix}\beta_{1k} & \beta_{2k}\end{bmatrix}\begin{bmatrix}\frac{\hat{\mathbf{b}}_{1j}}{\hat{c}_1\hat{s}_{1j}^2} - \frac{1}{\hat{c}_1\hat{s}_{1j}^2}\mathbf{R}_{1j}^T(\sum_{k'\neq 1}^K \mathbb{E}_{q_{k'}}(\boldsymbol{\gamma}_{k'}\beta_{1k'}) + \mathbb{E}_{q_\phi}(\boldsymbol{\phi}_1))\\ \frac{\hat{\mathbf{b}}_{2j}}{\hat{c}_2\hat{s}_{2j}^2} - \frac{1}{\hat{c}_2\hat{s}_{2j}^2}\mathbf{R}_{2j}^T(\sum_{k'\neq 1}^K \mathbb{E}_{q_{k'}}(\boldsymbol{\gamma}_{k'}\beta_{2k'}) + \mathbb{E}_{q_\phi}(\boldsymbol{\phi}_2))\end{bmatrix}$$
$$- \frac{1}{2}\begin{bmatrix}\beta_{1k} & \beta_{2k}\end{bmatrix}\left(\begin{bmatrix}\frac{r_{1jj}}{\hat{c}_1\hat{s}_{1j}^2} & \mathbf{0}\\ \mathbf{0} & \frac{r_{2jj}}{\hat{c}_2\hat{s}_{2j}^2}\end{bmatrix} + \boldsymbol{\Sigma}_k^{-1}\right)\begin{bmatrix}\beta_{1k}\\ \beta_{2k}\end{bmatrix} \tag{17}$$
$$+ \text{constant},$$

where $\mathbf{R}_{1j} = [r_{1j1}, ..., r_{1jp}]^T$ and $\mathbf{R}_{2j} = [r_{2j1}, ..., r_{2jp}]^T$, the expectation $\mathbb{E}_{q_{k'}}$ is taken under the distributions $q(\boldsymbol{\gamma}_{k'})$ and $q(\beta_{1k'}, \beta_{2k'}|\boldsymbol{\gamma}_k)$ for $k' \neq k$, and the expectation $\mathbb{E}_\phi$ is taken under the distribution $q(\boldsymbol{\phi})$. The expression in Supplementary Equation (17) indicates that $q(\beta_{1k}, \beta_{2k}|\gamma_{kj} = 1)$ follows the normal distribution:

$$\begin{bmatrix}\beta_{1k}\\ \beta_{2k}\end{bmatrix}|\gamma_{kj} = 1 \sim \mathcal{N}(\tilde{\boldsymbol{\mu}}_{kj}, \tilde{\boldsymbol{\Sigma}}_{kj}), \tag{18}$$

where

$$\tilde{\boldsymbol{\Sigma}}_{kj} = \begin{bmatrix}\tilde{\sigma}_{kj,1}^2 & \tilde{\sigma}_{kj,12}^2\\ \tilde{\sigma}_{kj,2}^2 & \tilde{\sigma}_{kj,2}^2\end{bmatrix} = \left(\begin{bmatrix}\frac{r_{1jj}}{\hat{c}_1\hat{s}_{b,1j}^2} & \mathbf{0}\\ \mathbf{0} & \frac{r_{2jj}}{\hat{c}_2\hat{s}_{b,2j}^2}\end{bmatrix} + \boldsymbol{\Sigma}_k^{-1}\right)^{-1},$$
$$\tilde{\boldsymbol{\mu}}_{kj} = \begin{bmatrix}\tilde{\mu}_{kj,1}\\ \tilde{\mu}_{kj,2}\end{bmatrix} = \tilde{\boldsymbol{\Sigma}}_{kj}\begin{bmatrix}\frac{\hat{\mathbf{b}}_{1j}}{\hat{c}_1\hat{s}_{1j}^2} - \frac{1}{\hat{c}_1\hat{s}_{1j}^2}\mathbf{R}_{1j}^T(\sum_{k'\neq 1}^K \mathbb{E}_{q_{k'}}(\boldsymbol{\gamma}_{k'}\beta_{1k'}) + \mathbb{E}_{q_\phi}(\boldsymbol{\phi}_1))\\ \frac{\hat{\mathbf{b}}_{2j}}{\hat{c}_2\hat{s}_{2j}^2} - \frac{1}{\hat{c}_2\hat{s}_{2j}^2}\mathbf{R}_{2j}^T(\sum_{k'\neq 1}^K \mathbb{E}_{q_{k'}}(\boldsymbol{\gamma}_{k'}\beta_{2k'}) + \mathbb{E}_{q_\phi}(\boldsymbol{\phi}_2))\end{bmatrix}. \tag{19}$$

Let $q(\boldsymbol{\gamma}_k) = \tilde{\boldsymbol{\pi}} = [\tilde{\pi}_{k1}, ..., \tilde{\pi}_{kp}]^T$, where $\sum_{j=1}^{p} \tilde{\pi}_{kj} = 1$ for $k = 1, .., K$. We can obtain $q(\boldsymbol{\gamma}, \boldsymbol{\beta}, \boldsymbol{\phi})$ as

$$q(\boldsymbol{\gamma}, \boldsymbol{\beta}, \boldsymbol{\phi}) = \prod_{k}^{K} q(\boldsymbol{\gamma}_k) q(\beta_{1k}, \beta_{2k}|\boldsymbol{\gamma}_k) q(\boldsymbol{\phi}_1, \boldsymbol{\phi}_2) = \prod_{k}^{K}\prod_{j}^{p} \Big[\tilde{\boldsymbol{\pi}}_{kj}\mathcal{N}(\tilde{\boldsymbol{\mu}}_{kj}, \tilde{\boldsymbol{\Sigma}}_{kj})\Big]^{\gamma_{kj}} \mathcal{N}(\tilde{\boldsymbol{\nu}}, \tilde{\boldsymbol{\Lambda}}). \quad (20)$$

Then, we can evaluate the lower bound given by Equation (13)

$$\begin{aligned}
&\mathcal{L}_q(\boldsymbol{\Sigma}) = \mathbb{E}_q[\mathcal{L}(\boldsymbol{\Sigma})] - \mathbb{E}_q[q(\boldsymbol{\gamma}, \boldsymbol{\beta}, \boldsymbol{\phi}))] \\
&= \left(\sum_{k}^{K} \tilde{\boldsymbol{\mu}}_{kj} \otimes \tilde{\boldsymbol{\pi}}_k + \tilde{\boldsymbol{\nu}}\right)^T \begin{bmatrix} \frac{\hat{\mathbf{S}}_1^{-2}\hat{\mathbf{b}}_1}{\hat{c}_1} \\ \frac{\hat{\mathbf{S}}_2^{-2}\hat{\mathbf{b}}_2}{\hat{c}_2} \end{bmatrix} - \frac{1}{2}\left(\sum_{k}^{K} \tilde{\boldsymbol{\mu}}_{kj} \otimes \tilde{\boldsymbol{\pi}}_k + \tilde{\boldsymbol{\nu}}\right)^T \begin{bmatrix} \frac{\hat{\mathbf{S}}_1^{-1}\mathbf{R}_1\hat{\mathbf{S}}_1^{-1}}{\hat{c}_1} & \mathbf{0} \\ \mathbf{0} & \frac{\hat{\mathbf{S}}_2^{-1}\mathbf{R}_2\hat{\mathbf{S}}_2^{-1}}{\hat{c}_2} \end{bmatrix} \left(\sum_{k}^{K} \tilde{\boldsymbol{\mu}}_{kj} \otimes \tilde{\boldsymbol{\pi}}_k + \tilde{\boldsymbol{\nu}}\right) \\
&\quad - \sum_{j}^{p} \frac{1}{2\hat{c}_1\hat{s}_{1j}^2} r_{1jj} \sum_{k}^{K} \tilde{\pi}_{kj}(\tilde{\mu}_{kj,1}^2 + \tilde{\sigma}_{kj,1}^2) - \sum_{j}^{p} \frac{1}{2\hat{c}_2\hat{s}_{2j}^2} r_{2jj} \sum_{k}^{K} \tilde{\pi}_{kj}(\tilde{\mu}_{kj,2}^2 + \tilde{\sigma}_{kj,2}^2) \\
&\quad + \frac{1}{2}\sum_{k}^{K}\left( (\tilde{\boldsymbol{\mu}}_{kj} \otimes \tilde{\boldsymbol{\pi}}_k)^T \begin{bmatrix} \frac{\hat{\mathbf{S}}_1^{-1}\mathbf{R}_1\hat{\mathbf{S}}_1^{-1}}{\hat{c}_1} & \mathbf{0} \\ \mathbf{0} & \frac{\hat{\mathbf{S}}_2^{-1}\mathbf{R}_2\hat{\mathbf{S}}_2^{-1}}{\hat{c}_2} \end{bmatrix} (\tilde{\boldsymbol{\mu}}_{kj} \otimes \tilde{\boldsymbol{\pi}}_k)\right) \\
&\quad - \frac{1}{2}\sum_{k}^{K} \log(2\pi)^2|\boldsymbol{\Sigma}_k| - \frac{1}{2}\sum_{k}\sum_{j} \tilde{\pi}_{kj} \mathrm{Tr}(\boldsymbol{\Sigma}_k^{-1}(\tilde{\boldsymbol{\Sigma}}_{kj} + \tilde{\boldsymbol{\mu}}_{kj}\tilde{\boldsymbol{\mu}}_{kj}^T)) + \sum_{j}^{p}\sum_{k}^{K} \tilde{\pi}_{kj} \log\frac{1}{p} \\
&\quad - \frac{p}{2}\log|2\pi\hat{\boldsymbol{\Omega}}| - \frac{1}{2}\tilde{\boldsymbol{\nu}}^T(\hat{\boldsymbol{\Omega}}^{-1} \otimes \mathbf{I}_p)\tilde{\boldsymbol{\nu}} - \frac{1}{2}\mathrm{Tr}\left(\left(\begin{bmatrix} \frac{1}{\hat{c}_1}\hat{\mathbf{S}}_1^{-1}\mathbf{R}_1\hat{\mathbf{S}}_1^{-1} & \mathbf{0} \\ \mathbf{0} & \frac{1}{\hat{c}_2}\hat{\mathbf{S}}_2^{-1}\mathbf{R}_2\hat{\mathbf{S}}_2^{-1} \end{bmatrix} + \hat{\boldsymbol{\Omega}}^{-1} \otimes \mathbf{I}_p\right)\tilde{\boldsymbol{\Lambda}}\right) \\
&\quad - \sum_{j}^{p}\sum_{k}^{K} \tilde{\pi}_{kj}\log\tilde{\pi}_{kj} + \frac{1}{2}\sum_{j}^{p}\sum_{k}^{K} \tilde{\pi}_{kj}\log|\tilde{\boldsymbol{\Sigma}}_{kj}| + K + K\log(2\pi) + \frac{1}{2}\log|\tilde{\boldsymbol{\Lambda}}| + p + p\log 2\pi \\
&= \left(\sum_{k}^{K} \tilde{\boldsymbol{\mu}}_{kj} \otimes \tilde{\boldsymbol{\pi}}_k + \tilde{\boldsymbol{\nu}}\right)^T \begin{bmatrix} \frac{\hat{\mathbf{S}}_1^{-2}\hat{\mathbf{b}}_1}{\hat{c}_1} \\ \frac{\hat{\mathbf{S}}_2^{-2}\hat{\mathbf{b}}_2}{\hat{c}_2} \end{bmatrix} - \frac{1}{2}\left(\sum_{k}^{K} \tilde{\boldsymbol{\mu}}_{kj} \otimes \tilde{\boldsymbol{\pi}}_k + \tilde{\boldsymbol{\nu}}\right)^T \begin{bmatrix} \frac{\hat{\mathbf{S}}_1^{-1}\mathbf{R}_1\hat{\mathbf{S}}_1^{-1}}{\hat{c}_1} & \mathbf{0} \\ \mathbf{0} & \frac{\hat{\mathbf{S}}_2^{-1}\mathbf{R}_2\hat{\mathbf{S}}_2^{-1}}{\hat{c}_2} \end{bmatrix} \left(\sum_{k}^{K} \tilde{\boldsymbol{\mu}}_{kj} \otimes \tilde{\boldsymbol{\pi}}_k + \tilde{\boldsymbol{\nu}}\right) \\
&\quad - \sum_{j}^{p} \frac{1}{2\hat{c}_1\hat{s}_{1j}^2} r_{1jj} \sum_{k}^{K} \tilde{\pi}_{kj}(\tilde{\mu}_{kj,1}^2 + \tilde{\sigma}_{kj,1}^2) - \sum_{j}^{p} \frac{1}{2\hat{c}_2\hat{s}_{2j}^2} r_{2jj} \sum_{k}^{K} \tilde{\pi}_{kj}(\tilde{\mu}_{kj,2}^2 + \tilde{\sigma}_{kj,2}^2) \\
&\quad + \frac{1}{2}\sum_{k}^{K}\left( (\tilde{\boldsymbol{\mu}}_{kj} \otimes \tilde{\boldsymbol{\pi}}_k)^T \begin{bmatrix} \frac{\hat{\mathbf{S}}_1^{-1}\mathbf{R}_1\hat{\mathbf{S}}_1^{-1}}{\hat{c}_1} & \mathbf{0} \\ \mathbf{0} & \frac{\hat{\mathbf{S}}_2^{-1}\mathbf{R}_2\hat{\mathbf{S}}_2^{-1}}{\hat{c}_2} \end{bmatrix} (\tilde{\boldsymbol{\mu}}_{kj} \otimes \tilde{\boldsymbol{\pi}}_k)\right) \\
&\quad - \frac{1}{2}\sum_{k}\sum_{j} \boldsymbol{\gamma}_{kj} \mathrm{Tr}(\boldsymbol{\Sigma}_k^{-1}(\tilde{\boldsymbol{\Sigma}}_{kj} + \tilde{\boldsymbol{\mu}}_{kj}\tilde{\boldsymbol{\mu}}_{kj}^T)) \\
&\quad - \frac{p}{2}\log|2\pi\hat{\boldsymbol{\Omega}}| - \frac{1}{2}\tilde{\boldsymbol{\nu}}^T(\hat{\boldsymbol{\Omega}}^{-1} \otimes \mathbf{I}_p)\tilde{\boldsymbol{\nu}} - \frac{1}{2}\mathrm{Tr}\left(\left(\begin{bmatrix} \frac{1}{\hat{c}_1}\hat{\mathbf{S}}_1^{-1}\mathbf{R}_1\hat{\mathbf{S}}_1^{-1} & \mathbf{0} \\ \mathbf{0} & \frac{1}{\hat{c}_2}\hat{\mathbf{S}}_2^{-1}\mathbf{R}_2\hat{\mathbf{S}}_2^{-1} \end{bmatrix} + \hat{\boldsymbol{\Omega}}^{-1} \otimes \mathbf{I}_p\right)\tilde{\boldsymbol{\Lambda}}\right) \\
&\quad + \sum_{j}^{p}\sum_{k}^{K} \tilde{\pi}_{kj}\log\frac{1}{p} - \sum_{j}^{p}\sum_{k}^{K} \tilde{\pi}_{kj}\log\tilde{\pi}_{kj} + \frac{1}{2}\sum_{j}^{p}\sum_{k}^{K} [\tilde{\pi}_{kj}(\log|\tilde{\boldsymbol{\Sigma}}_{kj}| - \log|\boldsymbol{\Sigma}_k|)] + \frac{1}{2}\log|\tilde{\boldsymbol{\Lambda}}| \\
&\quad + \text{constant}.
\end{aligned} \quad (21)$$

By setting the derivative of the lower bound w.r.t $\tilde{\pi}_{kj}$ as zero, we can get the update of variational parameter $\gamma_{kj}$:

$$\tilde{\pi}_{kj} = softmax(\log\frac{1}{p} + \frac{1}{2}\log|\tilde{\mathbf{\Sigma}}_{kj}| + \frac{1}{2}\tilde{\boldsymbol{\mu}}_{kj}^T\tilde{\mathbf{\Sigma}}_{kj}^{-1}\tilde{\boldsymbol{\mu}}_{kj}), \tag{22}$$

where $softmax$ is the softmax function to make sure $\sum_{j=1}^{p}\tilde{\pi}_{kj} = 1$. Combining, we can obtain the updating equations of the variational parameters in Equation (16).

**M-step** At M-step, we set $\frac{\partial\mathcal{L}_q}{\partial\mathbf{\Sigma}_k} = 0$ to obtain the update equation of $\mathbf{\Sigma}_k$:

$$\mathbf{\Sigma}_k = \sum_{j}^{p}\tilde{\pi}_{kj}(\tilde{\boldsymbol{\mu}}_{kj}\tilde{\boldsymbol{\mu}}_{kj}^T + \tilde{\mathbf{\Sigma}}_{kj}). \tag{23}$$

## 3.7 Adjustment to $z$-scores

In the derivation of XMAP, we have made the approximations

$$\begin{aligned} \hat{s}_{1j} &= \sqrt{||\mathbf{y}_1 - \mathbf{x}_{1j}\hat{b}_{1j}||_2^2/(n_1\mathbf{x}_{1j}^T\mathbf{x}_{1j})} \approx \frac{1}{\sqrt{n_1}}, \\ \hat{s}_{2j} &= \sqrt{||\mathbf{y}_2 - \mathbf{x}_{2j}\hat{b}_{2j}||_2^2/(n_2\mathbf{x}_{2j}^T\mathbf{x}_{2j})} \approx \frac{1}{\sqrt{n_2}}. \end{aligned} \tag{24}$$

These approximations implicitly assume that the genetic effect contributed by a single variant is ignorable. This assumption makes the results of summary-level method different from the individual-level method. However, in the special case when the confounding bias is absent, we can derive a summary-level XMAP likelihood that exactly reproduce the individual-level results with in-sample LD. To see this, we first denote the $z$-scores $\{\hat{\mathbf{z}}_1\}_j = \hat{z}_{1j} = \frac{\hat{b}_{1j}}{\hat{s}_{1j}}$ and $\{\hat{\mathbf{z}}_2\}_j = \hat{z}_{2j} = \frac{\hat{b}_{2j}}{\hat{s}_{2j}}$. We can re-write the XMAP data likelihood in Equation (9) in the main text as distributions of $z$-scores

$$\begin{aligned} \hat{\mathbf{z}}_1 &\sim \mathcal{N}(\sqrt{n_1}\mathbf{R}_1(\mathbf{b}_1 + \boldsymbol{\phi}_1), \mathbf{R}_1), \\ \hat{\mathbf{z}}_2 &\sim \mathcal{N}(\sqrt{n_2}\mathbf{R}_2(\mathbf{b}_2 + \boldsymbol{\phi}_2), \mathbf{R}_2). \end{aligned} \tag{25}$$

The above model can be modified to remove the assumption of ignorable genetic effect contributed by a single variant. Specifically, we apply the following adjustment to the $z$-scores:

$$\begin{aligned} \tilde{\mathbf{z}}_1 &:= \mathbf{D}_1^{-1/2}\hat{\mathbf{z}}_1 = \frac{\mathbf{X}_1^T\mathbf{y}_1}{\sqrt{n_1}}, \\ \tilde{\mathbf{z}}_2 &:= \mathbf{D}_2^{-1/2}\hat{\mathbf{z}}_2 = \frac{\mathbf{X}_2^T\mathbf{y}_2}{\sqrt{n_2}}, \end{aligned} \tag{26}$$

where $\mathbf{D}_1 \in \mathbb{R}^{p\times p}$ and $\mathbf{D}_2 \in \mathbb{R}^{p\times p}$ are diagonal matrices with $j$-th diagonal elements being $\frac{n_1}{n_1+\hat{z}_{1j}^2}$ and $\frac{n_2}{n_2+\hat{z}_{2j}^2}$, respectively. These are the maximum likelihood estimates of the residual variance expressed with $z$-scores. By replacing the $\hat{\mathbf{z}}_1$ and $\hat{\mathbf{z}}_2$ in Supplementary Equation (25) with $\tilde{\mathbf{z}}_1$ and $\tilde{\mathbf{z}}_2$, respectively, we have

$$\begin{aligned} \tilde{\mathbf{z}}_1 &\sim \mathcal{N}(\sqrt{n_1}\mathbf{R}_1(\mathbf{b}_1 + \boldsymbol{\phi}_1), \mathbf{R}_1), \\ \tilde{\mathbf{z}}_2 &\sim \mathcal{N}(\sqrt{n_2}\mathbf{R}_2(\mathbf{b}_2 + \boldsymbol{\phi}_2), \mathbf{R}_2). \end{aligned} \tag{27}$$

Next, we show that model (27) is equivalent to the individual-model model. With Equation (1) in the main text, the individual-level XMAP model can be written as

$$\begin{aligned}\mathbf{y}_1 &\sim \mathcal{N}(\mathbf{X}_1(\mathbf{b}_1+\boldsymbol{\phi}_1), \sigma^2_{\mathbf{e}_1}\mathbf{I}_{n_1}),\\ \mathbf{y}_2 &\sim \mathcal{N}(\mathbf{X}_2(\mathbf{b}_2+\boldsymbol{\phi}_2), \sigma^2_{\mathbf{e}_2}\mathbf{I}_{n_2}).\end{aligned} \tag{28}$$

By assuming that the phenotype vectors are standardized to have a mean of zero and unit variance and fixing $\sigma^2_{\mathbf{e}_1} = \mathbf{y}_1^T\mathbf{y}_1/n_1 = 1$ and $\sigma^2_{\mathbf{e}_2} = \mathbf{y}_2^T\mathbf{y}_2/n_2 = 1$, we have

$$\begin{aligned}\frac{\mathbf{X}_1^T\mathbf{y}_1}{\sqrt{n_1}} &\sim \mathcal{N}(\frac{\mathbf{X}_1^T\mathbf{X}_1}{n_1}\times\sqrt{n_1}(\mathbf{b}_1+\boldsymbol{\phi}_1), \frac{\mathbf{X}_1^T\mathbf{X}_1}{n_1}),\\ \frac{\mathbf{X}_2^T\mathbf{y}_2}{\sqrt{n_2}} &\sim \mathcal{N}(\frac{\mathbf{X}_2^T\mathbf{X}_2}{n_2}\times\sqrt{n_2}(\mathbf{b}_2+\boldsymbol{\phi}_2), \frac{\mathbf{X}_2^T\mathbf{X}_2}{n_2}).\end{aligned} \tag{29}$$

Clearly, with the substitutions $\tilde{\mathbf{z}}_1 = \frac{\mathbf{X}_1^T\mathbf{y}_1}{\sqrt{n_1}}$, $\tilde{\mathbf{z}}_2 = \frac{\mathbf{X}_2^T\mathbf{y}_2}{\sqrt{n_2}}$, $\mathbf{R}_1 = \frac{\mathbf{X}_1^T\mathbf{X}_1}{n_1}$, and $\mathbf{R}_2 = \frac{\mathbf{X}_2^T\mathbf{X}_2}{n_2}$, model (29) is equivalent to model (27). Therefore, by applying the adjustment in Supplementary Equation (26), XMAP can produce exactly the same results as the individual-level model. We implement this adjustment in the updated version of XMAP software and provide an option to make such an adjustment.

# Supplementary References

[1] Brendan K Bulik-Sullivan, Po-Ru Loh, Hilary K Finucane, Stephan Ripke, Jian Yang, Nick Patterson, Mark J Daly, Alkes L Price, and Benjamin M Neale. LD score regression distinguishes confounding from polygenicity in genome-wide association studies. *Nature genetics*, 47(3):291–295, 2015.

[2] Zheng Ning, Yudi Pawitan, and Xia Shen. High-definition likelihood inference of genetic correlations across human complex traits. *Nature genetics*, 52(8):859–864, 2020.
