# Supplementary material for: XMAP: Cross-population fine-mapping by leveraging genetic diversity and accounting for confounding bias
Source: Nat Commun. 2023 Oct 28;14:6870. doi: 10.1038/s41467-023-42614-7 (PMC10613261; doi:10.1038/s41467-023-42614-7)
Supplement: Supplementary file 3 — Description of Additional Supplementary Files [file 41467_2023_42614_MOESM3_ESM.pdf]

File name: Supplementary Software 1

Description: The source code of R package XMAP is attached in the zip file XMAP_1.0.tar.gz. Alternatively, the software can be installed from our GitHub repo: https://github.com/YangLabHKUST/XMAP. Codes for reproducing the real data results in Figures 4-6 are attached in the reproduce folder. Complete codes for reproducing manuscript results can be found at https://github.com/YangLabHKUST/XMAP/tree/main/results.
