# Supplementary material for: XMAP: Cross-population fine-mapping by leveraging genetic diversity and accounting for confounding bias
Source: Nat Commun. 2023 Oct 28;14:6870. doi: 10.1038/s41467-023-42614-7 (PMC10613261; doi:10.1038/s41467-023-42614-7)
Supplement: Supplementary file 4 — Supplementary Software 1 [file 41467_2023_42614_MOESM4_ESM.zip › Software_submit_r3/reproduce/Fig5_replicate_r1.html]

Fig5\_replicate\_r1


# Figure 5 height replication study¶

This notebook reproduces the Figure 5 A-D in XMAP manuscript.

GWAS summary statistics can be found at https://hkustconnect-my.sharepoint.com/:f:/g/personal/mcaiad\_connect\_ust\_hk/EumV7yII87BAsaA-SuYw3ZcBo0Xa1OYbDy6T9hHeq51UJA?e=ulWzqq

PIP files required for running this script can be found at https://hkustconnect-my.sharepoint.com/:f:/g/personal/mcaiad\_connect\_ust\_hk/Eptgw2McNLRKjsypetdRwzABaA\_JM20jEeGgSwwc\_GfK1g?e=XNloZD

In [2]:

```
library(ggplot2)
library(data.table)
library(dplyr)
```

```
Attaching package: ‘dplyr’


The following objects are masked from ‘package:data.table’:

    between, first, last


The following objects are masked from ‘package:stats’:

    filter, lag


The following objects are masked from ‘package:base’:

    intersect, setdiff, setequal, union
```

## Figure 5 A-B: Replication in Sibship GWAS¶

In [5]:

```
thr_pip_all <- c(0.8, 0.9)
K <- 10

pip_sibship <- readRDS("/home/share/mingxuan/fine_mapping/analysis/results/height_sibship_pip_allSNPs.RDS")
sumstat_EUR <- fread("/home/share/sumstats/format/height_EUR_sibship_summary_format.txt")
pip_susiex <- fread(paste0("/home/share/mingxuan/fine_mapping/analysis/results/height_susiex_success_UKB_WG_K", K, "_PIP.txt"))
pip_susiex$PIP[is.na(pip_susiex$PIP)] <- 0
snps <- intersect(intersect(pip_sibship$rsid, sumstat_EUR$SNP), pip_susiex$rsid)
pip_sibship <- pip_sibship[match(snps, pip_sibship$rsid),]
sumstat_EUR <- sumstat_EUR[match(snps, sumstat_EUR$SNP),]
pval <- 2 * pnorm(abs(sumstat_EUR$Z), lower.tail = F)

out <- data.frame()
p_all <- vector("list", 3)
names(p_all) <- thr_pip_all

pip_xmap <- fread(paste0("/home/share/mingxuan/fine_mapping/analysis/results/height_UKB_WG_K",K,"_PIP.txt"))
pip_eas <- fread(paste0("/home/share/mingxuan/fine_mapping/analysis/results/height_WG_K",K,"_PIP.txt"))
pip_eur <- fread(paste0("/home/share/mingxuan/fine_mapping/analysis/results/height_UKB_K",K,"_PIP.txt"))
pip_max <- data.frame(pip_eas[,1:5],PIP=apply(cbind(pip_eas$PIP,pip_eur$PIP),1,max))
pip_inf_eur <- fread(paste0("/import/home/share/mingxuan/fine_mapping/analysis/results/height_susieinf_converge_UKB_K", K, "_PIP.txt"))
pip_inf_eas <- fread(paste0("/import/home/share/mingxuan/fine_mapping/analysis/results/height_susieinf_converge_WG_K", K, "_PIP.txt"))

pip_inf_eur$PIP[!pip_inf_eur$converge] <- 0
pip_inf_eas$PIP[!pip_inf_eas$converge] <- 0
```

In [6]:

```
out <- data.frame()
p_all <- vector("list", 3)
names(p_all) <- thr_pip_all


for (thr_pip in thr_pip_all) {

  xmap <- pip_xmap[pip_xmap$PIP>thr_pip&pip_xmap$rsid%in%sumstat_EUR$SNP,]
  xmap$pval <- pval[match(xmap$rsid, sumstat_EUR$SNP)]
  xmap$pip_rep <- pip_sibship$PIP[match(xmap$rsid, pip_sibship$rsid)]

  eur <- pip_eur[pip_eur$PIP>thr_pip&pip_eur$rsid%in%sumstat_EUR$SNP,]
  eur$pval <- pval[match(eur$rsid, sumstat_EUR$SNP)]
  eur$pip_rep <- pip_sibship$PIP[match(eur$rsid, pip_sibship$rsid)]

  eas <- pip_eas[pip_eas$PIP>thr_pip&pip_eas$rsid%in%sumstat_EUR$SNP,]
  eas$pval <- pval[match(eas$rsid, sumstat_EUR$SNP)]
  eas$pip_rep <- pip_sibship$PIP[match(eas$rsid, pip_sibship$rsid)]
    
  susie_max <- pip_max[pip_max$PIP > thr_pip & pip_max$rsid %in% sumstat_EUR$SNP,]
  susie_max$pval <- pval[match(susie_max$rsid, sumstat_EUR$SNP)]
  susie_max$pip_rep <- pip_sibship$PIP[match(susie_max$rsid, pip_sibship$rsid)]

  susiex <- pip_susiex[pip_susiex$PIP > thr_pip & pip_susiex$rsid %in% sumstat_EUR$SNP,]
  susiex$pval <- pval[match(susiex$rsid, sumstat_EUR$SNP)]
  susiex$pip_rep <- pip_sibship$PIP[match(susiex$rsid, pip_sibship$rsid)]

  inf_eur <- pip_inf_eur[pip_inf_eur$PIP > thr_pip & pip_inf_eur$rsid %in% sumstat_EUR$SNP,1:6]
  inf_eur$pval <- pval[match(inf_eur$rsid, sumstat_EUR$SNP)]
  inf_eur$pip_rep <- pip_sibship$PIP[match(inf_eur$rsid, pip_sibship$rsid)]

  inf_eas <- pip_inf_eas[pip_inf_eas$PIP > thr_pip & pip_inf_eas$rsid %in% sumstat_EUR$SNP,1:6]
  inf_eas$pval <- pval[match(inf_eas$rsid, sumstat_EUR$SNP)]
  inf_eas$pip_rep <- pip_sibship$PIP[match(inf_eas$rsid, pip_sibship$rsid)]

  p_all[[paste(thr_pip)]] <- list(EUR = eur$pval,
                                  EAS = eas$pval,
                                  SuSiE_max = susie_max$pval,
                                  SuSiEInf_EUR = inf_eur$pval,
                                  SuSiEInf_EAS = inf_eas$pval,
                                  XMAP = xmap$pval,
                                  SuSiEx = susiex$pval)

  out <- rbind(out,
               data.frame(xmap, method = "XMAP", thr_pip = thr_pip),
               data.frame(eur, method = "SuSiE-EUR", thr_pip = thr_pip),
               data.frame(eas, method = "SuSiE-EAS", thr_pip = thr_pip),
               data.frame(susie_max, method = "SuSiE-merge", thr_pip = thr_pip),
               data.frame(inf_eur, method = "SuSiE-inf-EUR", thr_pip = thr_pip),
               data.frame(inf_eas, method = "SuSiE-inf-EAS", thr_pip = thr_pip),
               data.frame(susiex, method = "SuSiEx", thr_pip = thr_pip))
}

out$sig <- factor(ifelse(out$pval < 5e-8, "< 5e-8", "> 5e-8"),
                  levels = c("> 5e-8", "< 5e-8"))
out$piprange <- ifelse(out$pip_rep > 0.1, ">0.1", "<0.1")
out$piprange[out$pip_rep > 0.1 & out$pip_rep < 0.5] <- "0.1-0.5"
out$piprange[out$pip_rep > 0.5 & out$pip_rep < 0.9] <- "0.5-0.9"
out$piprange[out$pip_rep > 0.9] <- ">0.9"
out$piprange <- factor(out$piprange, levels = c("<0.1", "0.1-0.5", "0.5-0.9", ">0.9"))
out$thr_pip <- paste0("PIP threshold=",out$thr_pip)
out$method <- factor(out$method,levels=c("XMAP", "SuSiEx", "SuSiE-inf-EUR", "SuSiE-inf-EAS", "SuSiE-merge", "SuSiE-EUR", "SuSiE-EAS"))
```

### Figure 5A: Replication rate evaluated with GWAS p-value in Sibship¶

In [8]:

```
options(repr.plot.width=18, repr.plot.height=3)
ggplot(out, aes(x = method, fill = sig)) +
  geom_bar(position = position_fill()) +
  geom_text(stat = 'count', aes(label = ..count..), position = position_fill(vjust = 0.5)) +
  scale_y_continuous(labels = scales::percent) +
  ylab("Number of SNPs") +
  facet_grid(.~thr_pip) +
  scale_fill_brewer(palette = "Paired") +
  theme_minimal() +
  coord_flip() +
  guides(fill=guide_legend(title="p-value in sibship GWAS")) +
  theme(legend.position = "bottom")
```

### Figure 5B: Replication rate evaluated with SuSiE PIP in Sibship¶

In [9]:

```
ggplot(out, aes(x = method, fill = piprange)) +
  geom_bar(position = position_fill()) +
  geom_text(stat = 'count', aes(label = ..count..), position = position_fill(vjust = 0.5)) +
  scale_y_continuous(labels = scales::percent) +
  ylab("Percentage") +
  facet_grid(.~thr_pip) +
  scale_fill_brewer(palette = "Paired") +
  theme_minimal() +
  coord_flip() +
  guides(fill=guide_legend(title="PIP in sibship GWAS")) +
  theme(legend.position = "bottom")
```

## Figure 5 A-B: Replication in BBJ GWAS¶

Because BBj GWAS is affected by population stratification (LDSC intercept=1.39, s.e.=0.024), we first corrected the inflation using the MTAG by setting gc=0, then used the correted p-value for replication. For BBJ PIP, we used the officially released PIP computed by SuSiE with in-sample LD, reducing false signals.

In [10]:

```
thr_pip_all <- c(0.8, 0.9)

K <- 10

pip_bbj <- fread("/import/home/share/mingxuan/fine_mapping/analysis/hum0197/BBJ.Height.Kanai2021.SuSiE.tsv.gz")
pip_bbj$PIP <- pip_bbj$pip
sumstat_EAS <- fread("/home/share/mingxuan/fine_mapping/analysis/mtag/height_bbj_ukbNeale_0gc_trait_1.txt")
pip_susiex <- fread(paste0("/home/share/mingxuan/fine_mapping/analysis/results/height_susiex_success_UKB_WG_K", K, "_PIP.txt"))
pip_susiex$PIP[is.na(pip_susiex$PIP)] <- 0
snps <- intersect(intersect(pip_bbj$rsid, sumstat_EAS$SNP), pip_susiex$rsid)
pip_bbj <- pip_bbj[match(snps, pip_bbj$rsid),]
sumstat_EAS <- sumstat_EAS[match(snps, sumstat_EAS$SNP),]
pval <- 2 * pnorm(abs(sumstat_EAS$mtag_z), lower.tail = F)
pval[pval < 1e-50] <- 1e-50


pip_xmap <- fread(paste0("/home/share/mingxuan/fine_mapping/analysis/results/height_UKB_WG_K",K,"_PIP.txt"))
pip_eas <- fread(paste0("/home/share/mingxuan/fine_mapping/analysis/results/height_WG_K",K,"_PIP.txt"))
pip_eur <- fread(paste0("/home/share/mingxuan/fine_mapping/analysis/results/height_UKB_K",K,"_PIP.txt"))

pip_max <- data.frame(pip_eas[,1:5],PIP=apply(cbind(pip_eas$PIP,pip_eur$PIP),1,max))

pip_inf_eur <- fread(paste0("/import/home/share/mingxuan/fine_mapping/analysis/results/height_susieinf_UKB_K", K, "_PIP.txt"))
pip_inf_eas <- fread(paste0("/import/home/share/mingxuan/fine_mapping/analysis/results/height_susieinf_WG_K", K, "_PIP.txt"))
```

In [11]:

```
out <- data.frame()
p_all <- vector("list", 3)
names(p_all) <- thr_pip_all


for (thr_pip in thr_pip_all) {

  xmap <- pip_xmap[pip_xmap$PIP>thr_pip&pip_xmap$rsid%in%sumstat_EAS$SNP,]
  xmap$pval <- pval[match(xmap$rsid, sumstat_EAS$SNP)]
  xmap$pip_rep <- pip_bbj$PIP[match(xmap$rsid, pip_bbj$rsid)]

  eur <- pip_eur[pip_eur$PIP>thr_pip&pip_eur$rsid%in%sumstat_EAS$SNP,]
  eur$pval <- pval[match(eur$rsid, sumstat_EAS$SNP)]
  eur$pip_rep <- pip_bbj$PIP[match(eur$rsid, pip_bbj$rsid)]

  eas <- pip_eas[pip_eas$PIP>thr_pip&pip_eas$rsid%in%sumstat_EAS$SNP,]
  eas$pval <- pval[match(eas$rsid, sumstat_EAS$SNP)]
  eas$pip_rep <- pip_bbj$PIP[match(eas$rsid, pip_bbj$rsid)]
    
  susie_max <- pip_max[pip_max$PIP > thr_pip & pip_max$rsid %in% sumstat_EAS$SNP,]
  susie_max$pval <- pval[match(susie_max$rsid, sumstat_EAS$SNP)]
  susie_max$pip_rep <- pip_bbj$PIP[match(susie_max$rsid, pip_bbj$rsid)]

  susiex <- pip_susiex[pip_susiex$PIP > thr_pip & pip_susiex$rsid %in% sumstat_EAS$SNP,]
  susiex$pval <- pval[match(susiex$rsid, sumstat_EAS$SNP)]
  susiex$pip_rep <- pip_bbj$PIP[match(susiex$rsid, pip_bbj$rsid)]

  inf_eur <- pip_inf_eur[pip_inf_eur$PIP > thr_pip & pip_inf_eur$rsid %in% sumstat_EAS$SNP,]
  inf_eur$pval <- pval[match(inf_eur$rsid, sumstat_EAS$SNP)]
  inf_eur$pip_rep <- pip_bbj$PIP[match(inf_eur$rsid, pip_bbj$rsid)]

  inf_eas <- pip_inf_eas[pip_inf_eas$PIP > thr_pip & pip_inf_eas$rsid %in% sumstat_EAS$SNP,]
  inf_eas$pval <- pval[match(inf_eas$rsid, sumstat_EAS$SNP)]
  inf_eas$pip_rep <- pip_bbj$PIP[match(inf_eas$rsid, pip_bbj$rsid)]

  p_all[[paste(thr_pip)]] <- list(EUR = eur$pval,
                                  EAS = eas$pval,
                                  SuSiE_max = susie_max$pval,
                                  SuSiEInf_EUR = inf_eur$pval,
                                  SuSiEInf_EAS = inf_eas$pval,
                                  XMAP = xmap$pval,
                                  SuSiEx = susiex$pval)

  out <- rbind(out,
               data.frame(xmap, method = "XMAP", thr_pip = thr_pip),
               data.frame(eur, method = "SuSiE-EUR", thr_pip = thr_pip),
               data.frame(eas, method = "SuSiE-EAS", thr_pip = thr_pip),
               data.frame(susie_max, method = "SuSiE-merge", thr_pip = thr_pip),
               data.frame(inf_eur, method = "SuSiE-inf-EUR", thr_pip = thr_pip),
               data.frame(inf_eas, method = "SuSiE-inf-EAS", thr_pip = thr_pip),
               data.frame(susiex, method = "SuSiEx", thr_pip = thr_pip))
}
out$sig <- factor(ifelse(out$pval < 5e-8, "< 5e-8", "> 5e-8"),
                  levels = c("> 5e-8", "< 5e-8"))
out$piprange <- ifelse(out$pip_rep > 0.1, ">0.1", "<0.1")
out$piprange[out$pip_rep > 0.1 & out$pip_rep < 0.5] <- "0.1-0.5"
out$piprange[out$pip_rep > 0.5 & out$pip_rep < 0.9] <- "0.5-0.9"
out$piprange[out$pip_rep > 0.9] <- ">0.9"
out$piprange <- factor(out$piprange, levels = c("<0.1", "0.1-0.5", "0.5-0.9", ">0.9"))
out$thr_pip <- paste0("PIP threshold=",out$thr_pip)
out$method <- factor(out$method,levels=c("XMAP", "SuSiEx", "SuSiE-inf-EUR", "SuSiE-inf-EAS", "SuSiE-merge", "SuSiE-EUR", "SuSiE-EAS"))
```

### Figure 5C: Replication rate evaluated with GWAS p-value in BBJ¶

In [12]:

```
ggplot(out, aes(x = method, fill = sig)) +
  geom_bar(position = position_fill()) +
  geom_text(stat = 'count', aes(label = ..count..), position = position_fill(vjust = 0.5)) +
  scale_y_continuous(labels = scales::percent) +
  ylab("Number of SNPs") +
  facet_grid(.~thr_pip) +
  scale_fill_brewer(palette = "Paired") +
  theme_minimal() +
  coord_flip() +
  guides(fill=guide_legend(title="p-value in BBJ")) +
  theme(legend.position = "bottom")
```

### Figure 5D: Replication rate evaluated with SuSiE PIP in BBJ¶

In [13]:

```
ggplot(out, aes(x = method, fill = piprange)) +
  geom_bar(position = position_fill()) +
  geom_text(stat = 'count', aes(label = ..count..), position = position_fill(vjust = 0.5)) +
  scale_y_continuous(labels = scales::percent) +
  ylab("Percentage") +
  facet_grid(.~thr_pip) +
  scale_fill_brewer(palette = "Paired") +
  theme_minimal() +
  coord_flip() +
  guides(fill=guide_legend(title="PIP in BBJ")) +
  theme(legend.position = "bottom")
# 1400 * 350
```
