# Supplementary material for: XMAP: Cross-population fine-mapping by leveraging genetic diversity and accounting for confounding bias
Source: Nat Commun. 2023 Oct 28;14:6870. doi: 10.1038/s41467-023-42614-7 (PMC10613261; doi:10.1038/s41467-023-42614-7)
Supplement: Supplementary file 4 — Supplementary Software 1 [file 41467_2023_42614_MOESM4_ESM.zip › Software_submit_r3/reproduce/XMAP_adj_z.html]

XMAP\_adj\_z


In this demo, we show the equivalence between the individual-level XMAP (XMAP-i) and summary-level XMAP with adjusted z-scores.

In [1]:

```
library(susieR)
library(XMAP)
library(mvtnorm)
set.seed(1000)
n1 <- 10000
n2 <- 10000

p <- 500

# Generate genotype matrices
X1 <- matrix(rnorm(n1*p),n1,p)
X2 <- matrix(rnorm(n2*p),n2,p)

Xs1 <- scale(X1)
Xs2 <- scale(X2)


h1_omega <- 0.005
h2_omega <- 0.005
rho_omega <- 0.8
enrich <- 50

rho_sigma <- 0

# per-snp variance for bg
omega1 <- h1_omega / p
omega2 <- h2_omega / p
omega12 <- rho_omega * sqrt(omega1 * omega2)

Omega <- matrix(c(omega1, omega12, omega12, omega2), 2, 2)
Omega0 <- matrix(c(omega1, 0, 0, omega2), 2, 2)


# per-snp variance for causal
Sigma1 <- omega1 * enrich
Sigma2 <- omega2 * enrich
Sigma12 <- rho_sigma * sqrt(Sigma1 * Sigma2)
Sigma <- matrix(c(Sigma1, Sigma12, Sigma12, Sigma2), 2, 2)


# Generate polygenic effects
phi <- rmvnorm(p, rep(0, 2), Omega)

idx_causal <- c(5,10,15)
K_true <- 3

# Generate causal effects
beta <- matrix(0, p, 2)
beta[idx_causal,] <- rmvnorm(K_true, rep(0, 2), Sigma)

beta[, 1] <- beta[, 1]/sqrt(var(beta[,1]))*sqrt(Sigma[1,1]/p)
beta[, 2] <- beta[, 2]/sqrt(var(beta[,2]))*sqrt(Sigma[2,2]/p)

# Generate phenotypes
y1 <- Xs1 %*% beta[, 1] +
  Xs1 %*% phi[, 1] +
  rnorm(n1, 0, sqrt(1 - omega1 * p - Sigma1 * K_true))

y2 <- Xs2 %*% beta[, 2] +
  Xs2 %*% phi[, 2] +
  rnorm(n2, 0, sqrt(1 - omega2 * p - Sigma2 * K_true))

# Compute summary statistics
sumstats1 <- univariate_regression(Xs1, y1)
sumstats2 <- univariate_regression(Xs2, y2)

# Generate z-scores
zs1 <- sumstats1$betahat/sumstats1$sebetahat
zs2 <- sumstats2$betahat/sumstats2$sebetahat

# Compute LD matrices
R1 <- cor(X1)
R2 <- cor(X2)
```

In [2]:

```
# Run summary-level XMAP with adj_zs=T performs z-score adjustment before fitting
fit_xmap <- XMAP(R=simplify2array(list(R1,R2)), z=cbind(zs1,zs2),adj_zs = T,
                 n=c(n1,n2), K = 5,
                 Omega = Omega,
                 Sig_E =  c(1,1),
                 tol = 1e-6,
                 maxIter = 200, estimate_residual_variance = T, estimate_prior_variance = T,
                 estimate_background_variance = F)
str(fit_xmap)
```

```
List of 9
 $ gamma        : num [1:5, 1:500] 6.14e-05 2.17e-03 2.46e-03 2.46e-03 2.46e-03 ...
 $ mu           : num [1:2, 1:5, 1:500] -0.0119 0.00565 -0.00545 0.01154 -0.00335 ...
 $ nu           : num [1:2, 1:500] 1.33e-04 5.99e-04 -1.98e-04 1.69e-04 7.21e-05 ...
 $ Sigma        : num [1:2, 1:2, 1:5] 1.79e-03 -7.00e-04 -7.00e-04 2.85e-04 7.58e-05 ...
 $ Omega        : num [1:2, 1:2] 1e-05 8e-06 8e-06 1e-05
 $ Sig_E        : num [1:2, 1] 0.994 0.993
 $ ELBO         : num [1:12, 1] -Inf -28899 -28879 -28878 -28877 ...
 $ K            : int 5
 $ prior_weights: num [1:500, 1] 0.002 0.002 0.002 0.002 0.002 0.002 0.002 0.002 0.002 0.002 ...
```

In [3]:

```
# Run individual-level XMAP 
fit_ind <- XMAP_i(Xs1,Xs2,scale(y1),scale(y2),K=5,Sigma1=0.1,Sigma2 = 0.1,rho=0,
                            Sig_E1 = 1,Sig_E2=1,
                            Omega = Omega,
                            prior_weights = rep(1 / p, p), tol = 1e-6,
                            maxIter = 200, estimate_residual_variance = T, estimate_prior_variance = T,
                            estimate_background_variance = F, initialize = F)
str(fit_ind)
```

```
2 -th iteration finished.	 ELBO= -28899.06 ,	 Diff= Inf 
3 -th iteration finished.	 ELBO= -28878.84 ,	 Diff= 20.22003 
4 -th iteration finished.	 ELBO= -28877.57 ,	 Diff= 1.264356 
5 -th iteration finished.	 ELBO= -28877.19 ,	 Diff= 0.3840007 
6 -th iteration finished.	 ELBO= -28877.01 ,	 Diff= 0.1802179 
7 -th iteration finished.	 ELBO= -28876.91 ,	 Diff= 0.1045612 
8 -th iteration finished.	 ELBO= -28876.84 ,	 Diff= 0.0691544 
9 -th iteration finished.	 ELBO= -28876.79 ,	 Diff= 0.05013862 
10 -th iteration finished.	 ELBO= -28876.75 ,	 Diff= 0.03908225 
11 -th iteration finished.	 ELBO= -28876.71 ,	 Diff= 0.03238681 
12 -th iteration finished.	 ELBO= -28876.69 ,	 Diff= 0.0282558 
List of 21
 $ gamma         : num [1:5, 1:500] 6.14e-05 2.17e-03 2.46e-03 2.46e-03 2.46e-03 ...
 $ tgamma1       : NULL
 $ tgamma2       : NULL
 $ mu1           : num [1:5, 1:500] -0.0119 -0.00545 -0.00335 -0.00336 -0.00337 ...
 $ mu2           : num [1:5, 1:500] 0.00565 0.01154 0.00763 0.00762 0.00763 ...
 $ tmu1          : NULL
 $ tmu2          : NULL
 $ nu            : num [1:1000, 1] 1.33e-04 -1.98e-04 7.21e-05 -9.42e-04 1.06e-03 ...
 $ Sigma         : num [1:2, 1:2, 1:5] 1.79e-03 -7.00e-04 -7.00e-04 2.85e-04 7.58e-05 ...
 $ Eta1          : NULL
 $ Eta2          : NULL
 $ Omega         : num [1:2, 1:2] 1e-05 8e-06 8e-06 1e-05
 $ Sig_E1        : num 0.994
 $ Sig_E2        : num 0.993
 $ ELBO          : num [1:12] -Inf -28899 -28879 -28878 -28877 ...
 $ K             : num 5
 $ K1            : num 0
 $ K2            : num 0
 $ prior_weights : num [1:500] 0.002 0.002 0.002 0.002 0.002 0.002 0.002 0.002 0.002 0.002 ...
 $ prior_weights1: NULL
 $ prior_weights2: NULL
```

In [4]:

```
# Check equivalence of PIP
all.equal(get_pip(fit_xmap$gamma),get_pip(fit_ind$gamma))
```

TRUE

In [5]:

```
# Plot PIP
plot(get_pip(fit_xmap$gamma), get_pip(fit_ind$gamma),xlab = "XMAP",ylab = "XMAP-i")
abline(a=c(0,0),b=c(1,1),col="red")
points(get_pip(fit_xmap$gamma)[idx_causal],get_pip(fit_ind$gamma)[idx_causal],col="blue",pch=16)
```
