# Supplementary material for: XMAP: Cross-population fine-mapping by leveraging genetic diversity and accounting for confounding bias
Source: Nat Commun. 2023 Oct 28;14:6870. doi: 10.1038/s41467-023-42614-7 (PMC10613261; doi:10.1038/s41467-023-42614-7)
Supplement: Supplementary file 4 — Supplementary Software 1 [file 41467_2023_42614_MOESM4_ESM.zip › Software_submit_r3/reproduce/Fig4_LDL_ncausal.html]

Fig4\_LDL\_ncausal


# Figure 4 LDL¶

This notebook reproduces the Figure4 A and B in XMAP manuscript.

GWAS summary statistics can be found at https://hkustconnect-my.sharepoint.com/:f:/g/personal/mcaiad\_connect\_ust\_hk/EumV7yII87BAsaA-SuYw3ZcBo0Xa1OYbDy6T9hHeq51UJA?e=ulWzqq

PIP files required for running this script can be found at https://hkustconnect-my.sharepoint.com/:f:/g/personal/mcaiad\_connect\_ust\_hk/ElvWk3EXEEVCpej0aJM2dx0B0mECe8b\_L18KPdinu8UXEA?e=09G3ZE.

LD scores can be found at https://hkustconnect-my.sharepoint.com/:f:/g/personal/mcaiad\_connect\_ust\_hk/EnztsG0PeWBBkbQlJRswb-4BZz9Uihyr1PWikKp2WJFFBg?e=kYR8PV

In [1]:

```
library(susieR)
library(data.table)
library(Matrix)
library(XMAP)
library(ggplot2)

get_enrichment <- function(a, b, c, d) {
  enrichment <- (a / b) / (c / d)
  se_log <- sqrt(1 / a + 1 / c - 1 / b - 1 / d)
  CI95 <- c(exp(log(enrichment) - 2 * se_log), exp(log(enrichment) + 2 * se_log))
  dat <- data.frame(matrix(c(a, b, c, d), 2, 2))
  pval <- fisher.test(dat)$p.value

  return(list(OR = enrichment, se = se_log, CI95 = CI95, pval = pval))
}
```

In [2]:

```
cvg <- 0.99
min_cor <- 0.1

thr_pip_all <- c(0.8, 0.9, 0.95, 0.99)

sumstat_EUR <- fread("/home/share/sumstats/format/LDL_allSNPs_UKBNealLab_summary_format.txt", data.table = F)
sumstat_AFR <- fread("/home/share/sumstats/format/LDL_AFR_GLGC_summary_format.txt", data.table = F)
sumstat_EAS <- fread("/home/share/sumstats/format/LDL_EAS_GLGC_summary_format.txt", data.table = F)
snps_all <- Reduce(intersect, list(sumstat_EUR$SNP, sumstat_AFR$SNP, sumstat_EAS$SNP))
```

In [3]:

```
out_eur <- fread(file = "/home/share/mingxuan/fine_mapping/analysis/results/LDL_EUR_PIP.txt", data.table = F)
out_afr <- fread(file = "/home/share/mingxuan/fine_mapping/analysis/results/LDL_AFR_PIP.txt", data.table = F)
out_eas <- fread(file = "/home/share/mingxuan/fine_mapping/analysis/results/LDL_EAS_PIP.txt", data.table = F)

out_3pop <- fread(file = "/home/share/mingxuan/fine_mapping/analysis/results/LDL_3pop_PIP.txt", data.table = F)

out_eur_afr <- fread(file = "/home/share/mingxuan/fine_mapping/analysis/results/LDL_EUR_AFR_PIP.txt", data.table = F)

out_eur_eas <- fread(file = "/home/share/mingxuan/fine_mapping/analysis/results/LDL_EUR_EAS_PIP.txt", data.table = F)

out_afr_eas <- fread(file = "/home/share/mingxuan/fine_mapping/analysis/results/LDL_AFR_EAS_PIP.txt", data.table = F)

snps_liver <- fread("/home/share/mingxuan/eqtls/Liver.v8.EUR.unique_SNPs.rsid.txt", header = F)
snps_in_eqtl <- sum(snps_all %in% snps_liver$V1)

ldscore <- data.frame()
for (chr in 1:22) {
  ldscore_chr <- fread(paste0("/home/share/mingxuan/UKB_geno_finemap/LDscore_eas_brit_afr_chr", chr, ".txt"))
  ldscore <- rbind(ldscore, ldscore_chr)
  cat("CHR", chr, "\n")
}

out_enrichment <- data.frame()
out_signal <- data.frame()
out_ldscore <- data.frame()
for (thr_pip in thr_pip_all) {
  out_ldscore <- rbind(out_ldscore, data.frame(ldscore = melt(ldscore[match(out_3pop$rsid[out_3pop$PIP > thr_pip], ldscore$rsid), c("AFR", "EUR", "EAS")]),
                                               thr_pip = thr_pip, method = "XMAP"))

  out_signal <- rbind(out_signal, data.frame(nSNPs = sum(out_3pop$PIP > thr_pip), Population = "EUR+AFR+EAS", thr_pip = thr_pip, method = "XMAP"))
  out_signal <- rbind(out_signal, data.frame(nSNPs = sum(out_eur_afr$PIP > thr_pip), Population = "EUR+AFR", thr_pip = thr_pip, method = "XMAP"))
  out_signal <- rbind(out_signal, data.frame(nSNPs = sum(out_eur_eas$PIP > thr_pip), Population = "EUR+EAS", thr_pip = thr_pip, method = "XMAP"))
  out_signal <- rbind(out_signal, data.frame(nSNPs = sum(out_afr_eas$PIP > thr_pip), Population = "AFR+EAS", thr_pip = thr_pip, method = "XMAP"))
  out_signal <- rbind(out_signal, data.frame(nSNPs = sum(out_eur$PIP > thr_pip), Population = "EUR", thr_pip = thr_pip, method = "SuSiE"))
  out_signal <- rbind(out_signal, data.frame(nSNPs = sum(out_afr$PIP > thr_pip), Population = "AFR", thr_pip = thr_pip, method = "SuSiE"))
  out_signal <- rbind(out_signal, data.frame(nSNPs = sum(out_eas$PIP > thr_pip), Population = "EAS", thr_pip = thr_pip, method = "SuSiE"))


  pop3 <- get_enrichment(sum(out_3pop$rsid[out_3pop$PIP > thr_pip] %in% snps_liver$V1), sum(out_3pop$PIP > thr_pip), snps_in_eqtl, length(snps_all))

  eur_afr <- get_enrichment(sum(out_eur_afr$rsid[out_eur_afr$PIP > thr_pip] %in% snps_liver$V1), sum(out_eur_afr$PIP > thr_pip), snps_in_eqtl, length(snps_all))
  eur_eas <- get_enrichment(sum(out_eur_eas$rsid[out_eur_eas$PIP > thr_pip] %in% snps_liver$V1), sum(out_eur_eas$PIP > thr_pip), snps_in_eqtl, length(snps_all))
  afr_eas <- get_enrichment(sum(out_afr_eas$rsid[out_afr_eas$PIP > thr_pip] %in% snps_liver$V1), sum(out_afr_eas$PIP > thr_pip), snps_in_eqtl, length(snps_all))

  eur <- get_enrichment(sum(out_eur$rsid[out_eur$PIP > thr_pip] %in% snps_liver$V1), sum(out_eur$PIP > thr_pip), snps_in_eqtl, length(snps_all))
  afr <- get_enrichment(sum(out_afr$rsid[out_afr$PIP > thr_pip] %in% snps_liver$V1), sum(out_afr$PIP > thr_pip), snps_in_eqtl, length(snps_all))
  eas <- get_enrichment(sum(out_eas$rsid[out_eas$PIP > thr_pip] %in% snps_liver$V1), sum(out_eas$PIP > thr_pip), snps_in_eqtl, length(snps_all))

  out_enrichment <- rbind(out_enrichment, data.frame(Enrichment = pop3$OR, se = pop3$se, pval = pop3$pval,
                                                     Population = "EUR+AFR+EAS", thr_pip = thr_pip, method = "XMAP"))
  out_enrichment <- rbind(out_enrichment, data.frame(Enrichment = eur_afr$OR, se = eur_afr$se, pval = eur_afr$pval,
                                                     Population = "EUR+AFR", thr_pip = thr_pip, method = "XMAP"))
  out_enrichment <- rbind(out_enrichment, data.frame(Enrichment = eur_eas$OR, se = eur_eas$se, pval = eur_eas$pval,
                                                     Population = "EUR+EAS", thr_pip = thr_pip, method = "XMAP"))
  out_enrichment <- rbind(out_enrichment, data.frame(Enrichment = afr_eas$OR, se = afr_eas$se, pval = afr_eas$pval,
                                                     Population = "AFR+EAS", thr_pip = thr_pip, method = "XMAP"))
  out_enrichment <- rbind(out_enrichment, data.frame(Enrichment = eur$OR, se = eur$se, pval = eur$pval,
                                                     Population = "EUR", thr_pip = thr_pip, method = "SuSiE"))
  out_enrichment <- rbind(out_enrichment, data.frame(Enrichment = afr$OR, se = afr$se, pval = afr$pval,
                                                     Population = "AFR", thr_pip = thr_pip, method = "SuSiE"))
  out_enrichment <- rbind(out_enrichment, data.frame(Enrichment = eas$OR, se = eas$se, pval = eas$pval,
                                                     Population = "EAS", thr_pip = thr_pip, method = "SuSiE"))
  cat(thr_pip, " finished.\n")
}
out_signal$thr_pip <- paste0("PIP>", out_signal$thr_pip)
out_ldscore$thr_pip <- paste0("PIP>", out_ldscore$thr_pip)
```

```
CHR 1 
CHR 2 
CHR 3 
CHR 4 
CHR 5 
CHR 6 
CHR 7 
CHR 8 
CHR 9 
CHR 10 
CHR 11 
CHR 12 
CHR 13 
CHR 14 
CHR 15 
CHR 16 
CHR 17 
CHR 18 
CHR 19 
CHR 20 
CHR 21 
CHR 22
```

```
Warning message in melt.data.table(ldscore[match(out_3pop$rsid[out_3pop$PIP > thr_pip], :
“id.vars and measure.vars are internally guessed when both are 'NULL'. All non-numeric/integer/logical type columns are considered id.vars, which in this case are columns []. Consider providing at least one of 'id' or 'measure' vars in future.”
```

```
0.8  finished.
```

```
Warning message in melt.data.table(ldscore[match(out_3pop$rsid[out_3pop$PIP > thr_pip], :
“id.vars and measure.vars are internally guessed when both are 'NULL'. All non-numeric/integer/logical type columns are considered id.vars, which in this case are columns []. Consider providing at least one of 'id' or 'measure' vars in future.”
```

```
0.9  finished.
```

```
Warning message in melt.data.table(ldscore[match(out_3pop$rsid[out_3pop$PIP > thr_pip], :
“id.vars and measure.vars are internally guessed when both are 'NULL'. All non-numeric/integer/logical type columns are considered id.vars, which in this case are columns []. Consider providing at least one of 'id' or 'measure' vars in future.”
```

```
0.95  finished.
```

```
Warning message in melt.data.table(ldscore[match(out_3pop$rsid[out_3pop$PIP > thr_pip], :
“id.vars and measure.vars are internally guessed when both are 'NULL'. All non-numeric/integer/logical type columns are considered id.vars, which in this case are columns []. Consider providing at least one of 'id' or 'measure' vars in future.”
```

```
0.99  finished.
```

In [4]:

```
out_signal$Population <- factor(out_signal$Population,levels = c("EUR+AFR+EAS","EUR+AFR","EUR+EAS","AFR+EAS","EUR","AFR","EAS"))
out_signal$method <- factor(out_signal$method,levels = c("XMAP","SuSiE"))
ggplot(out_signal, aes(x = method, y = nSNPs, fill = Population)) +
  geom_bar(stat = "identity", color = "black",
           position = position_dodge()) +
  ylab("Number of Causal SNPs") +
  facet_grid(. ~ thr_pip) +
  theme_bw() +
  guides(fill = guide_legend(nrow = 1))+
  theme(legend.position = "bottom")
```

In [5]:

```
colnames(out_ldscore)[1:2] <- c("Population","LD Score")
ggplot(out_ldscore, aes(x = thr_pip, y = `LD Score`, fill = Population)) +
  labs(x ="PIP Threshold", y="LD Score")+
    geom_boxplot()+
  theme_bw() +
  theme(legend.position = "bottom")
```

In [6]:

```
out_enrichment$thr_pip <- paste0("PIP>", out_enrichment$thr_pip)
out_enrichment$method <- factor(out_enrichment$method,levels = c("XMAP","SuSiE"))
out_enrichment$Population <- factor(out_enrichment$Population,levels = c("EUR+AFR+EAS","EUR+AFR","EUR+EAS","AFR+EAS","EUR","AFR","EAS"))

ggplot(out_enrichment[out_enrichment$method != "XMAP (C=I)",], aes(x = method, y = -log10(pval), fill = Population)) +
  geom_bar(stat = "identity", color = "black",
           position = position_dodge()) +
  ylab("Enrichment in liver eQTL")+
  theme_bw() +
  facet_grid(. ~ thr_pip) +
  guides(fill = guide_legend(nrow = 1))+
  theme(legend.position = "bottom")
```
