# Supplementary material for: XMAP: Cross-population fine-mapping by leveraging genetic diversity and accounting for confounding bias
Source: Nat Commun. 2023 Oct 28;14:6870. doi: 10.1038/s41467-023-42614-7 (PMC10613261; doi:10.1038/s41467-023-42614-7)
Supplement: Supplementary file 4 — Supplementary Software 1 [file 41467_2023_42614_MOESM4_ESM.zip › Software_submit_r3/reproduce/Fig6_height_multiple_signal_r1.html]

Fig6\_height\_multiple\_signal\_r1


# Figure 6 height¶

This notebook reproduces the Figure 6 A-D in XMAP manuscript.
PIP files required for running this script can be found at https://hkustconnect-my.sharepoint.com/:f:/g/personal/mcaiad\_connect\_ust\_hk/Eptgw2McNLRKjsypetdRwzABaA\_JM20jEeGgSwwc\_GfK1g?e=XNloZD
loci information can be found at https://hkustconnect-my.sharepoint.com/:f:/g/personal/mcaiad\_connect\_ust\_hk/EhMiNgq0EulAkpTNk9d4b2ABKgfUBgIteHgCIlDbMZHC6Q?e=3h0DNh

In [1]:

```
library(ggplot2)
library(data.table)
library(dplyr)
library(ggrepel)

thr_all <- c(0.5,0.8, 0.9,0.95,0.99)

K <- 10
```

```
Attaching package: ‘dplyr’


The following objects are masked from ‘package:data.table’:

    between, first, last


The following objects are masked from ‘package:stats’:

    filter, lag


The following objects are masked from ‘package:base’:

    intersect, setdiff, setequal, union
```

In [2]:

```
pip_xmap <- fread(paste0("/home/share/mingxuan/fine_mapping/analysis/results/height_UKB_WG_K", K, "_PIP.txt"))
pip_susiex <- fread(paste0("/home/share/mingxuan/fine_mapping/analysis/results/height_susiex_success_UKB_WG_K", K, "_PIP.txt"))
pip_susiex$PIP[is.na(pip_susiex$PIP)] <- 0
pip_eas <- fread(paste0("/home/share/mingxuan/fine_mapping/analysis/results/height_WG_K", K, "_PIP.txt"))
pip_eur <- fread(paste0("/home/share/mingxuan/fine_mapping/analysis/results/height_UKB_K", K, "_PIP.txt"))

out <- out_multi <- data.frame()
for (chr in 1:22) {

  loci <- fread(paste0("/home/share/mingxuan/UKB_geno_finemap/chr", chr, "_eas_brit_afr.loci"))
  pip_xmap_chr <- pip_xmap[pip_xmap$chromosome == chr,]
  pip_susiex_chr <- pip_susiex[pip_susiex$chromosome == chr,]
  pip_eas_chr <- pip_eas[pip_eas$chromosome == chr,]
  pip_eur_chr <- pip_eur[pip_eur$chromosome == chr,]

  for (i in 1:nrow(loci)) {
    pip_xmap_i <- pip_xmap_chr[pip_xmap_chr$position > loci$start[i] & pip_xmap_chr$position < loci$end[i],]
    pip_susiex_i <- pip_susiex_chr[pip_susiex_chr$position > loci$start[i] & pip_susiex_chr$position < loci$end[i],]
    pip_eas_i <- pip_eas_chr[pip_eas_chr$position > loci$start[i] & pip_eas_chr$position < loci$end[i],]
    pip_eur_i <- pip_eur_chr[pip_eur_chr$position > loci$start[i] & pip_eur_chr$position < loci$end[i],]
    for (thr in thr_all) {
      if (sum(pip_xmap_i$PIP > thr) > 0) {
        out_multi <- rbind(out_multi, data.frame(pip_xmap_i[pip_xmap_i$PIP > thr,], method = "XMAP", nsnp = sum(pip_xmap_i$PIP > thr), thr = thr))
      }
      if (sum(pip_susiex_i$PIP > thr) > 0) {
        out_multi <- rbind(out_multi, data.frame(pip_susiex_i[pip_susiex_i$PIP > thr,], method = "SuSiEx", nsnp = sum(pip_susiex_i$PIP > thr), thr = thr))
      }
      if (sum(pip_eas_i$PIP > thr) > 0) {
        out_multi <- rbind(out_multi, data.frame(pip_eas_i[pip_eas_i$PIP > thr,], method = "SuSiE-EAS", nsnp = sum(pip_eas_i$PIP > thr), thr = thr))
      }
      if (sum(pip_eur_i$PIP > thr) > 0) {
        out_multi <- rbind(out_multi, data.frame(pip_eur_i[pip_eur_i$PIP > thr,], method = "SuSiE-EUR", nsnp = sum(pip_eur_i$PIP > thr), thr = thr))
      }
      out <- rbind(out, data.frame(sum(pip_xmap_i$PIP > thr), sum(pip_eas_i$PIP > thr), sum(pip_eur_i$PIP > thr), thr = thr))
    }
  }
  cat(chr, "-th chr finished.\n")
}
out_multi$thr <- paste0("PIP threshold=", out_multi$thr)
out_multi$method <- factor(out_multi$method, levels = c("XMAP", "SuSiEx", "SuSiE-EUR", "SuSiE-EAS"))
```

```
1 -th chr finished.
2 -th chr finished.
3 -th chr finished.
4 -th chr finished.
5 -th chr finished.
6 -th chr finished.
7 -th chr finished.
8 -th chr finished.
9 -th chr finished.
10 -th chr finished.
11 -th chr finished.
12 -th chr finished.
13 -th chr finished.
14 -th chr finished.
15 -th chr finished.
16 -th chr finished.
17 -th chr finished.
18 -th chr finished.
19 -th chr finished.
20 -th chr finished.
21 -th chr finished.
22 -th chr finished.
```

## Figure 6 B-C¶

In [3]:

```
############# Main Fig replicate in Sibship cumulative nsnp #############
out_cum <- out_multi[out_multi$nsnp > 1,]
out_cum$nsnp_cum <- "Loci with >1 SNPs"
out_cum <- rbind(out_cum, data.frame(out_multi[out_multi$nsnp > 2,], nsnp_cum = "Loci with >2 SNPs"))

pip_sibship <- readRDS("/home/share/mingxuan/fine_mapping/analysis/results/height_sibship_pip_allSNPs.RDS")
sumstat_EUR <- fread("/home/share/sumstats/format/height_EUR_sibship_summary_format.txt")
snps <- intersect(pip_sibship$rsid, sumstat_EUR$SNP)
pip_sibship <- pip_sibship[match(snps, pip_sibship$rsid),]
sumstat_EUR <- sumstat_EUR[match(snps, sumstat_EUR$SNP),]
pval <- 2 * pnorm(abs(sumstat_EUR$Z), lower.tail = F)

idx_rep <- match(out_cum$rsid, snps)
out_cum$pip_rep <- pip_sibship$PIP[idx_rep]
out_cum$pval_rep <- pval[idx_rep]

out_cum$sig <- factor(ifelse(is.na(out_cum$pval_rep), "Not present", ifelse(out_cum$pval_rep < 5e-8, "< 5e-8", ifelse(out_cum$pval_rep < 1e-6, "1e-6 - 5e-8", "> 1e-6"))),
                      levels = c("Not present", "> 1e-6", "1e-6 - 5e-8", "< 5e-8"))
# out$replicated <- ifelse(out$pip > 0.1, "PIP>0.1 in GIANT", "PIP<0.1 in GIANT")
out_cum$piprange <- ifelse(is.na(out_cum$pip_rep), "Not present", ifelse(out_cum$pip_rep > 0.1, ">0.1", "<0.1"))
out_cum$piprange[!is.na(out_cum$pip_rep) &
                   out_cum$pip_rep > 0.1 &
                   out_cum$pip_rep < 0.5] <- "0.1-0.5"
out_cum$piprange[!is.na(out_cum$pip_rep) &
                   out_cum$pip_rep > 0.5 &
                   out_cum$pip_rep < 0.9] <- "0.5-0.9"
out_cum$piprange[!is.na(out_cum$pip_rep) & out_cum$pip_rep > 0.9] <- ">0.9"
out_cum$piprange <- factor(out_cum$piprange, levels = c("Not present", "<0.1", "0.1-0.5", "0.5-0.9", ">0.9"))
```

In [4]:

```
options(repr.plot.width=18, repr.plot.height=3)
ggplot(out_cum[out_cum$thr %in% paste0("PIP threshold=", c(0.9)) &
                 out_cum$sig != "Not present",], aes(x = method, fill = sig)) +
  geom_bar(position = position_fill()) +
  geom_text(stat = 'count', aes(label = ..count..), position = position_fill(vjust = 0.5)) +
  scale_y_continuous(labels = scales::percent) +
  ylab("Number of SNPs") +
  # xlab("") +
  facet_grid(. ~ nsnp_cum) +
  scale_fill_brewer(palette = "Paired") +
  theme_minimal() +
  coord_flip() +
  guides(fill = guide_legend(title = "p-value in sibship GWAS")) +
  theme(legend.position = "bottom", axis.title.y = element_blank())

ggplot(out_cum[out_cum$thr =="PIP threshold=0.9" &
                 out_cum$sig != "Not present",], aes(x = method, fill = piprange)) +
  geom_bar(position = position_fill()) +
  geom_text(stat = 'count', aes(label = ..count..), position = position_fill(vjust = 0.5)) +
  scale_y_continuous(labels = scales::percent) +
  ylab("Percentage") +
  # xlab("") +
  facet_grid(. ~ nsnp_cum) +
  scale_fill_brewer(palette = "Paired") +
  theme_minimal() +
  coord_flip() +
  guides(fill = guide_legend(title = "PIP in sibship GWAS")) +
  theme(legend.position = "bottom", axis.title.y = element_blank())
```

```
Warning message:
“The dot-dot notation (`..count..`) was deprecated in ggplot2 3.4.0.
ℹ Please use `after_stat(count)` instead.”
```

## Figure6 A¶

In [5]:

```
################### Main Fig summarize XMAP # snps in each locus ###################
out_xmap <- out[, c(1, 4)]
colnames(out_xmap) <- c("nloci", "thr")
out_xmap$thr <- paste0("PIP>", out_xmap$thr)
count_xmap <- dplyr::count(out_xmap[out_xmap$nloci != 0,], nloci, thr)


count_xmap$nloci <- factor(count_xmap$nloci, levels = as.character(max(count_xmap$nloci):1))
count_xmap <- count_xmap %>%
  group_by(thr) %>%
  mutate(label_y = cumsum(n) - 0.5 * n)

ggplot(count_xmap, aes(x = thr, y = n, fill = nloci, order = nloci)) +
  geom_bar(stat = "identity") +
  xlab("PIP threshold") +
  ylab("Number of loci") +
  guides(fill = guide_legend(title = "Number of identified SNPs")) +
  theme_classic() +
  coord_flip() +
  theme(legend.position = c(0.75, 0.8), legend.direction = "horizontal") +
  scale_y_continuous(expand = expansion(mult = c(0.01, 0.01))) +
  scale_fill_brewer(palette = "Pastel1", direction = -1) +
  geom_text(aes(label = n, y = label_y), size = 3)
# 1200 * 200
############################################################################
```
