# Supplementary material for: XMAP: Cross-population fine-mapping by leveraging genetic diversity and accounting for confounding bias
Source: Nat Commun. 2023 Oct 28;14:6870. doi: 10.1038/s41467-023-42614-7 (PMC10613261; doi:10.1038/s41467-023-42614-7)
Supplement: Supplementary file 4 — Supplementary Software 1 [file 41467_2023_42614_MOESM4_ESM.zip › Software_submit_r3/reproduce/Fig7_SCAVENGE.html]

Figure7\_SCAVENGE


# Figure 7¶

This notebook reproduces the Figure 7 in XMAP manuscript.

PIP files of 12 blood traits can be found at https://hkustconnect-my.sharepoint.com/:f:/g/personal/mcaiad\_connect\_ust\_hk/EiexnAhCaEZJj-1A3EhOpwQBAJZPF6eMYuiMQf0QH8flzg?e=NErpj7

SCAVENGE output can be found at https://hkustconnect-my.sharepoint.com/:f:/g/personal/mcaiad\_connect\_ust\_hk/EkcuIxsmluFDrFcWj7C3Ly8BfrWwJ-LeuQZxC-MODjbXUw?e=Rh7ZPv

In [5]:

```
library(SCAVENGE)
library(chromVAR)
library(gchromVAR)
library(SummarizedExperiment)
library(data.table)
library(BiocParallel)
library(BSgenome.Hsapiens.UCSC.hg19)
library(dplyr)
library(igraph)
library(ggplot2)
library(RColorBrewer)
library(ggridges)
library(dplyr)
library(grid)
library(reshape2)
library(RColorBrewer)
library(ggrepel)
```

In [2]:

```
all_traits <- c("Lym", "WBC", "Neutro", "Mono", "Eosino", "Baso", "Plt", "RBC", "MCH", "MCHC", "MCV", "Hb")
method <- "xmap"
# method <- "ukb"
# method <- "bbj"
out_all <- data.frame()
for (i in 1:length(all_traits)) {
  trait <- all_traits[i]
  trait_mat <- fread(paste0("/home/share/mingxuan/fine_mapping/analysis/results/scavenge_output_", trait, "_", method, ".txt"))
  out_all <- rbind(out_all, data.frame(trait_mat, trait = all_traits[i]))
  cat(i, "-th trait finished.\n")
}
out_all$BioClassification <- factor(out_all$BioClassification,
                                    levels = c("Late.Eryth", "Early.Eryth", "Early.Baso", "CD14.Mono.1", "GMP.Neut", "cDC", "GMP", "CD14.Mono.2", "HSC", "CMP.LMPP", "CLP.1", "CLP.2", "Plasma", "B", "Pre.B", "pDC", "CD8.EM", "CD8.CM", "CD4.M", "CD8.N", "NK", "CD4.N1", "CD4.N2"))
```

```
1 -th trait finished.
2 -th trait finished.
3 -th trait finished.
4 -th trait finished.
5 -th trait finished.
6 -th trait finished.
7 -th trait finished.
8 -th trait finished.
9 -th trait finished.
10 -th trait finished.
11 -th trait finished.
12 -th trait finished.
```

## Figure 7A¶

In [6]:

```
################################## UMAP ##################################
class_avg <- trait_mat %>%
  group_by(BioClassification) %>%
  summarise(
    UMAP1 = median(UMAP1),
    UMAP2 = median(UMAP2)
  )

colourCount = length(unique(trait_mat$BioClassification))
mycolors <- colorRampPalette(brewer.pal(12, "Paired"))(colourCount)
p <- ggplot(data = trait_mat, aes(UMAP1, UMAP2, color = BioClassification)) +
  geom_point(size = 1, na.rm = TRUE) +
  geom_text(aes(label = BioClassification), data = class_avg, color = "black") +
  scale_color_manual(values = mycolors) +
  xlab("UMAP 1") +
  ylab("UMAP 2") +
  theme_classic() +
  theme(legend.title = element_blank(), legend.position = "none")
p
```

In [7]:

```
viridis = c("#440154FF", "#472D7BFF", "#3B528BFF", "#2C728EFF", "#21908CFF", "#27AD81FF", "#5DC863FF", "#AADC32FF", "#FDE725FF")

# bar plot of prop
out_all$label <- out_all$label_text <- as.character(out_all$BioClassification)
out_prop <- out_all %>%
  group_by(true_cell_top_idx, trait) %>%
  count(BioClassification) %>%
  mutate(cell_prop = n / sum(n)) %>%
  ungroup
for (i in 1:nrow(out_prop)) {
  if (out_prop$cell_prop[i] < 0.03) {
    out_all$label_text[out_all$trait == out_prop$trait[i] &
                         out_all$BioClassification == out_prop$BioClassification[i] &
                         out_all$true_cell_top_idx == out_prop$true_cell_top_idx[i]] <- "Other"
    out_all$label[out_all$trait == out_prop$trait[i] &
                    out_all$BioClassification == out_prop$BioClassification[i] &
                    out_all$true_cell_top_idx == out_prop$true_cell_top_idx[i]] <- "Other"
  } else if (out_prop$cell_prop[i] < 0.05) {
    idx <- which(out_all$trait == out_prop$trait[i] &
                   out_all$BioClassification == out_prop$BioClassification[i] &
                   out_all$true_cell_top_idx == out_prop$true_cell_top_idx[i] &
                   nchar(out_all$label_text) > 5)
    out_all$label_text[idx] <- sub("[.]", ".\n", out_all$label_text[idx])
  }
}
# Define the number of colors you want
mycolors <- colorRampPalette(brewer.pal(12, "Paired"))(length(unique(out_all$label)))
ggplot(out_all[out_all$true_cell_top_idx,], aes(x = trait, fill = label)) +
  geom_bar(position = position_fill()) +
  geom_text(stat = 'count', aes(label = label_text), position = position_fill(vjust = 0.5), size = 2.6, lineheight = 0.8) +
  # geom_text_repel(stat = 'count', aes(label = label), position = position_fill(vjust = 0.5),direction = "x") +
  scale_fill_manual(values = mycolors) +
  theme_classic() +
  theme(legend.position = "none") +
  scale_y_continuous(labels = scales::percent_format(scale = 100), limits = c(0, 1), expand = expansion(mult = c(0.01, 0.01))) +
  ylab("Trait-enriched cell proportion") +
  xlab(element_blank()) +
  coord_flip() +
  labs(fill = "Cell type")
#1500*400 120
```

## Figure 7B¶

In [8]:

```
# bar plot of count
out_all$label_text <- as.character(out_all$BioClassification)
out_prop <- out_all %>%
  group_by(true_cell_top_idx, trait) %>%
  count(BioClassification) %>%
  # mutate(cell_prop = n / sum(n)) %>%
  ungroup
# for (i in 1:nrow(out_prop)) {
#   if (out_prop$cell_prop[i] < 0.1) {
#     out_all$label_text[out_all$trait == out_prop$trait[i] &
#                          out_all$BioClassification == out_prop$BioClassification[i] &
#                          out_all$true_cell_top_idx == out_prop$true_cell_top_idx[i]] <- NA
#   }
# }
for (i in 1:nrow(out_prop)) {
  if ( out_prop$n[i]/nchar(as.character(out_prop$BioClassification[i])) < 50) {
    out_all$label_text[out_all$trait == out_prop$trait[i] &
                         out_all$BioClassification == out_prop$BioClassification[i] &
                         out_all$true_cell_top_idx == out_prop$true_cell_top_idx[i]] <- NA
  }
}
# Define the number of colors you want
mycolors <- colorRampPalette(brewer.pal(12, "Paired"))(length(unique(out_all$BioClassification)))
ggplot(out_all[out_all$true_cell_top_idx,], aes(x = trait, fill = as.character(BioClassification))) +
  geom_bar(position = position_stack(vjust = 0.5)) +
  geom_text(stat = 'count', aes(label = label_text), position = position_stack(vjust = 0.5), lineheight = 0.8) +
  # geom_text_repel(stat = 'count', aes(label = label), position = position_fill(vjust = 0.5),direction = "x") +
  scale_fill_manual(values = mycolors) +
  theme_classic() +
  theme(legend.position = "none") +
  scale_y_continuous(expand = expansion(mult = c(0.01, 0.01))) +
  ylab("Trait-enriched cell count") +
  xlab(element_blank()) +
  coord_flip() +
  labs(fill = "Cell type")
#1500*400 120
```

```
Warning message:
“Removed 217 rows containing missing values (geom_text).”
```

## Figure 7 C-J¶

In [10]:

```
for (i in 1:length(all_traits)) {
  trait_name <- all_traits[i]
  p_umap <- ggplot(data = out_all[out_all$trait == trait_name,], aes(UMAP1, UMAP2, color = TRS)) +
    geom_point(size = 1, na.rm = TRUE, alpha = 0.6) +
    scale_color_gradientn(colors = viridis) +
    scale_alpha() +
    theme_classic() +
    theme(legend.title = element_text(size = 18),
          legend.text = element_text(size = 14),
          legend.position = c(0.8, 0.8),
          axis.title = element_text(size = 18)) +
    xlab("UMAP 1") +
    ylab("UMAP 2") +
    annotate("text", x = -9, y = 13, label = trait_name, size = 8)
  print(p_umap)
  #size: 750*650

  ############# Enriched cell prop boxplot-circle #############
  p_bar <- out_all[out_all$trait == trait_name,] %>%
    group_by(BioClassification, trait) %>%
    summarise(enriched_cell = mean(true_cell_top_idx)) %>%
    ggplot(aes(x = BioClassification, y = enriched_cell, fill = BioClassification)) +
    geom_bar(stat = "identity", position = position_dodge()) +
    geom_text(aes(y = enriched_cell, label = scales::percent(enriched_cell, accuracy = 0.1)), position = position_dodge(width = 0.8)) +
    theme_bw() +
    scale_y_continuous(labels = scales::percent_format(scale = 100), limits = c(-1, 1.05), breaks = c(0, 0.25, 0.5, 0.75, 1)) +
    ylab("Trait-enriched cell proportion") +
    xlab(element_blank()) +
    theme(
      panel.border = element_blank(),
      plot.margin = unit(rep(0, 4), "cm"),
      legend.position = "none",
      axis.text.x = element_text(size = 13),
      axis.text.y = element_text(size = 13),
      axis.title.y = element_text(size = 18)
    ) +
    labs(fill = "Cell type") +
    coord_polar(start = 0) +
    annotate("text", x = 0, y = -1, label = trait_name, size = 8)
  #size: 1000*950
  print(p_bar)

}
```

```
`summarise()` has grouped output by 'BioClassification'. You can override using the `.groups` argument.
```

```
`summarise()` has grouped output by 'BioClassification'. You can override using the `.groups` argument.
```

```
`summarise()` has grouped output by 'BioClassification'. You can override using the `.groups` argument.
```

```
`summarise()` has grouped output by 'BioClassification'. You can override using the `.groups` argument.
```

```
`summarise()` has grouped output by 'BioClassification'. You can override using the `.groups` argument.
```

```
`summarise()` has grouped output by 'BioClassification'. You can override using the `.groups` argument.
```

```
`summarise()` has grouped output by 'BioClassification'. You can override using the `.groups` argument.
```

```
`summarise()` has grouped output by 'BioClassification'. You can override using the `.groups` argument.
```

```
`summarise()` has grouped output by 'BioClassification'. You can override using the `.groups` argument.
```

```
`summarise()` has grouped output by 'BioClassification'. You can override using the `.groups` argument.
```

```
`summarise()` has grouped output by 'BioClassification'. You can override using the `.groups` argument.
```

```
`summarise()` has grouped output by 'BioClassification'. You can override using the `.groups` argument.
```
