# Supplementary material for: XMAP: Cross-population fine-mapping by leveraging genetic diversity and accounting for confounding bias
Source: Nat Commun. 2023 Oct 28;14:6870. doi: 10.1038/s41467-023-42614-7 (PMC10613261; doi:10.1038/s41467-023-42614-7)
Supplement: Supplementary file 4 — Supplementary Software 1 [file 41467_2023_42614_MOESM4_ESM.zip › Software_submit_r3/reproduce/Fig_suppl30_LDL_replicate.html]

Fig\_suppl30\_LDL\_replicate


# Supplementary Figure 30: Replication analysis of LDL¶

This notebook reproduces the Supplementary Figure 30 XMAP Supplementary Note.

GWAS summary statistics can be found at https://hkustconnect-my.sharepoint.com/:f:/g/personal/mcaiad\_connect\_ust\_hk/EumV7yII87BAsaA-SuYw3ZcBo0Xa1OYbDy6T9hHeq51UJA?e=ulWzqq

PIP files required for running this script can be found at https://hkustconnect-my.sharepoint.com/:f:/g/personal/mcaiad\_connect\_ust\_hk/ElvWk3EXEEVCpej0aJM2dx0B0mECe8b\_L18KPdinu8UXEA?e=09G3ZE.

In [1]:

```
library(susieR)
library(data.table)
library(Matrix)
library(ggplot2)
library(RhpcBLASctl)
set.seed(1)
blas_set_num_threads(20)


thr_pip_all <- c(0.8, 0.9, 0.95, 0.99)
```

In [2]:

```
# Read summary data
sumstat_EUR <- fread("/home/share/sumstats/format/LDL_allSNPs_UKBNealLab_summary_format.txt", data.table = F)
sumstat_AFR <- fread("/home/share/sumstats/format/LDL_AFR_GLGC_summary_format.txt", data.table = F)
sumstat_EAS <- fread("/home/share/sumstats/format/LDL_EAS_GLGC_summary_format.txt", data.table = F)
sumstat_GLGC <- fread("/import/home/share/sumstats/format/LDL_EUR_GLGC_nonUKB_summary_format.txt", data.table = F)

# SuSiE results
out_eur <- fread(file = "/home/share/mingxuan/fine_mapping/analysis/results/LDL_EUR_PIP.txt", data.table = F)
out_afr <- fread(file = "/home/share/mingxuan/fine_mapping/analysis/results/LDL_AFR_PIP.txt", data.table = F)
out_eas <- fread(file = "/home/share/mingxuan/fine_mapping/analysis/results/LDL_EAS_PIP.txt", data.table = F)


# SuSiE-inf results
out_inf_eas <- fread(file = "/home/share/mingxuan/fine_mapping/analysis/results/LDL_sussieinf_EAS_PIP.txt", data.table = F)
out_inf_eur <- fread(file = "/home/share/mingxuan/fine_mapping/analysis/results/LDL_sussieinf_EUR_PIP.txt", data.table = F)
out_inf_afr <- fread(file = "/home/share/mingxuan/fine_mapping/analysis/results/LDL_sussieinf_AFR_PIP.txt", data.table = F)
# set PIP of  unconverged loci to 0
out_inf_eas$PIP[!out_inf_eas$converge] <- 0
out_inf_eur$PIP[!out_inf_eur$converge] <- 0
out_inf_afr$PIP[!out_inf_afr$converge] <- 0


# XMAP results
out_3pop <- fread(file = "/home/share/mingxuan/fine_mapping/analysis/results/LDL_3pop_PIP.txt", data.table = F)

out_eur_afr <- fread(file = "/home/share/mingxuan/fine_mapping/analysis/results/LDL_EUR_AFR_PIP.txt", data.table = F)

out_eur_eas <- fread(file = "/home/share/mingxuan/fine_mapping/analysis/results/LDL_EUR_EAS_PIP.txt", data.table = F)

out_afr_eas <- fread(file = "/home/share/mingxuan/fine_mapping/analysis/results/LDL_AFR_EAS_PIP.txt", data.table = F)

# SuSiEx results
out_ssx_3pop <- fread(file = "/import/home/share/mingxuan/fine_mapping/analysis/results/LDL_sussiex_success_3pop_PIP.txt", data.table = F)
out_ssx_3pop$PIP[is.na(out_ssx_3pop$PIP)] <- 0
out_ssx_eas_afr <- fread(file = "/import/home/share/mingxuan/fine_mapping/analysis/results/LDL_sussiex_success_EAS_AFR_PIP.txt", data.table = F)
out_ssx_eas_afr$PIP[is.na(out_ssx_eas_afr$PIP)] <- 0
out_ssx_eas_eur <- fread(file = "/import/home/share/mingxuan/fine_mapping/analysis/results/LDL_sussiex_success_EAS_EUR_PIP.txt", data.table = F)
out_ssx_eas_eur$PIP[is.na(out_ssx_eas_eur$PIP)] <- 0
out_ssx_afr_eur <- fread(file = "/import/home/share/mingxuan/fine_mapping/analysis/results/LDL_sussiex_success_AFR_EUR_PIP.txt", data.table = F)
out_ssx_afr_eur$PIP[is.na(out_ssx_afr_eur$PIP)] <- 0


# PIP of Replication data (GLGC-nonUKB) obtained with SuSiE
out_glgc <- fread("/home/share/mingxuan/fine_mapping/analysis/results/LDL_EUR_GLGC_nonUKB_PIP.txt", data.table = F)
out_glgc$PIP[!out_glgc$converge] <- 0

sumstat_GLGC <- sumstat_GLGC[sumstat_GLGC$SNP %in% out_glgc$rsid,]
pval <- 2 * pnorm(abs(sumstat_GLGC$beta / sumstat_GLGC$se), lower.tail = F)
```

In [3]:

```
out <- data.frame()
for (thr_pip in thr_pip_all) {

  xmap_3pop <- out_3pop[out_3pop$PIP > thr_pip,]
  xmap_3pop$pval <- pval[match(xmap_3pop$rsid, sumstat_GLGC$SNP)]
  xmap_3pop$pip_rep <- out_glgc$PIP[match(xmap_3pop$rsid, out_glgc$rsid)]

  xmap_eur_eas <- out_eur_eas[out_eur_eas$PIP > thr_pip,]
  xmap_eur_eas$pval <- pval[match(xmap_eur_eas$rsid, sumstat_GLGC$SNP)]
  xmap_eur_eas$pip_rep <- out_glgc$PIP[match(xmap_eur_eas$rsid, out_glgc$rsid)]

  xmap_eur_afr <- out_eur_afr[out_eur_afr$PIP > thr_pip,]
  xmap_eur_afr$pval <- pval[match(xmap_eur_afr$rsid, sumstat_GLGC$SNP)]
  xmap_eur_afr$pip_rep <- out_glgc$PIP[match(xmap_eur_afr$rsid, out_glgc$rsid)]

  xmap_afr_eas <- out_afr_eas[out_afr_eas$PIP > thr_pip,]
  xmap_afr_eas$pval <- pval[match(xmap_afr_eas$rsid, sumstat_GLGC$SNP)]
  xmap_afr_eas$pip_rep <- out_glgc$PIP[match(xmap_afr_eas$rsid, out_glgc$rsid)]

  ssx_3pop <- out_ssx_3pop[out_ssx_3pop$PIP > thr_pip,]
  ssx_3pop$pval <- pval[match(ssx_3pop$rsid, sumstat_GLGC$SNP)]
  ssx_3pop$pip_rep <- out_glgc$PIP[match(ssx_3pop$rsid, out_glgc$rsid)]

  ssx_eas_eur <- out_ssx_eas_eur[out_ssx_eas_eur$PIP > thr_pip,]
  ssx_eas_eur$pval <- pval[match(ssx_eas_eur$rsid, sumstat_GLGC$SNP)]
  ssx_eas_eur$pip_rep <- out_glgc$PIP[match(ssx_eas_eur$rsid, out_glgc$rsid)]

  ssx_eas_afr <- out_ssx_eas_afr[out_ssx_eas_afr$PIP > thr_pip,]
  ssx_eas_afr$pval <- pval[match(ssx_eas_afr$rsid, sumstat_GLGC$SNP)]
  ssx_eas_afr$pip_rep <- out_glgc$PIP[match(ssx_eas_afr$rsid, out_glgc$rsid)]

  ssx_afr_eur <- out_ssx_afr_eur[out_ssx_afr_eur$PIP > thr_pip,]
  ssx_afr_eur$pval <- pval[match(ssx_afr_eur$rsid, sumstat_GLGC$SNP)]
  ssx_afr_eur$pip_rep <- out_glgc$PIP[match(ssx_afr_eur$rsid, out_glgc$rsid)]

  susie_eur <- out_eur[out_eur$PIP > thr_pip,]
  susie_eur$pval <- pval[match(susie_eur$rsid, sumstat_GLGC$SNP)]
  susie_eur$pip_rep <- out_glgc$PIP[match(susie_eur$rsid, out_glgc$rsid)]

  susie_eas <- out_eas[out_eas$PIP > thr_pip,]
  susie_eas$pval <- pval[match(susie_eas$rsid, sumstat_GLGC$SNP)]
  susie_eas$pip_rep <- out_glgc$PIP[match(susie_eas$rsid, out_glgc$rsid)]

  susie_afr <- out_afr[out_afr$PIP > thr_pip,]
  susie_afr$pval <- pval[match(susie_afr$rsid, sumstat_GLGC$SNP)]
  susie_afr$pip_rep <- out_glgc$PIP[match(susie_afr$rsid, out_glgc$rsid)]

  susieinf_eur <- out_inf_eur[out_inf_eur$PIP > thr_pip,1:6]
  susieinf_eur$pval <- pval[match(susieinf_eur$rsid, sumstat_GLGC$SNP)]
  susieinf_eur$pip_rep <- out_glgc$PIP[match(susieinf_eur$rsid, out_glgc$rsid)]

  susieinf_eas <- out_inf_eas[out_inf_eas$PIP > thr_pip,1:6]
  susieinf_eas$pval <- pval[match(susieinf_eas$rsid, sumstat_GLGC$SNP)]
  susieinf_eas$pip_rep <- out_glgc$PIP[match(susieinf_eas$rsid, out_glgc$rsid)]

  susieinf_afr <- out_inf_afr[out_inf_afr$PIP > thr_pip,1:6]
  susieinf_afr$pval <- pval[match(susieinf_afr$rsid, sumstat_GLGC$SNP)]
  susieinf_afr$pip_rep <- out_glgc$PIP[match(susieinf_afr$rsid, out_glgc$rsid)]

  susie_3pop <- out_eur[out_eur$PIP > thr_pip |
                          out_eas$PIP > thr_pip |
                          out_afr$PIP > thr_pip,]
  susie_3pop$pval <- pval[match(susie_3pop$rsid, sumstat_GLGC$SNP)]
  susie_3pop$pip_rep <- out_glgc$PIP[match(susie_3pop$rsid, out_glgc$rsid)]

  susie_eur_eas <- out_eur[out_eur$PIP > thr_pip | out_eas$PIP > thr_pip,]
  susie_eur_eas$pval <- pval[match(susie_eur_eas$rsid, sumstat_GLGC$SNP)]
  susie_eur_eas$pip_rep <- out_glgc$PIP[match(susie_eur_eas$rsid, out_glgc$rsid)]

  susie_eur_afr <- out_eur[out_eur$PIP > thr_pip | out_afr$PIP > thr_pip,]
  susie_eur_afr$pval <- pval[match(susie_eur_afr$rsid, sumstat_GLGC$SNP)]
  susie_eur_afr$pip_rep <- out_glgc$PIP[match(susie_eur_afr$rsid, out_glgc$rsid)]

  susie_afr_eas <- out_eur[out_eas$PIP > thr_pip | out_afr$PIP > thr_pip,]
  susie_afr_eas$pval <- pval[match(susie_afr_eas$rsid, sumstat_GLGC$SNP)]
  susie_afr_eas$pip_rep <- out_glgc$PIP[match(susie_afr_eas$rsid, out_glgc$rsid)]

  out <- rbind(out,
               data.frame(xmap_3pop, method = "XMAP", Population = "EUR+AFR+EAS", thr_pip = thr_pip),
               data.frame(xmap_eur_afr, method = "XMAP", Population = "EUR+AFR", thr_pip = thr_pip),
               data.frame(xmap_eur_eas, method = "XMAP", Population = "EUR+EAS", thr_pip = thr_pip),
               data.frame(xmap_afr_eas, method = "XMAP", Population = "AFR+EAS", thr_pip = thr_pip),
               data.frame(ssx_3pop, method = "SuSiEx", Population = "EUR+AFR+EAS", thr_pip = thr_pip),
               data.frame(ssx_afr_eur, method = "SuSiEx", Population = "EUR+AFR", thr_pip = thr_pip),
               data.frame(ssx_eas_eur, method = "SuSiEx", Population = "EUR+EAS", thr_pip = thr_pip),
               data.frame(ssx_eas_afr, method = "SuSiEx", Population = "AFR+EAS", thr_pip = thr_pip),
               data.frame(susie_eur, method = "SuSiE", Population = "EUR", thr_pip = thr_pip),
               data.frame(susie_eas, method = "SuSiE", Population = "EAS", thr_pip = thr_pip),
               data.frame(susie_afr, method = "SuSiE", Population = "AFR", thr_pip = thr_pip),
               data.frame(susieinf_eur, method = "SuSiE-inf", Population = "EUR", thr_pip = thr_pip),
               data.frame(susieinf_eas, method = "SuSiE-inf", Population = "EAS", thr_pip = thr_pip),
               data.frame(susieinf_afr, method = "SuSiE-inf", Population = "AFR", thr_pip = thr_pip),
               data.frame(susie_3pop, method = "SuSiE-merge", Population = "EUR+AFR+EAS", thr_pip = thr_pip),
               data.frame(susie_eur_afr, method = "SuSiE-merge", Population = "EUR+AFR", thr_pip = thr_pip),
               data.frame(susie_eur_eas, method = "SuSiE-merge", Population = "EUR+EAS", thr_pip = thr_pip),
               data.frame(susie_afr_eas, method = "SuSiE-merge", Population = "AFR+EAS", thr_pip = thr_pip))
  cat(thr_pip, " finished.\n")
}
```

```
0.8  finished.
0.9  finished.
0.95  finished.
0.99  finished.
```

In [4]:

```
out$sig <- factor(ifelse(out$pval < 5e-8, "< 5e-8", "> 5e-8"),
                  levels = c("> 5e-8", "< 5e-8"))
out$piprange <- ifelse(out$pip_rep > 0.1, ">0.1", "<0.1")
out$piprange[out$pip_rep > 0.1 & out$pip_rep < 0.5] <- "0.1-0.5"
out$piprange[out$pip_rep > 0.5 & out$pip_rep < 0.9] <- "0.5-0.9"
out$piprange[out$pip_rep > 0.9] <- ">0.9"
out$piprange <- factor(out$piprange, levels = c("<0.1", "0.1-0.5", "0.5-0.9", ">0.9"))
out$thr_pip <- paste0("PIP>", out$thr_pip)
```

In [5]:

```
options(repr.plot.width=18, repr.plot.height=6)
ggplot(out, aes(x = method, fill = piprange)) +
  geom_bar() +
  geom_text(stat = 'count', aes(label = ..count..), position = position_stack(vjust = 0.5)) +
  # scale_y_continuous(labels = scales::percent) +
  ylab("Number of causal SNPs") +
  facet_grid(Population ~ thr_pip, scales = "free_y", space = 'free') +
  scale_fill_brewer(palette = "Paired") +
  theme_bw() +
  coord_flip() +
  guides(fill = guide_legend(title = "PIP in sibship GWAS")) +
  theme(legend.position = "bottom")
# 2000 * 800
```

```
Warning message:
“The dot-dot notation (`..count..`) was deprecated in ggplot2 3.4.0.
ℹ Please use `after_stat(count)` instead.”
```
