# Supplementary material for: XMAP: Cross-population fine-mapping by leveraging genetic diversity and accounting for confounding bias
Source: Nat Commun. 2023 Oct 28;14:6870. doi: 10.1038/s41467-023-42614-7 (PMC10613261; doi:10.1038/s41467-023-42614-7)
Supplement: Supplementary file 5 — Reporting Summary [file 41467_2023_42614_MOESM5_ESM.pdf]

nature portfolio

Corresponding author(s): YANG Can
CAI Mingxuan

Last updated by author(s): Sep 17, 2023

Reporting Summary

Nature Portfolio wishes to improve the reproducibility of the work that we publish. This form provides structure for consistency and transparency in reporting. For further information on Nature Portfolio policies, see our Editorial Policies and the Editorial Policy Checklist.

Statistics

For all statistical analyses, confirm that the following items are present in the figure legend, table legend, main text, or Methods section.

n/a	Confirmed	
☐	☒	The exact sample size (*n*) for each experimental group/condition, given as a discrete number and unit of measurement
☒	☐	A statement on whether measurements were taken from distinct samples or whether the same sample was measured repeatedly
☐	☒	The statistical test(s) used AND whether they are one- or two-sided<br>*Only common tests should be described solely by name; describe more complex techniques in the Methods section.*
☐	☒	A description of all covariates tested
☐	☒	A description of any assumptions or corrections, such as tests of normality and adjustment for multiple comparisons
☐	☒	A full description of the statistical parameters including central tendency (e.g. means) or other basic estimates (e.g. regression coefficient) AND variation (e.g. standard deviation) or associated estimates of uncertainty (e.g. confidence intervals)
☐	☒	For null hypothesis testing, the test statistic (e.g. *F*, *t*, *r*) with confidence intervals, effect sizes, degrees of freedom and *P* value noted<br>*Give P values as exact values whenever suitable.*
☐	☒	For Bayesian analysis, information on the choice of priors and Markov chain Monte Carlo settings
☐	☒	For hierarchical and complex designs, identification of the appropriate level for tests and full reporting of outcomes
☒	☐	Estimates of effect sizes (e.g. Cohen's *d*, Pearson's *r*), indicating how they were calculated

Our web collection on statistics for biologists contains articles on many of the points above.

Software and code

Policy information about availability of computer code

Data collection

The present study does not involve data collection. We used publicly available data sets to validate the effectiveness of our proposed method. No software was used for data collection.

Data analysis

We used the newly developed R package XMAP for data analysis. XMAP is in the Methods section and deposited at GitHub (https://github.com/YangLabHKUST/XMAP). The source code is released under the GNU General Public License version 3 (GPL>=3). Example codes for using XMAP are publicly available at https://mxcai.github.io/XMAP-tutorial/index.html. All analysis codes for reproducing the results of the present study are publicly available at https://github.com/YangLabHKUST/XMAP/tree/main/results. The R codes for simulation analyses are also available at https://github.com/YangLabHKUST/XMAP/tree/main/results/simulation.

We used the following software packages for comparative analysis:
PAINTOR [https://github.com/gkichaev/PAINTOR_V3.0] (Python package v3.0)
MsCAVIAR [https://github.com/nlapier2/MsCAVIAR]
FINEMAP [http://www.christianbenner.com] (v1.4.1)
SuSiE [https://github.com/stephenslab/susieR] (R package v0.12.06)
SuSiE-inf [https://github.com/FinucaneLab/fine-mapping-inf] (R package v1.0)
SuSiEx [https://github.com/getian107/SuSiEx] (R package v1.0.0)
DAP-G [https://github.com/xqwen/dap] (R package v1.0.0)

In addtion, we used the following packages for data analysis:
g-chromVAR [https://github.com/caleblareau/gchromVAR] (R pacakage 0.3.2)

SCAVENGE [https://github.com/sankaranlab/SCAVENGE] (R pacakage 1.0.1)
Seurat [https://github.com/satijalab/seurat] (R package v 4.0.3)

For manuscripts utilizing custom algorithms or software that are central to the research but not yet described in published literature, software must be made available to editors and reviewers. We strongly encourage code deposition in a community repository (e.g. GitHub). See the Nature Portfolio guidelines for submitting code & software for further information.

Data

Policy information about availability of data

All manuscripts must include a data availability statement. This statement should provide the following information, where applicable:

- Accession codes, unique identifiers, or web links for publicly available datasets
- A description of any restrictions on data availability
- For clinical datasets or third party data, please ensure that the statement adheres to our policy

The study made use of publicly available datasets. Summary statistics of LDL from GLGC can be downloaded at http://csg.sph.umich.edu/willer/public/glgc-lipids2021/. Summary statistics of LDL, height and blood traits from UKBB are available at https://nealelab.github.io/UKBB_ldsc/index.html. Summary statistics of height and blood traits from BBJ are available at http://jenger.riken.jp/en/result. Summary statistics from the within-sibship GWAS are available at https://gwas.mrcieu.ac.uk. The details of publicly available GWAS summary statistics are summarized in Supplementary Table 2. LD files for UKBB British-ancestry and African samples are available at https://data.broadinstitute.org/alkesgroup/UKBB_LD. The scATAC-seq dataset is available at https://github.com/GreenleafLab/MPAL-Single-Cell-2019. The fine-mapping results generated in this study have been deposited in https://github.com/YangLabHKUST/XMAP/tree/main/results.

Research involving human participants, their data, or biological material

Policy information about studies with human participants or human data. See also policy information about sex, gender (identity/presentation), and sexual orientation and race, ethnicity and racism.

Reporting on sex and gender	N/A
Reporting on race, ethnicity, or other socially relevant groupings	N/A
Population characteristics	N/A
Recruitment	N/A
Ethics oversight	N/A

Note that full information on the approval of the study protocol must also be provided in the manuscript.

Field-specific reporting

Please select the one below that is the best fit for your research. If you are not sure, read the appropriate sections before making your selection.

[x] Life sciences [ ] Behavioural & social sciences [ ] Ecological, evolutionary & environmental sciences

For a reference copy of the document with all sections, see nature.com/documents/nr-reporting-summary-flat.pdf

Life sciences study design

All studies must disclose on these points even when the disclosure is negative.

Sample size	In simulation studies, we generated 20,000 samples from European population and considered sample sizes of 5,000, 10,000, 15,000, and 20,000 for the East Asian population to mimic the unbalanced composition of GWAS samples across global populations.<br><br>For GWAS summary statistics, we used data downloaded from public websites and used all samples in these data. The sample size determination information, if available, were described in the original paper. These original papers are cited in the present study and summarized in Supplementary Table 1 and 2.
Data exclusions	All data are included.
Replication	In our paper, we proposed an approach to improve cross-population fine-mapping. All data are public available and we do not perform any experiment, so replication is not relevant to our study.
Randomization	In our paper, we proposed an approach to improve cross-population fine-mapping. All data are public available and we do not perform any experiment, so randomization is not relevant to our study.
Blinding	In our paper, we proposed an approach to improve cross-population fine-mapping. All data are public available and we do not perform any

Blinding	experiment, so blinding is not relevant to our study.

Reporting for specific materials, systems and methods

We require information from authors about some types of materials, experimental systems and methods used in many studies. Here, indicate whether each material, system or method listed is relevant to your study. If you are not sure if a list item applies to your research, read the appropriate section before selecting a response.

Materials & experimental systems

n/a	Involved in the study
☒	☐ Antibodies
☒	☐ Eukaryotic cell lines
☒	☐ Palaeontology and archaeology
☒	☐ Animals and other organisms
☒	☐ Clinical data
☒	☐ Dual use research of concern
☒	☐ Plants

Methods

n/a	Involved in the study
☒	☐ ChIP-seq
☒	☐ Flow cytometry
☒	☐ MRI-based neuroimaging
